# Supplementary figures and images for: Fractionation of Lycopodiaceae Alkaloids and Evaluation of Their Anticholinesterase and Cytotoxic Activities
Source: Molecules. 2021 Oct 22;26(21):6379. doi: 10.3390/molecules26216379 (PMC8588253; doi:10.3390/molecules26216379)

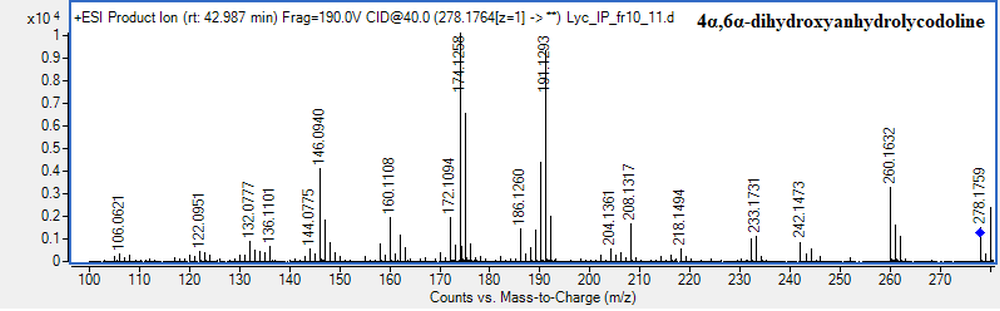

Supplement: Supplementary file 1 [file molecules-26-06379-s001.zip › Fig.S2.a.tif]

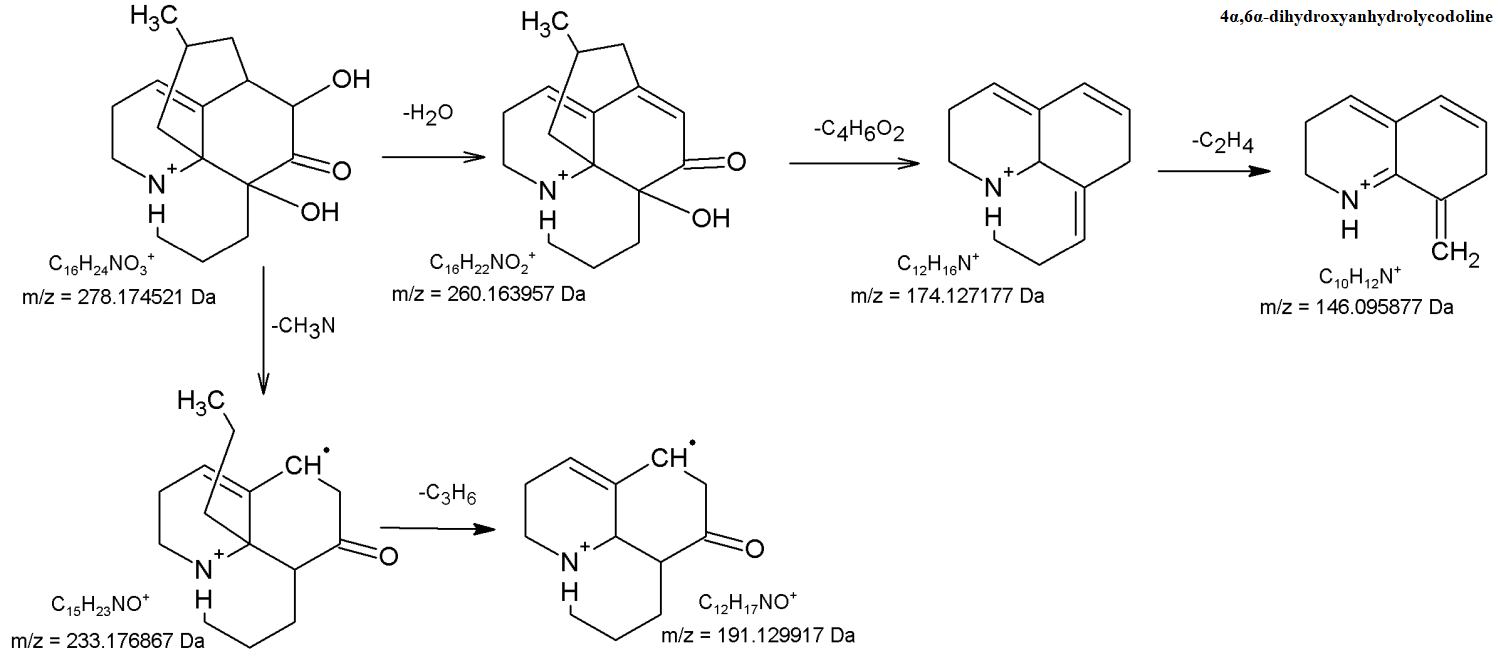

Supplement: Supplementary file 1 [file molecules-26-06379-s001.zip › Fig.S2.b.tif]

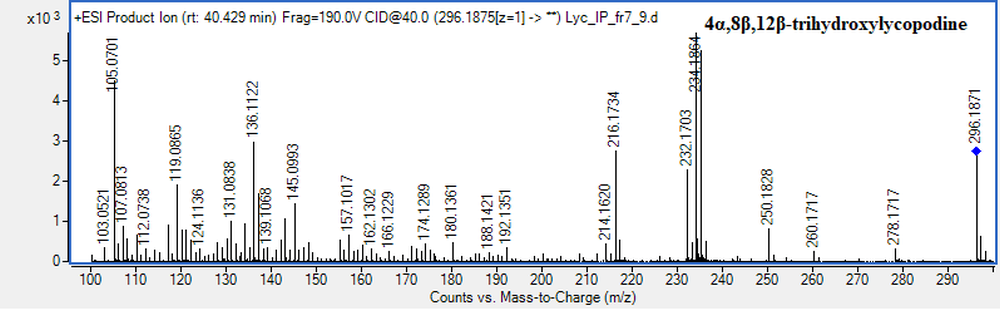

Supplement: Supplementary file 1 [file molecules-26-06379-s001.zip › Fig.S3.a.tif]

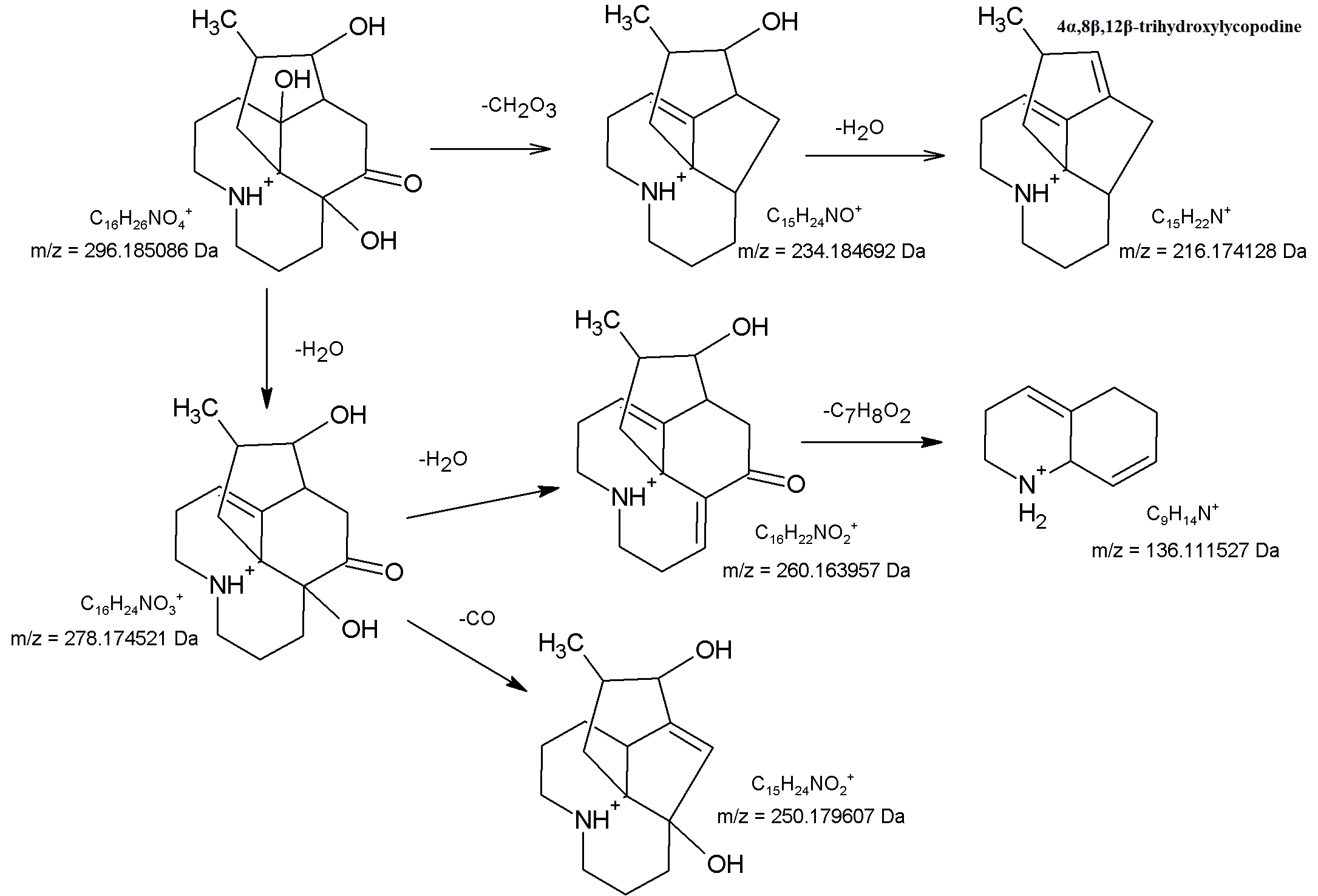

Supplement: Supplementary file 1 [file molecules-26-06379-s001.zip › Fig.S3.b.tif]

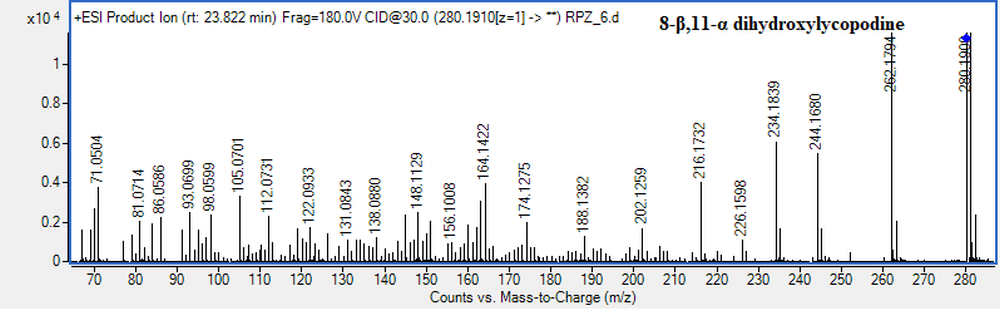

Supplement: Supplementary file 1 [file molecules-26-06379-s001.zip › Fig.S4.a.tif]

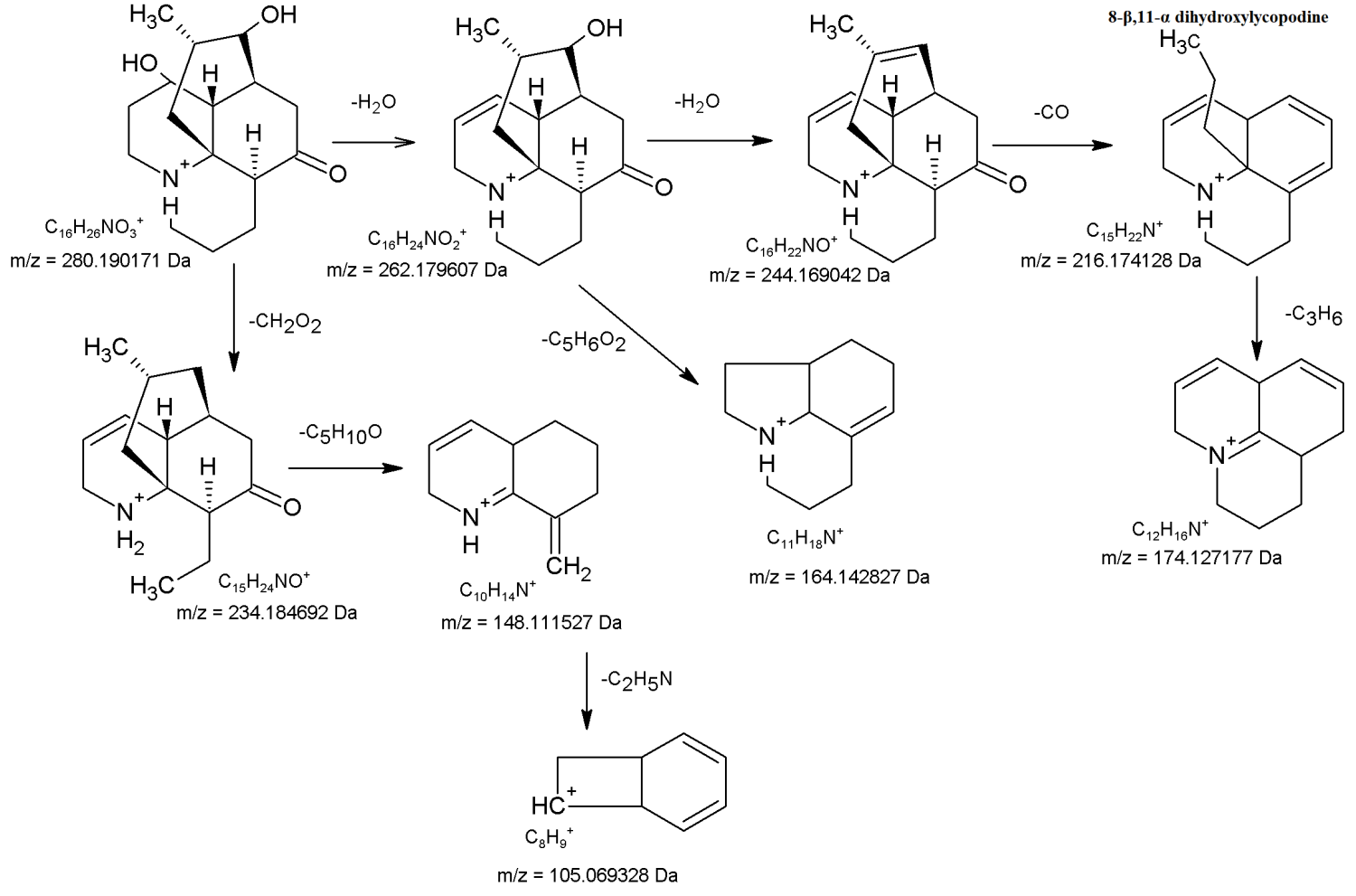

Supplement: Supplementary file 1 [file molecules-26-06379-s001.zip › Fig.S4.b.tif]

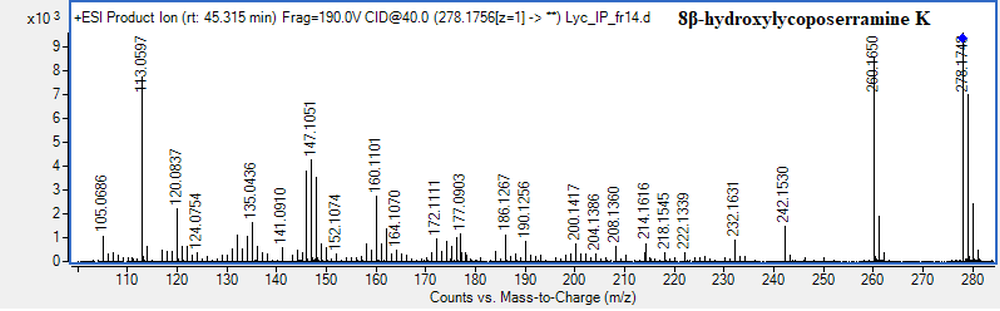

Supplement: Supplementary file 1 [file molecules-26-06379-s001.zip › Fig.S5.a.tif]

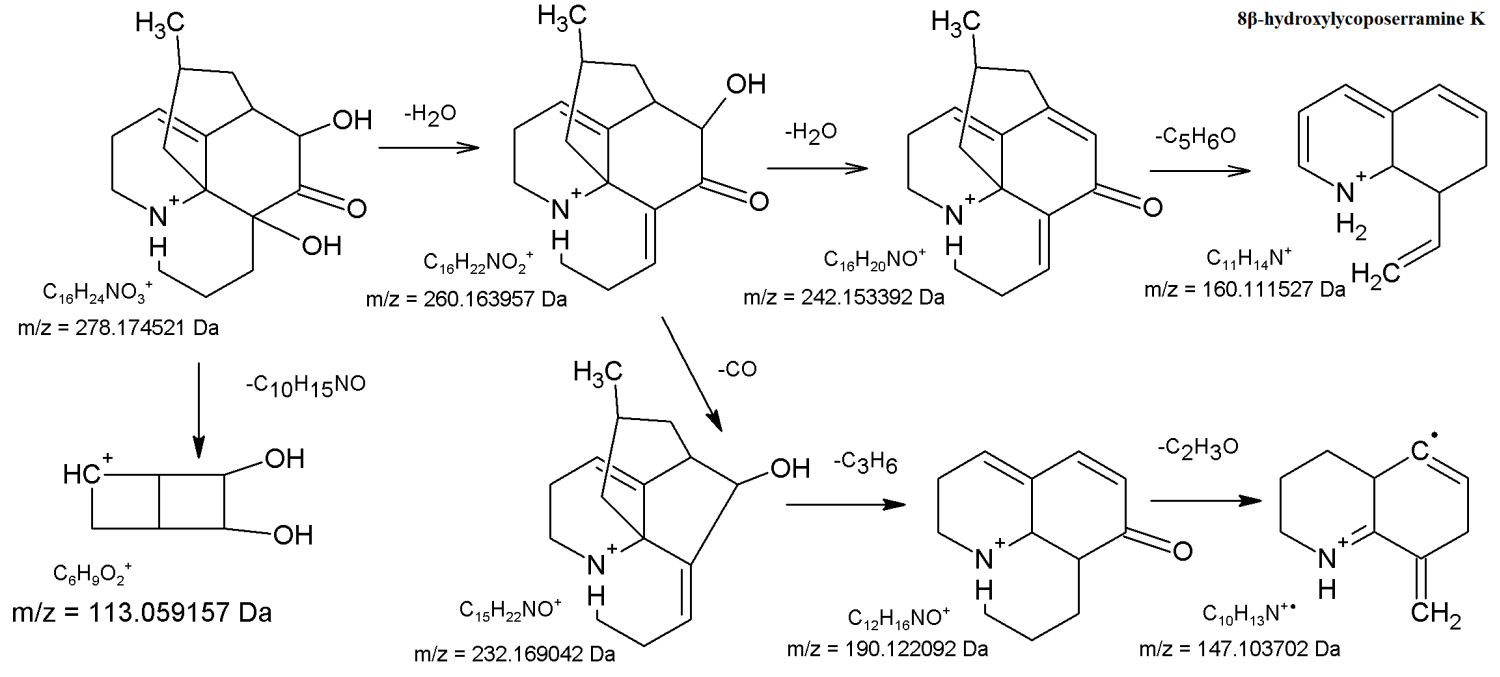

Supplement: Supplementary file 1 [file molecules-26-06379-s001.zip › Fig.S5.b.tif]

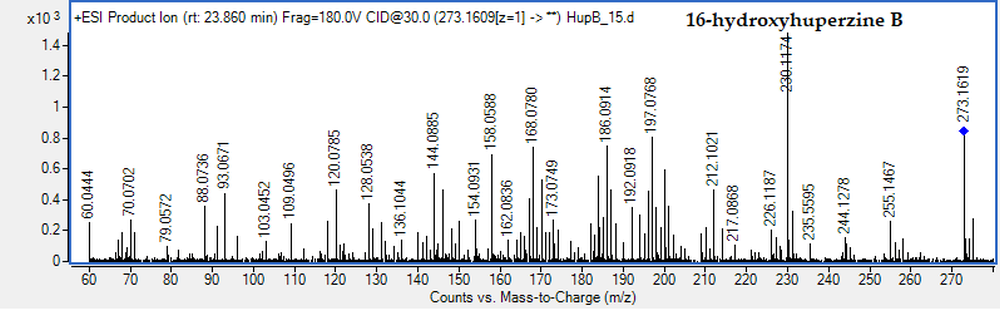

Supplement: Supplementary file 1 [file molecules-26-06379-s001.zip › Fig.S6.a.tif]

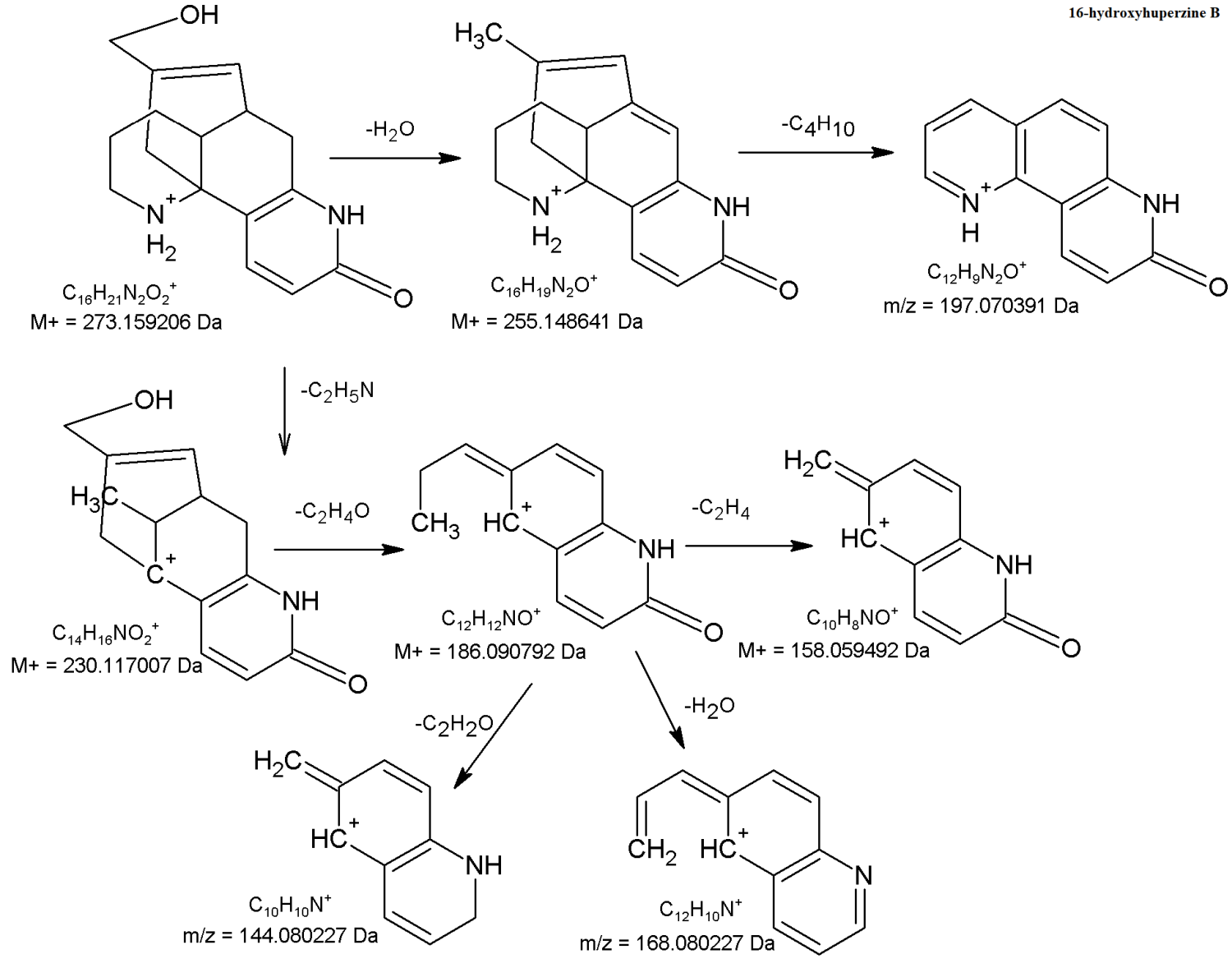

Supplement: Supplementary file 1 [file molecules-26-06379-s001.zip › Fig.S6.b.tif]

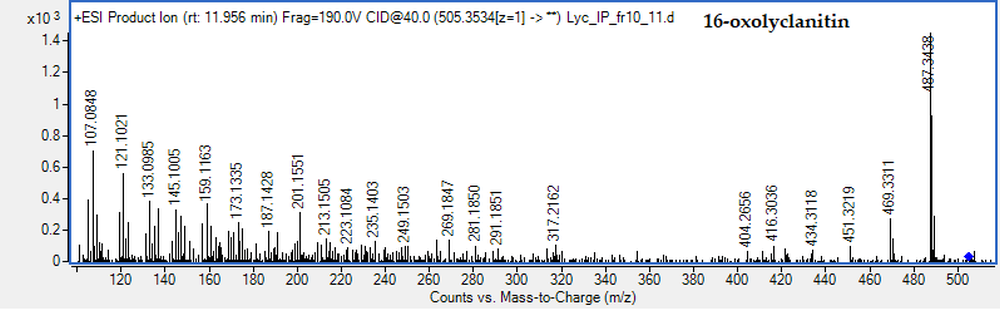

Supplement: Supplementary file 1 [file molecules-26-06379-s001.zip › Fig.S7.a.tif]

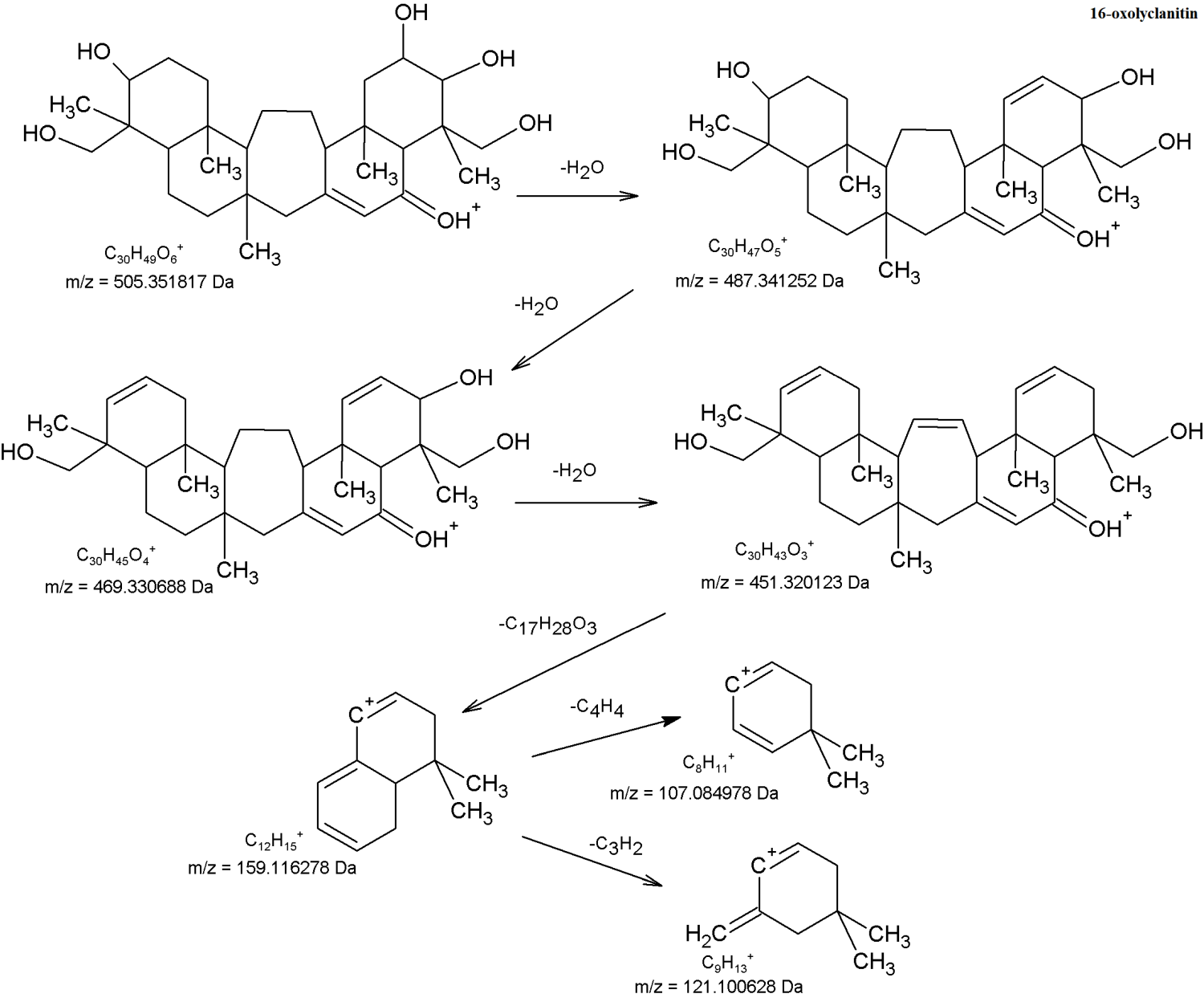

Supplement: Supplementary file 1 [file molecules-26-06379-s001.zip › Fig.S7.b.tif]

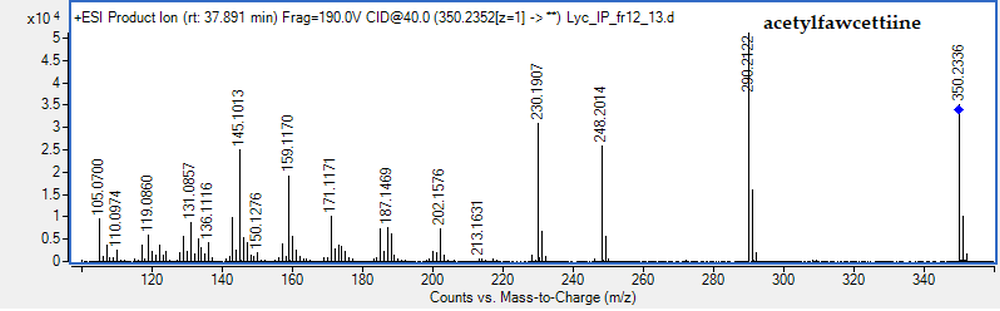

Supplement: Supplementary file 1 [file molecules-26-06379-s001.zip › Fig.S8.a.tif]

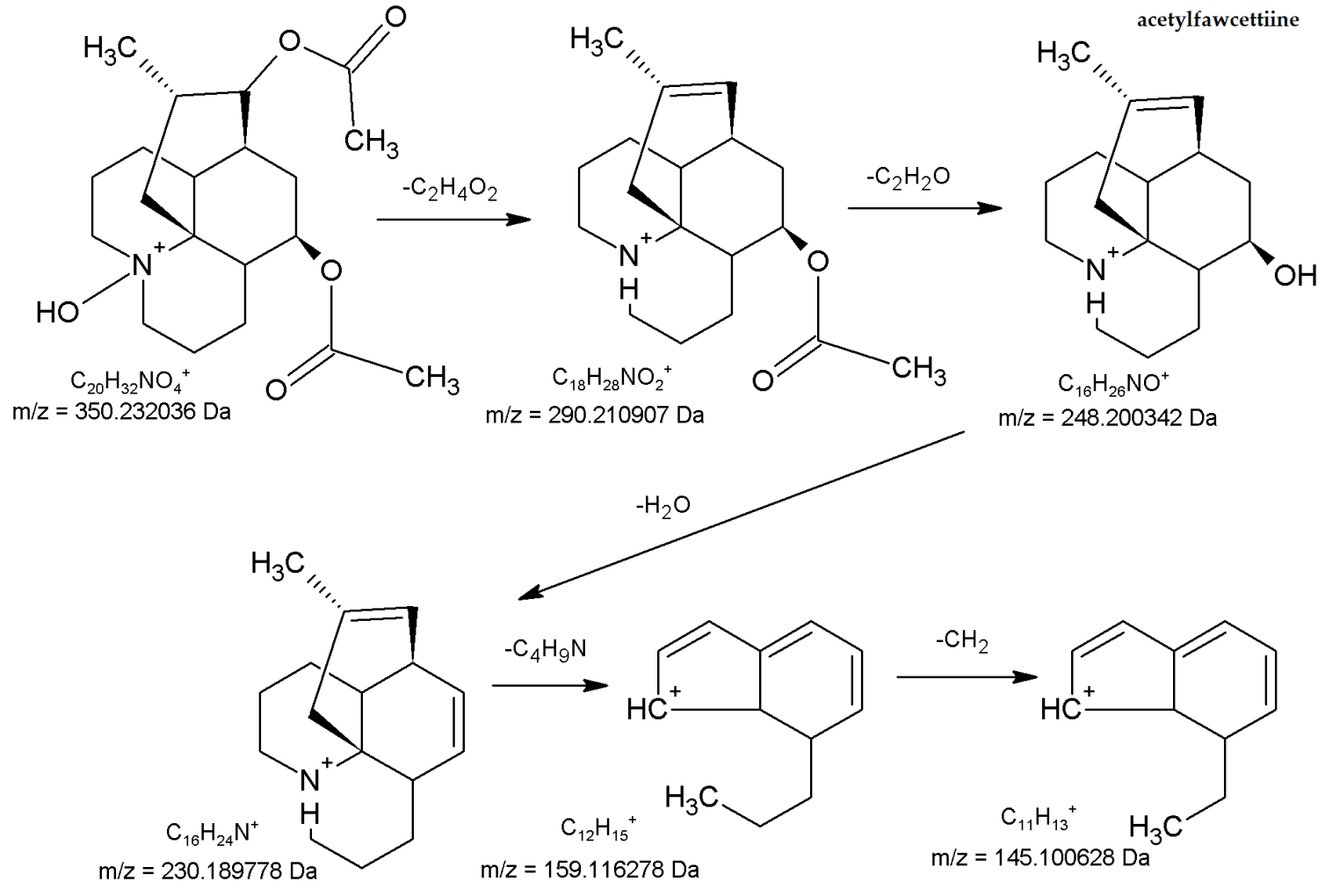

Supplement: Supplementary file 1 [file molecules-26-06379-s001.zip › Fig.S8.b.tif]

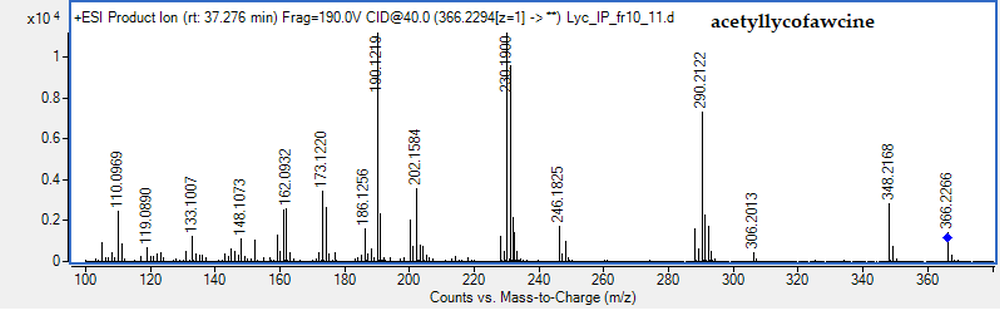

Supplement: Supplementary file 1 [file molecules-26-06379-s001.zip › Fig.S9.a.tif]

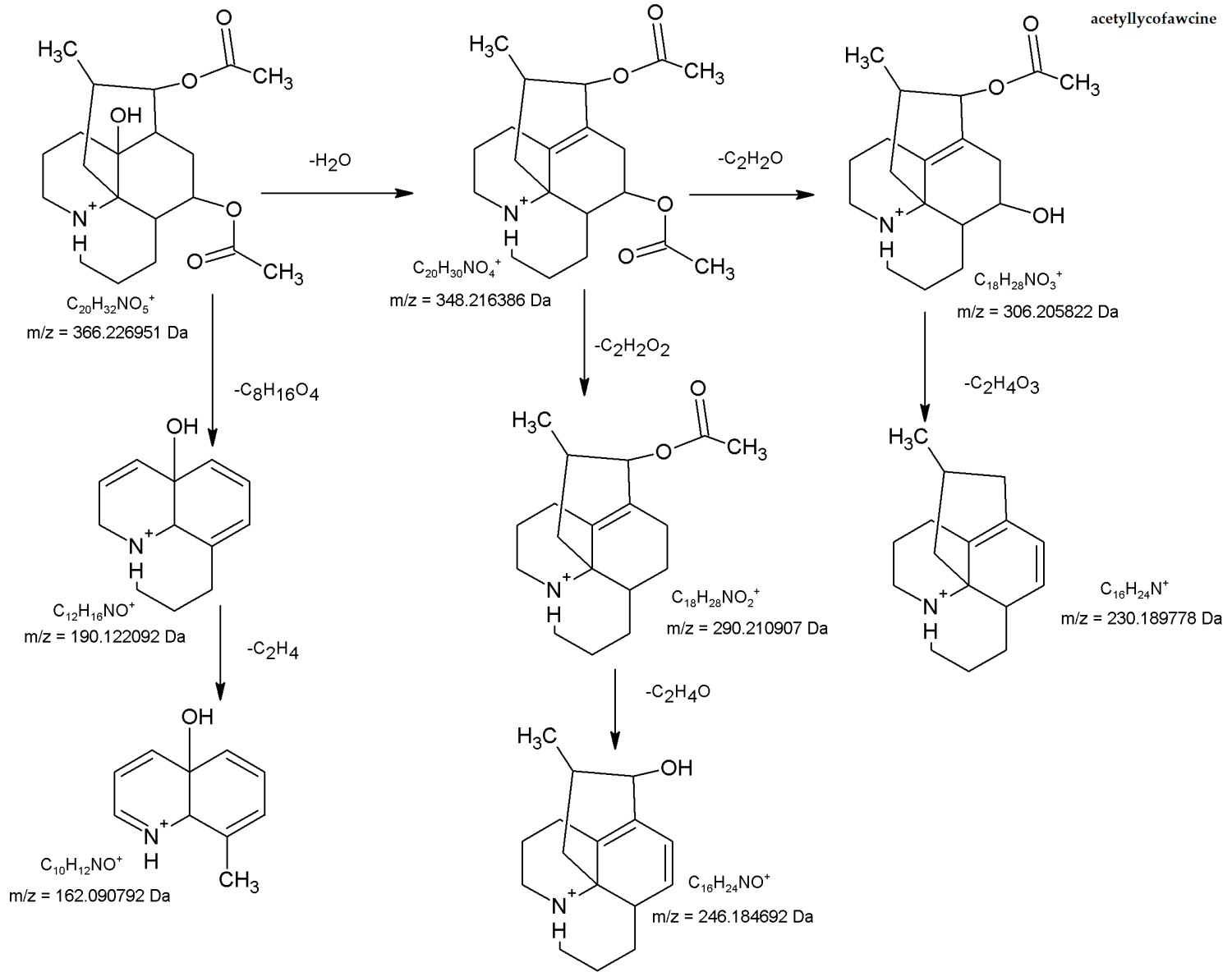

Supplement: Supplementary file 1 [file molecules-26-06379-s001.zip › Fig.S9.b.tif]

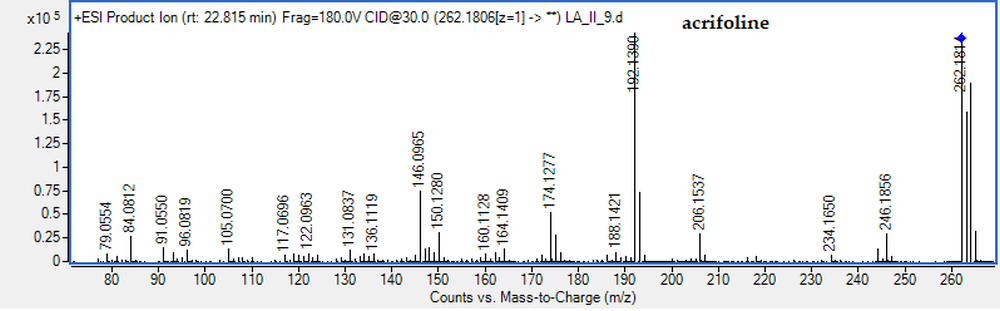

Supplement: Supplementary file 1 [file molecules-26-06379-s001.zip › Fig.S10.a.tif]

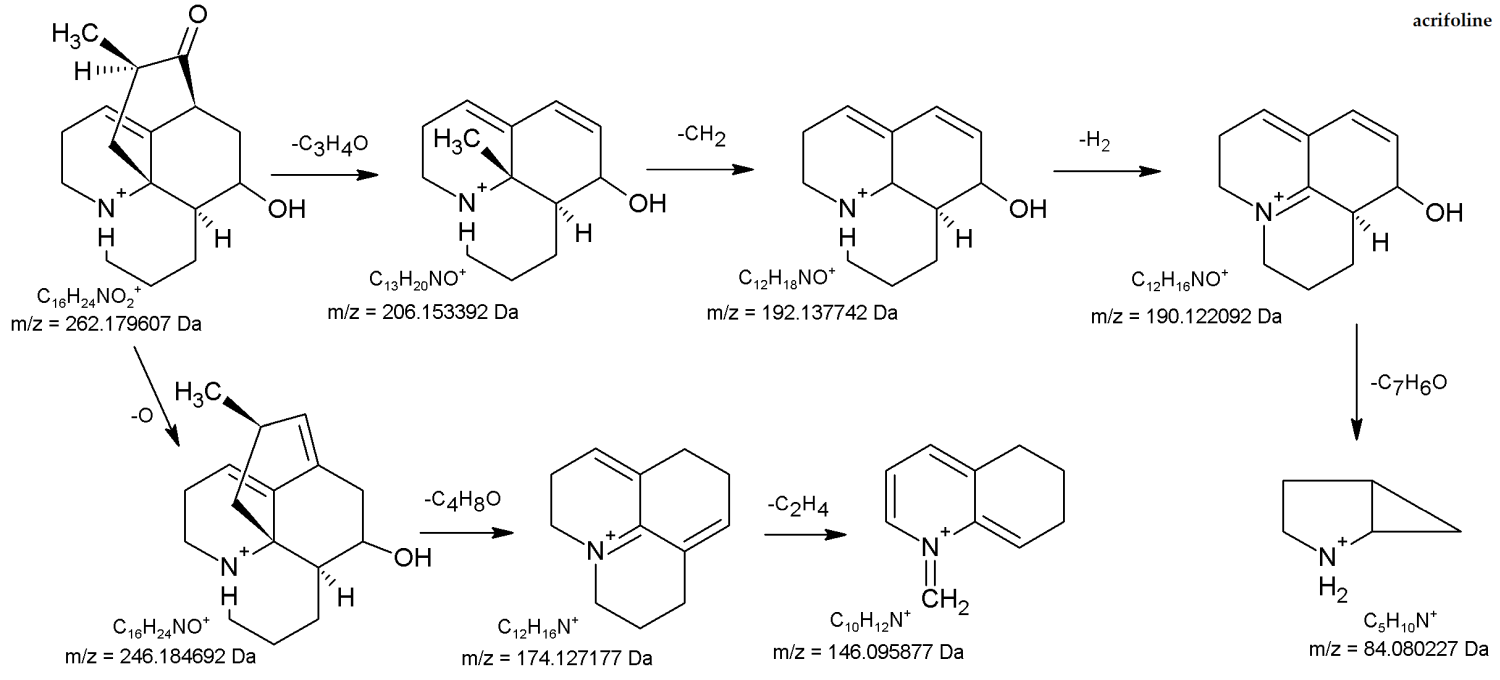

Supplement: Supplementary file 1 [file molecules-26-06379-s001.zip › Fig.S10.b.tif]

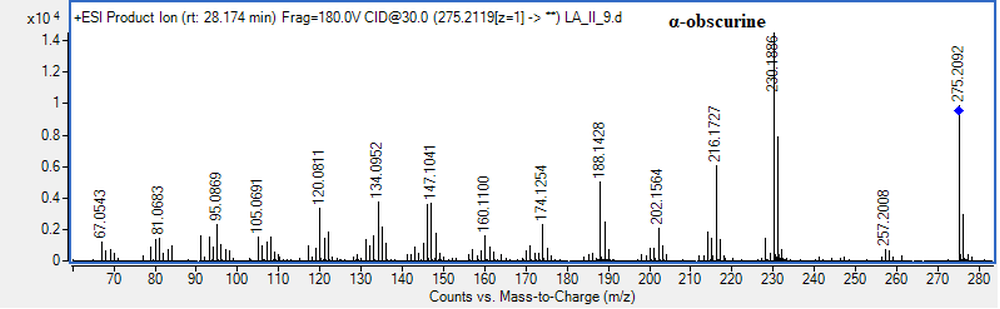

Supplement: Supplementary file 1 [file molecules-26-06379-s001.zip › Fig.S11.a.tif]

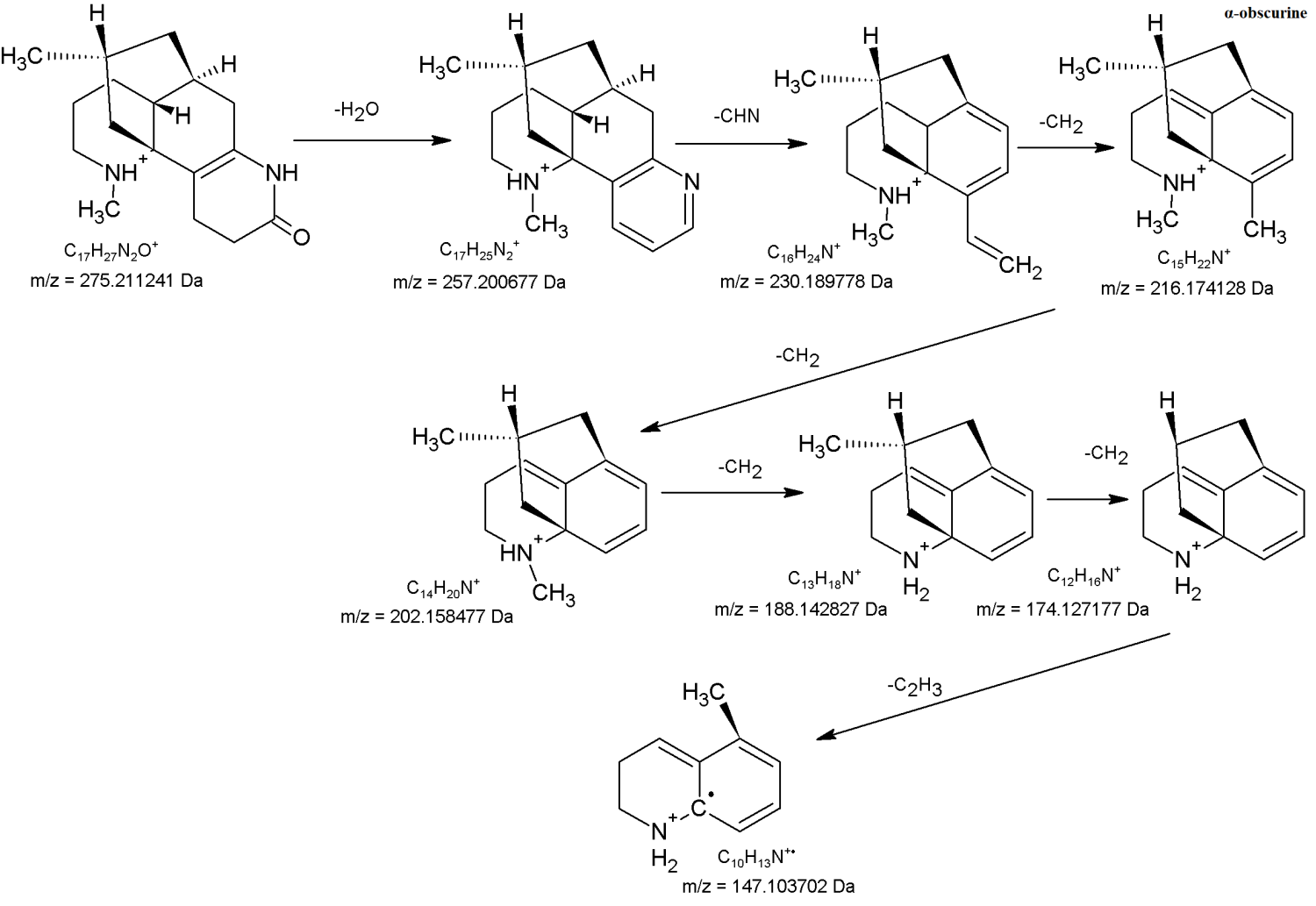

Supplement: Supplementary file 1 [file molecules-26-06379-s001.zip › Fig.S11.b.tif]

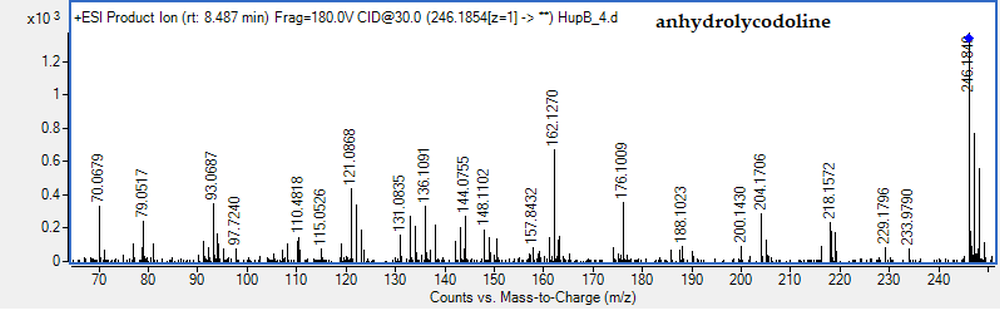

Supplement: Supplementary file 1 [file molecules-26-06379-s001.zip › Fig.S12.a.tif]

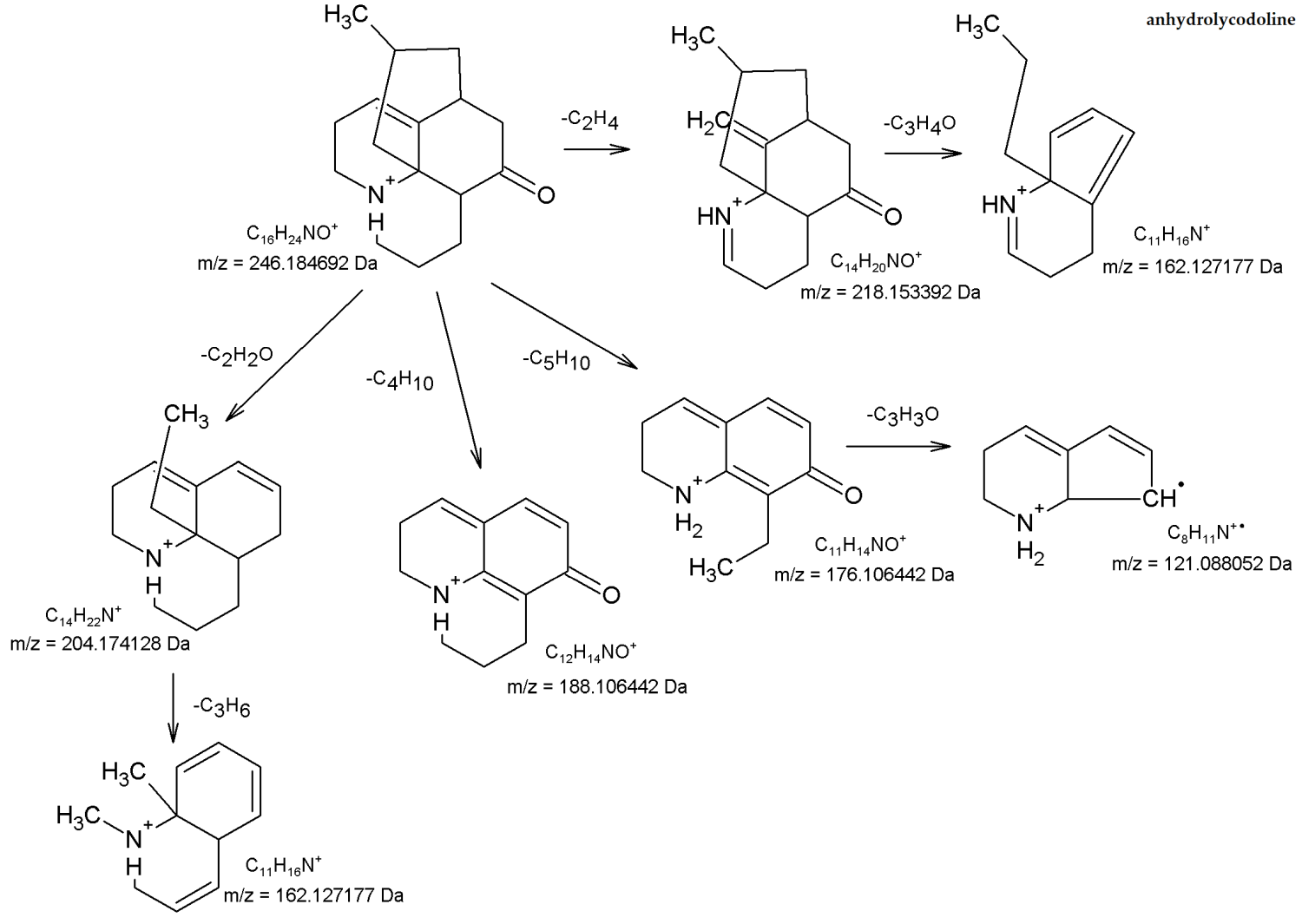

Supplement: Supplementary file 1 [file molecules-26-06379-s001.zip › Fig.S12.b.tif]

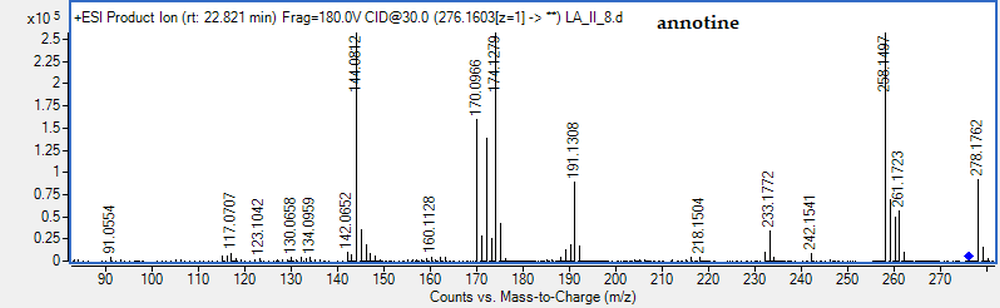

Supplement: Supplementary file 1 [file molecules-26-06379-s001.zip › Fig.S13.a.tif]

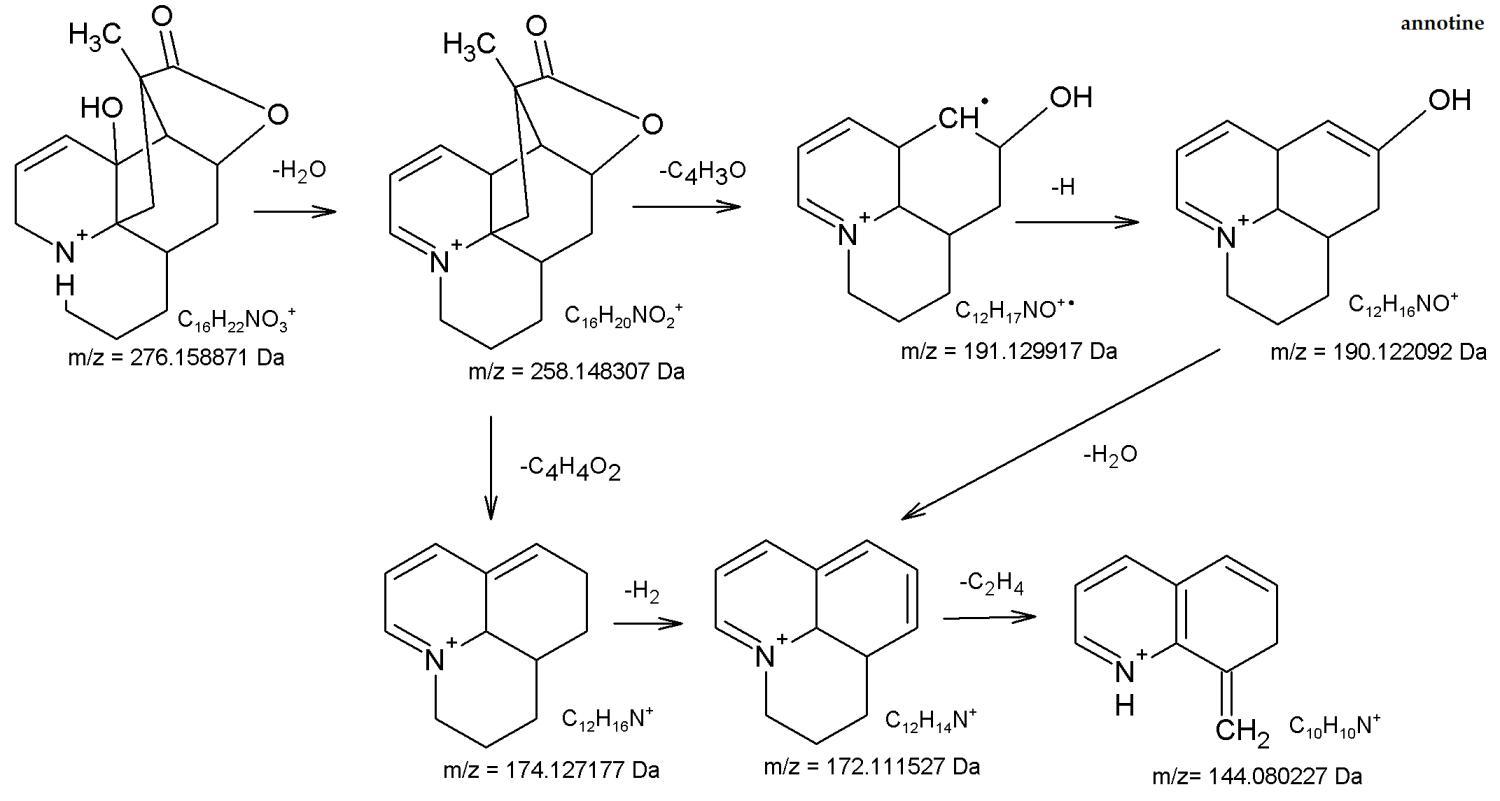

Supplement: Supplementary file 1 [file molecules-26-06379-s001.zip › Fig.S13.b.tif]

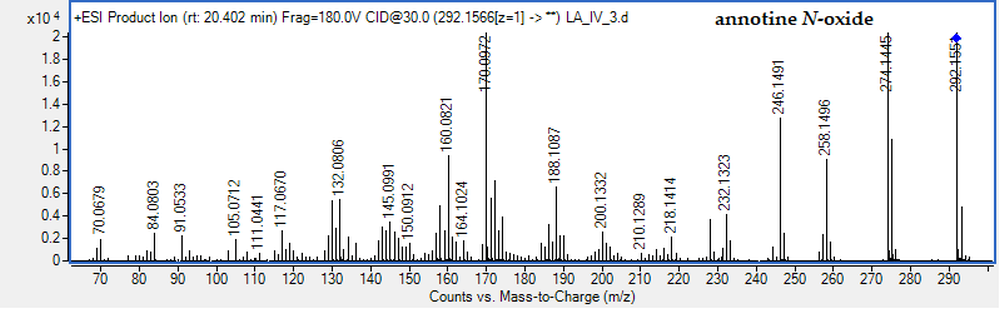

Supplement: Supplementary file 1 [file molecules-26-06379-s001.zip › Fig.S14.a.tif]

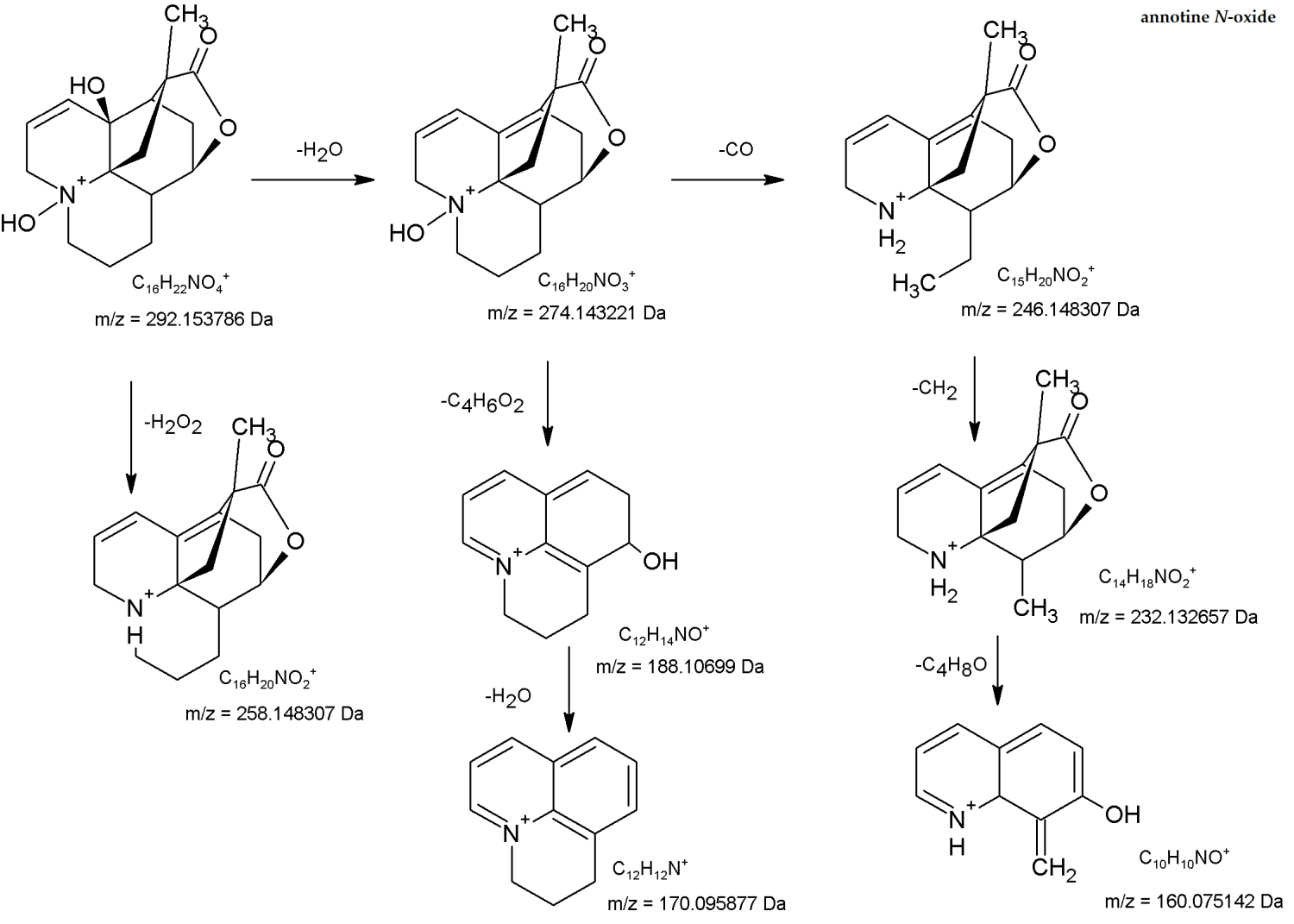

Supplement: Supplementary file 1 [file molecules-26-06379-s001.zip › Fig.S14.b.tif]

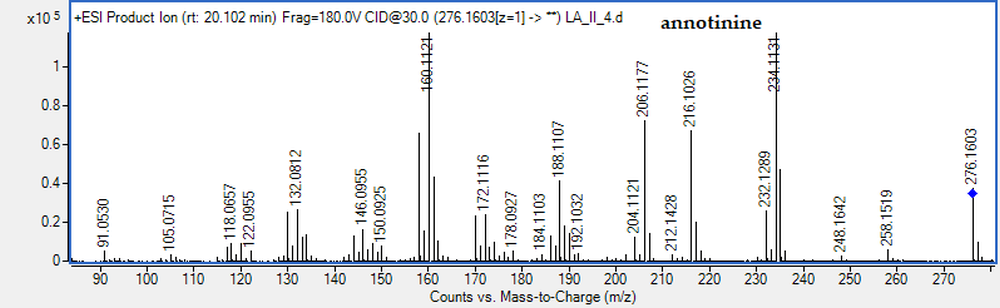

Supplement: Supplementary file 1 [file molecules-26-06379-s001.zip › Fig.S15.a.tif]

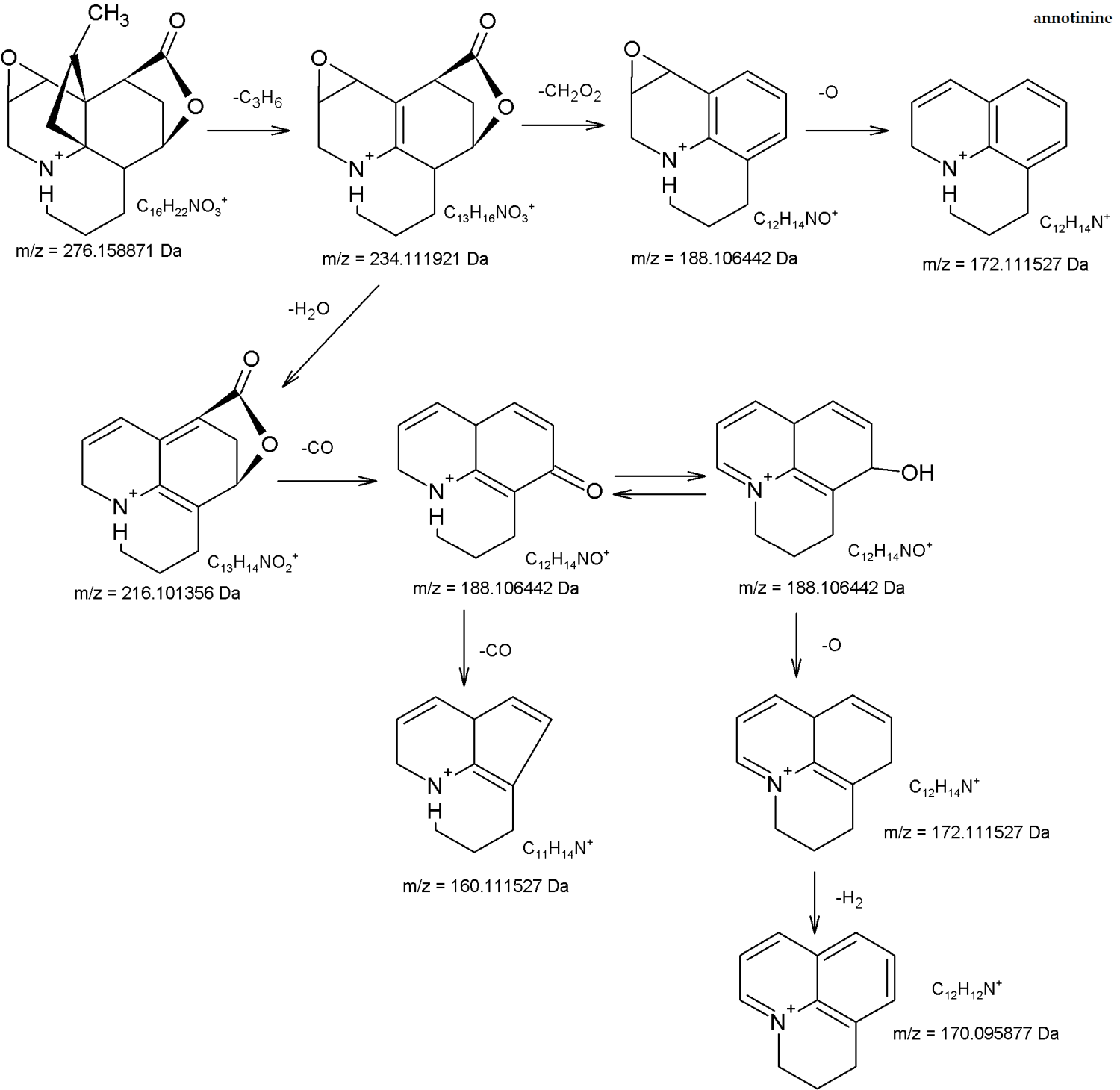

Supplement: Supplementary file 1 [file molecules-26-06379-s001.zip › Fig.S15.b.tif]

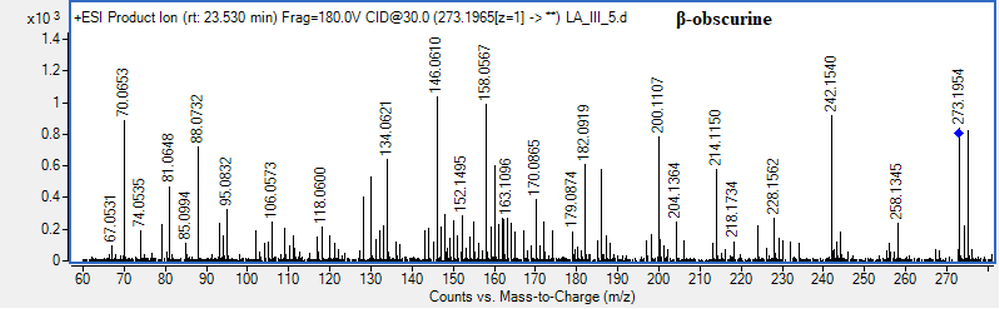

Supplement: Supplementary file 1 [file molecules-26-06379-s001.zip › Fig.S16.a.tif]

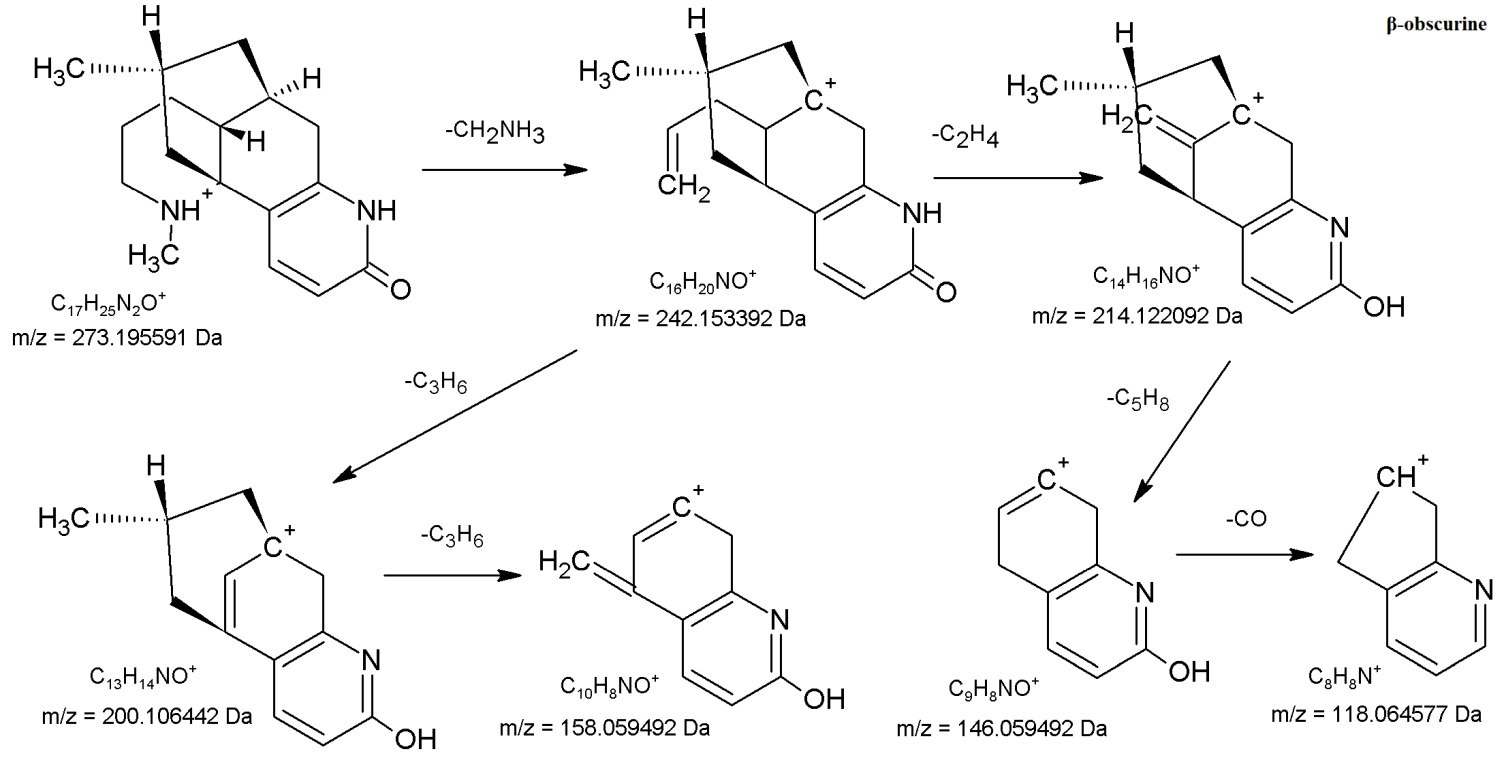

Supplement: Supplementary file 1 [file molecules-26-06379-s001.zip › Fig.S16.b.tif]

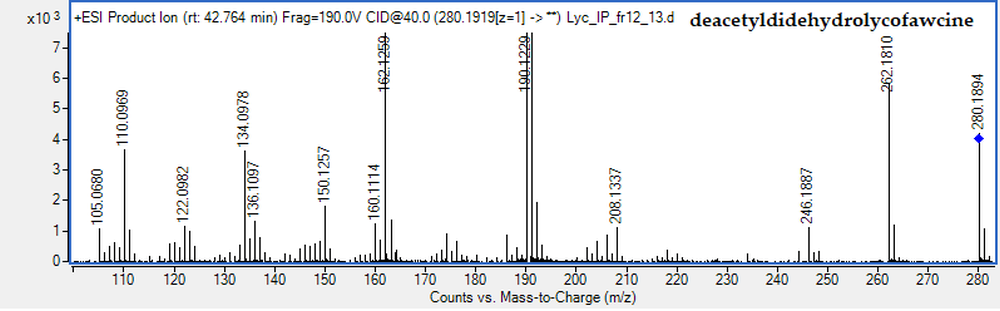

Supplement: Supplementary file 1 [file molecules-26-06379-s001.zip › Fig.S17.a.tif]

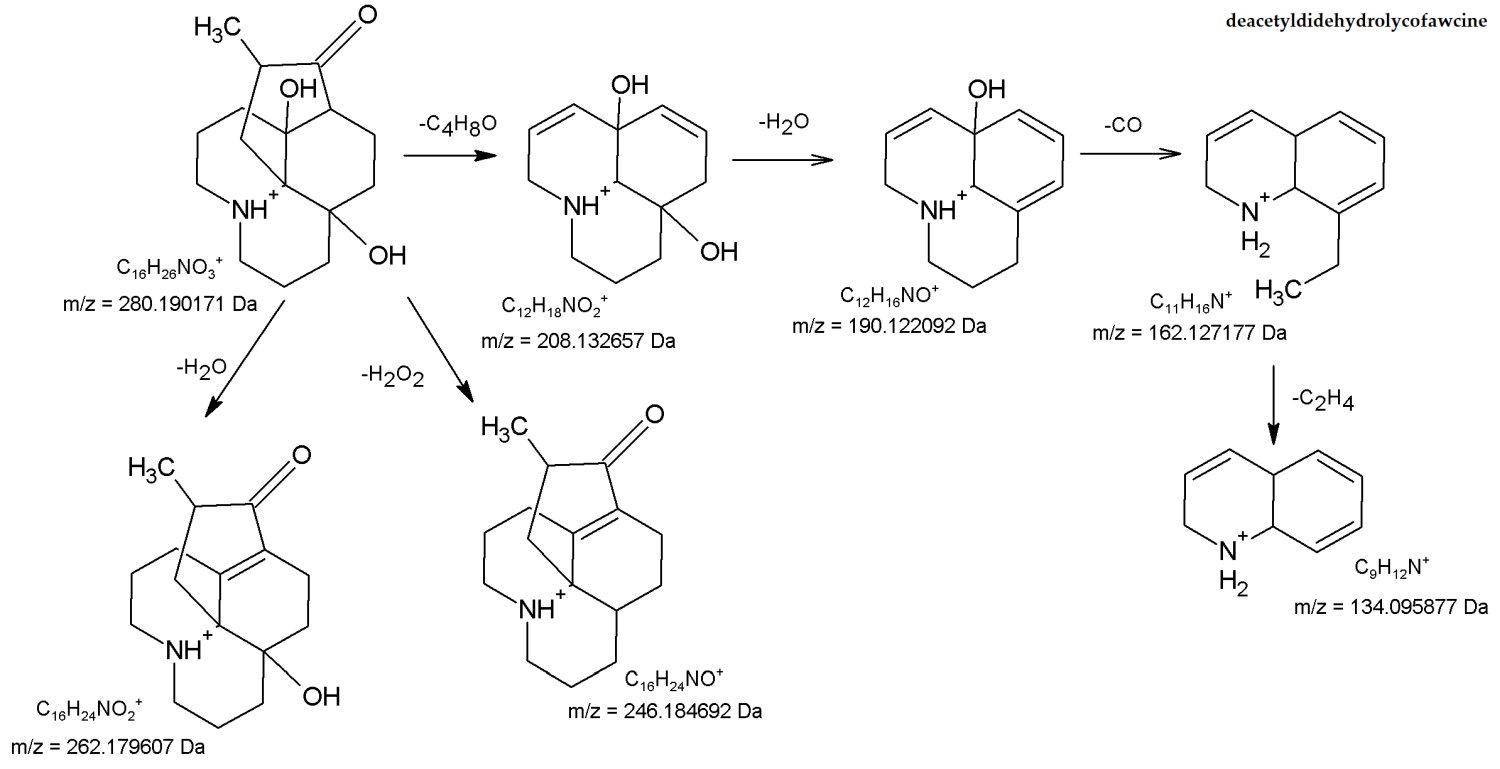

Supplement: Supplementary file 1 [file molecules-26-06379-s001.zip › Fig.S17.b.tif]

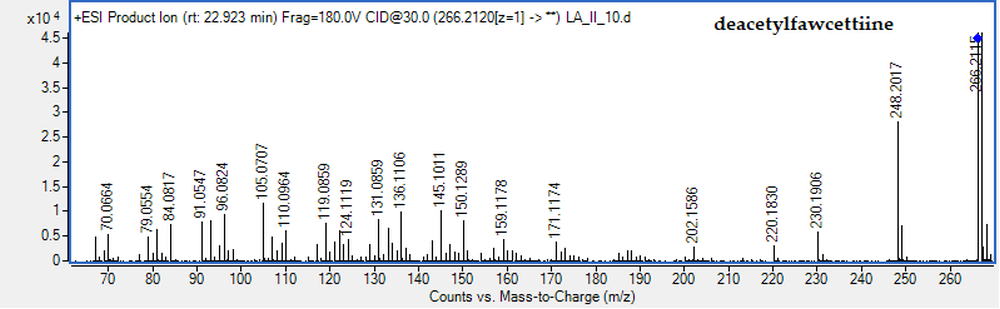

Supplement: Supplementary file 1 [file molecules-26-06379-s001.zip › Fig.S18.a.tif]

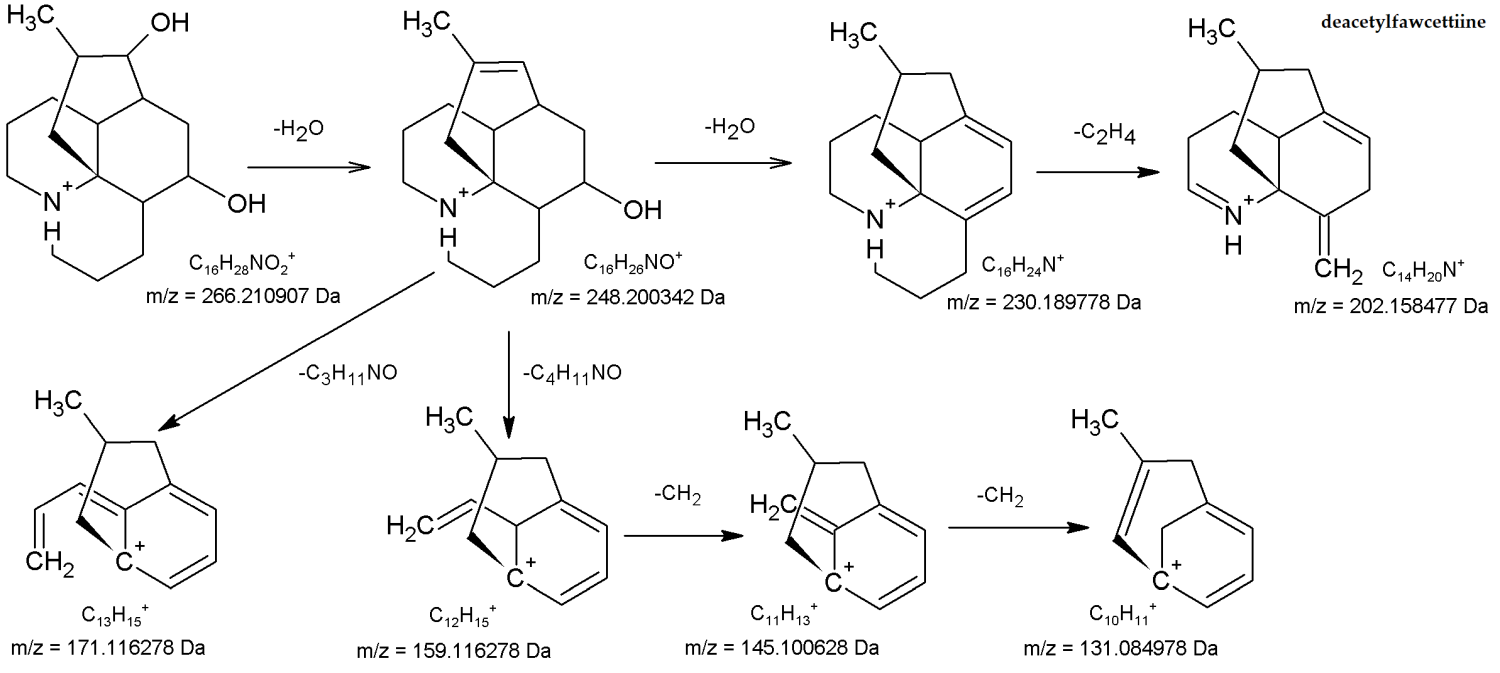

Supplement: Supplementary file 1 [file molecules-26-06379-s001.zip › Fig.S18.b.tif]

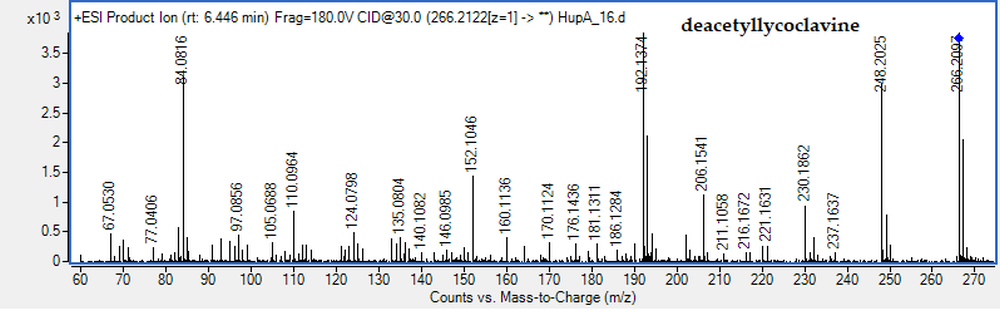

Supplement: Supplementary file 1 [file molecules-26-06379-s001.zip › Fig.S19.a.tif]

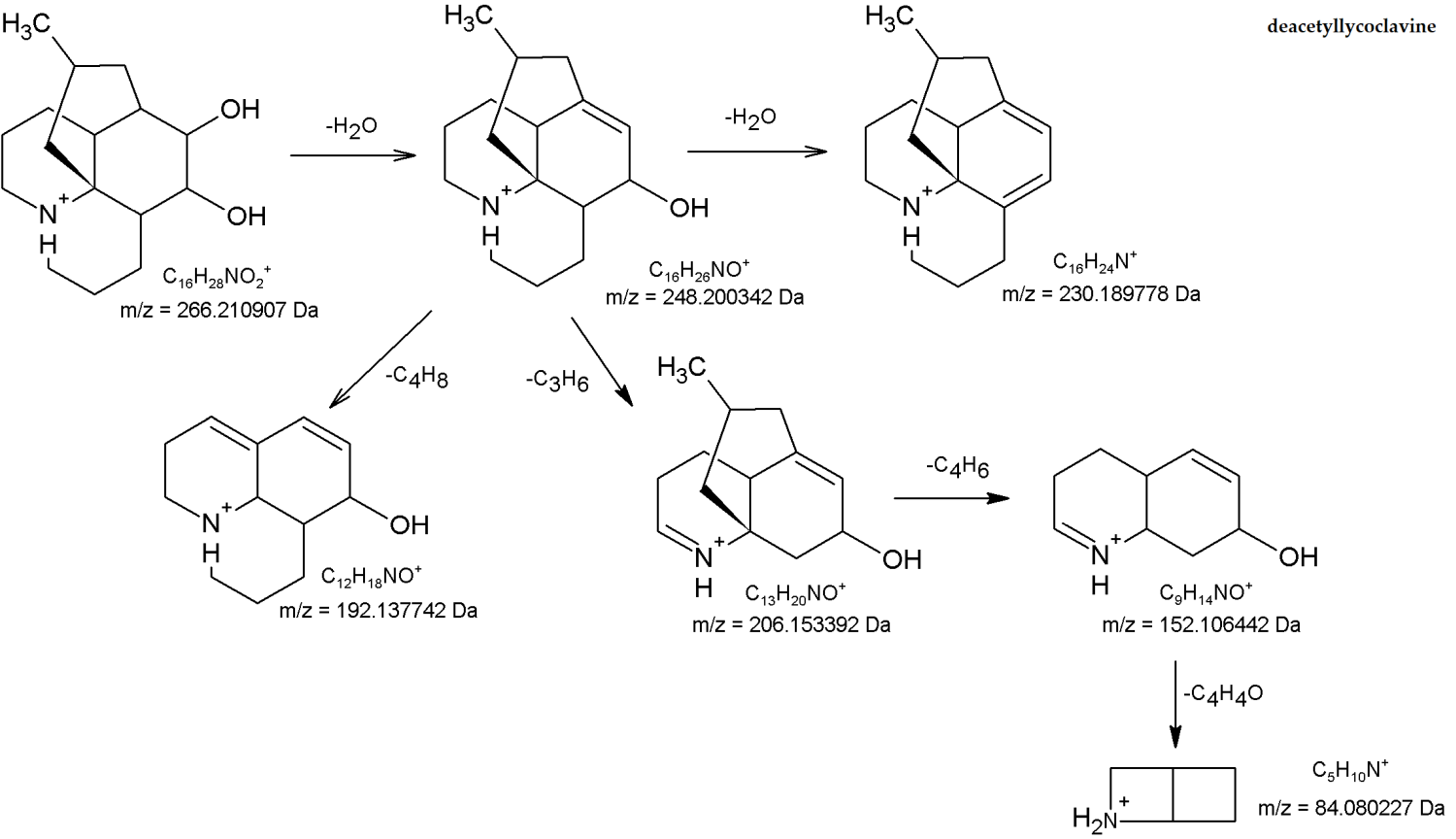

Supplement: Supplementary file 1 [file molecules-26-06379-s001.zip › Fig.S19.b.tif]

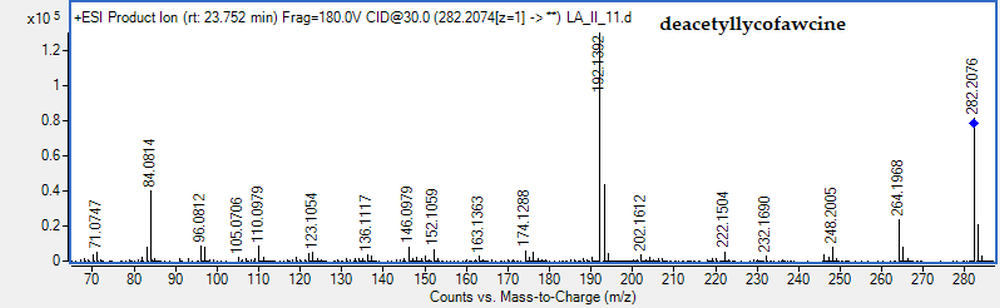

Supplement: Supplementary file 1 [file molecules-26-06379-s001.zip › Fig.S20.a.tif]

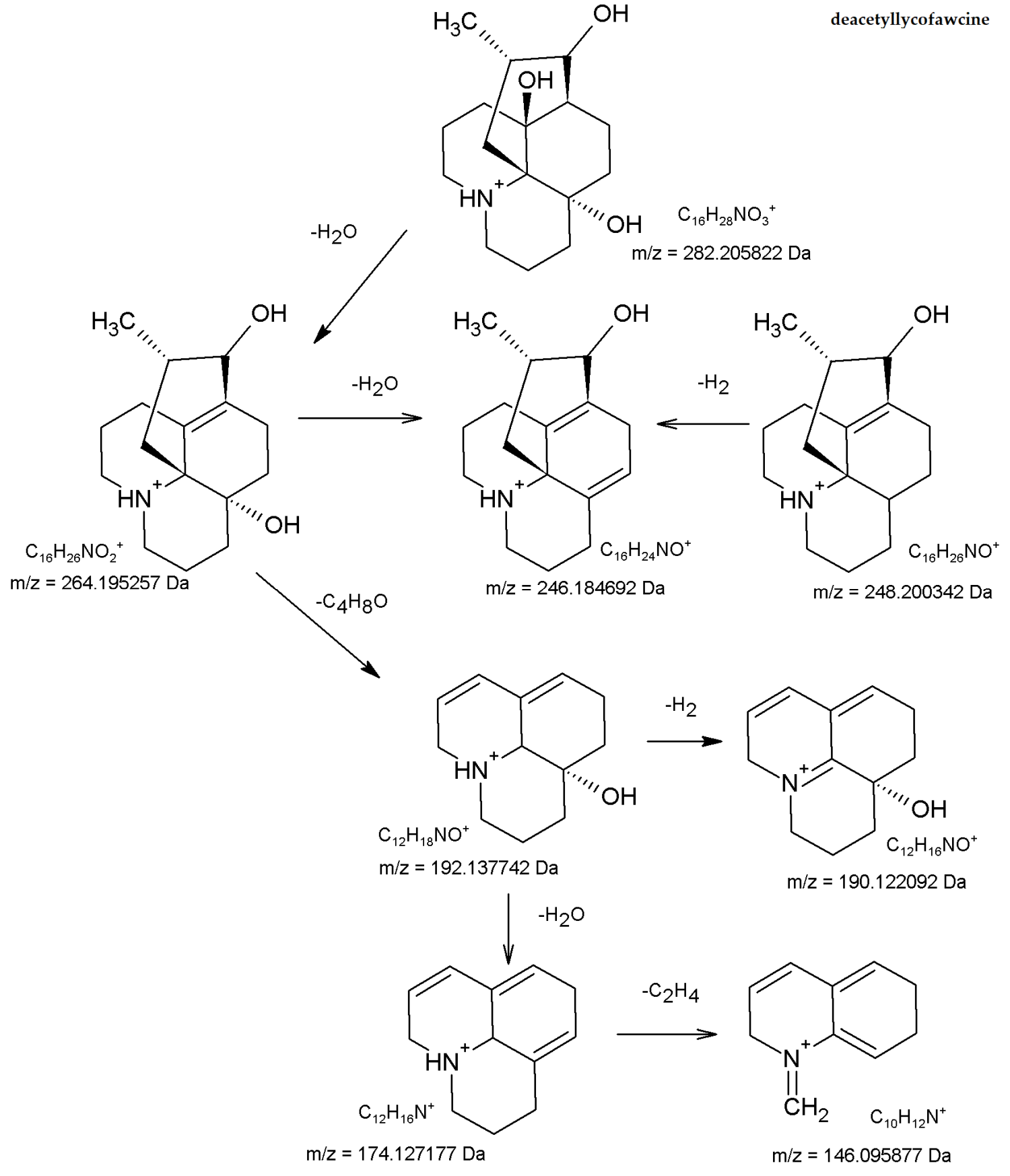

Supplement: Supplementary file 1 [file molecules-26-06379-s001.zip › Fig.S20.b.tif]

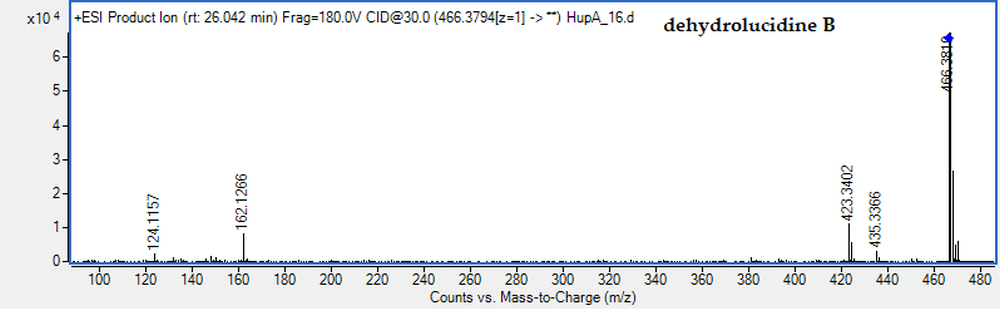

Supplement: Supplementary file 1 [file molecules-26-06379-s001.zip › Fig.S21.a.tif]

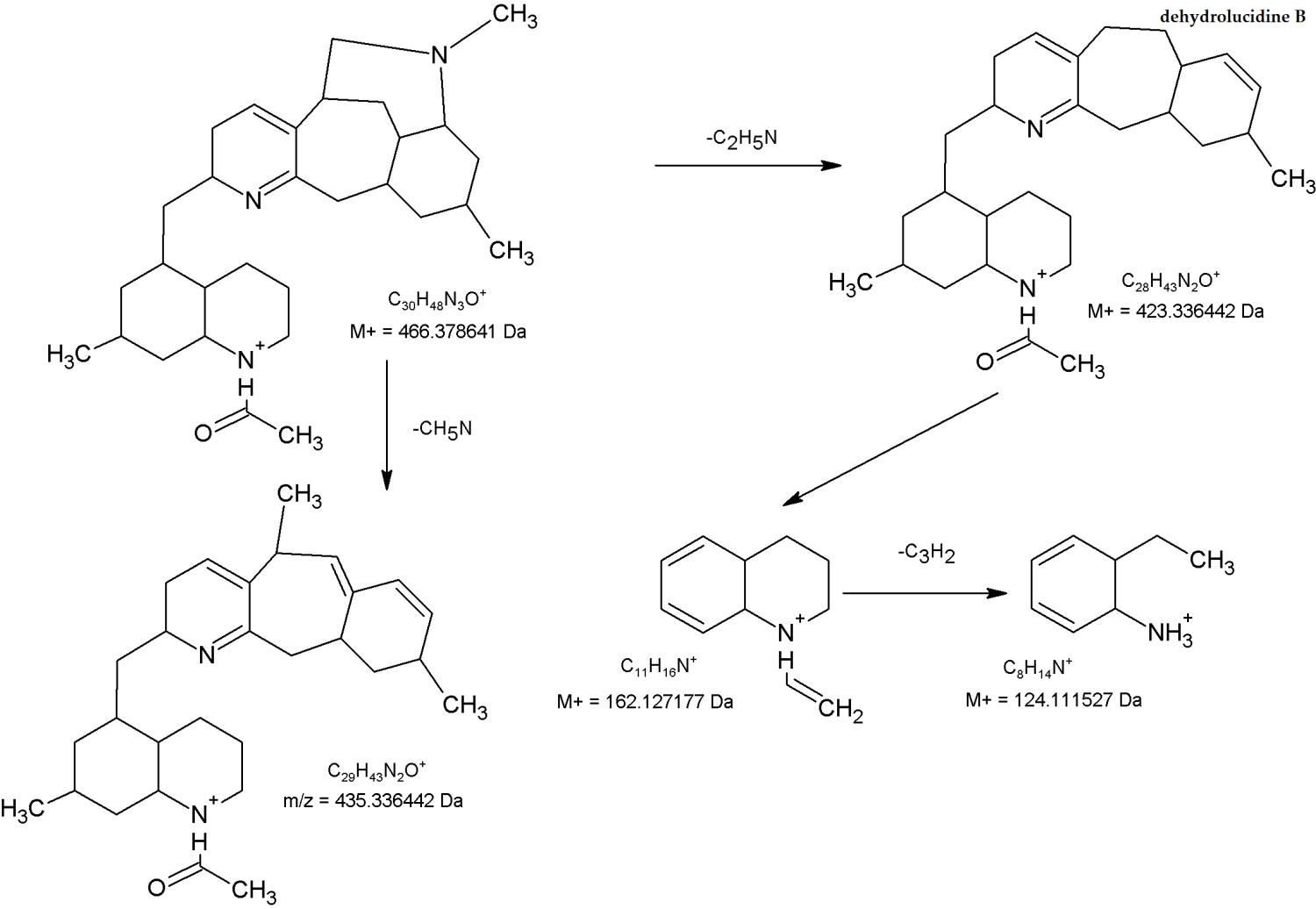

Supplement: Supplementary file 1 [file molecules-26-06379-s001.zip › Fig.S21.b.tif]

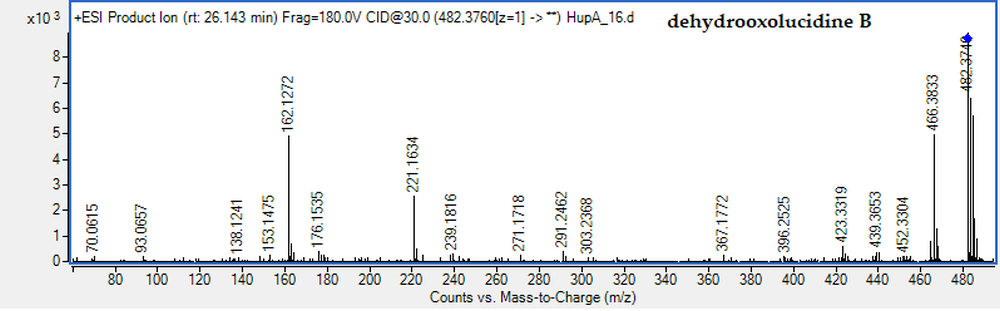

Supplement: Supplementary file 1 [file molecules-26-06379-s001.zip › Fig.S22.a.tif]

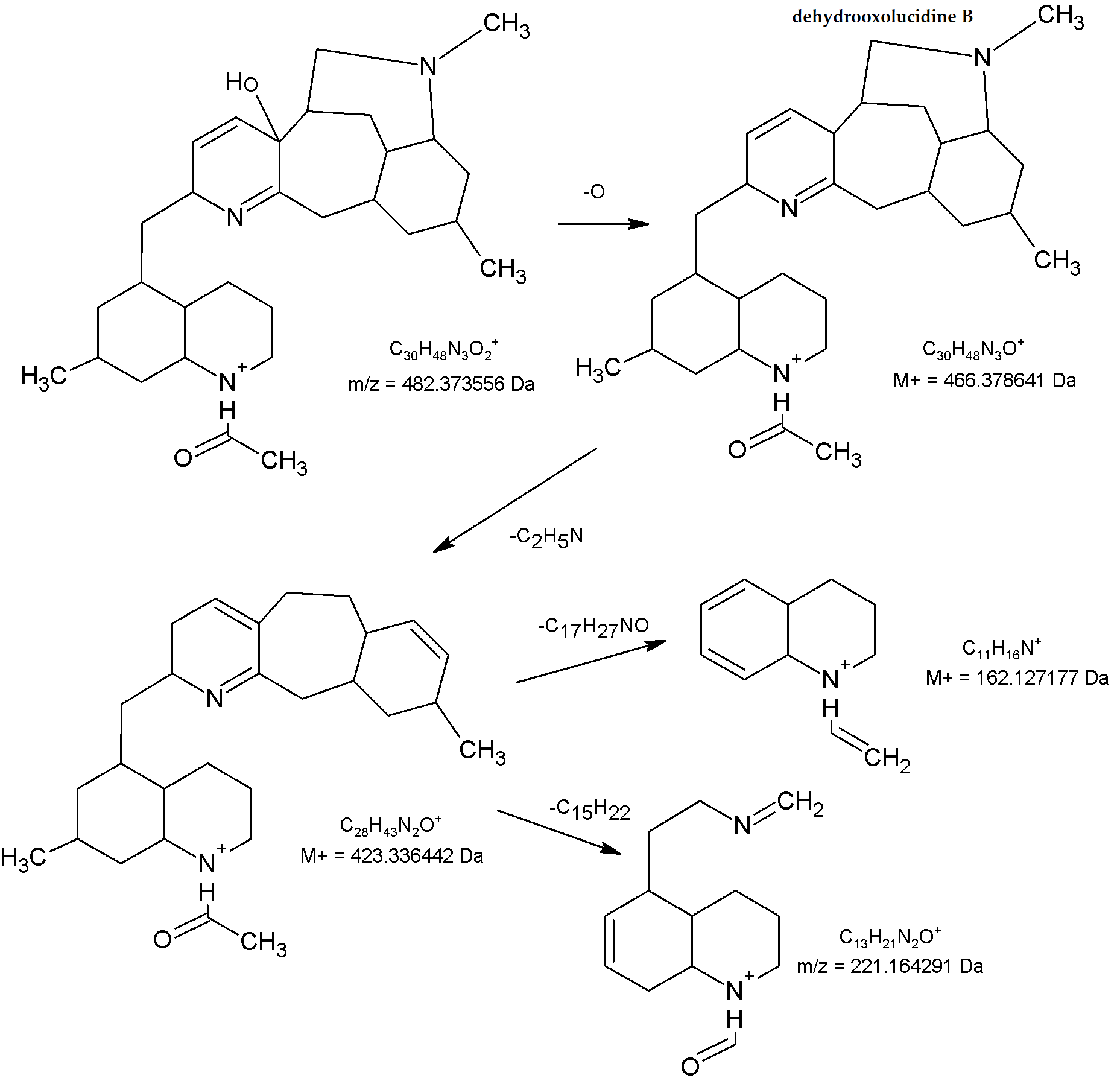

Supplement: Supplementary file 1 [file molecules-26-06379-s001.zip › Fig.S22.b.tif]

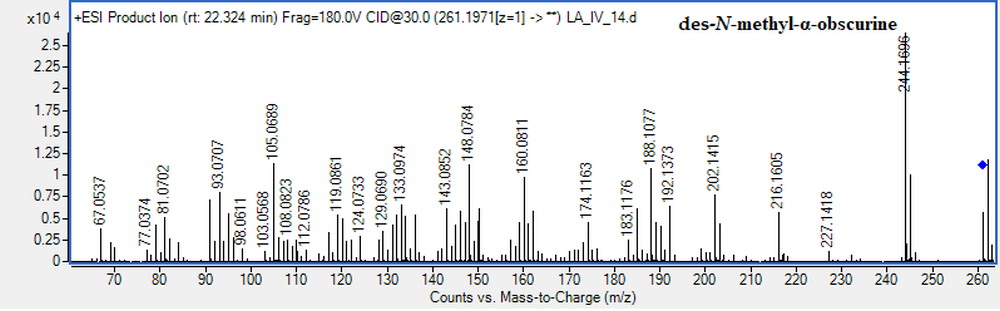

Supplement: Supplementary file 1 [file molecules-26-06379-s001.zip › Fig.S23.a.tif]

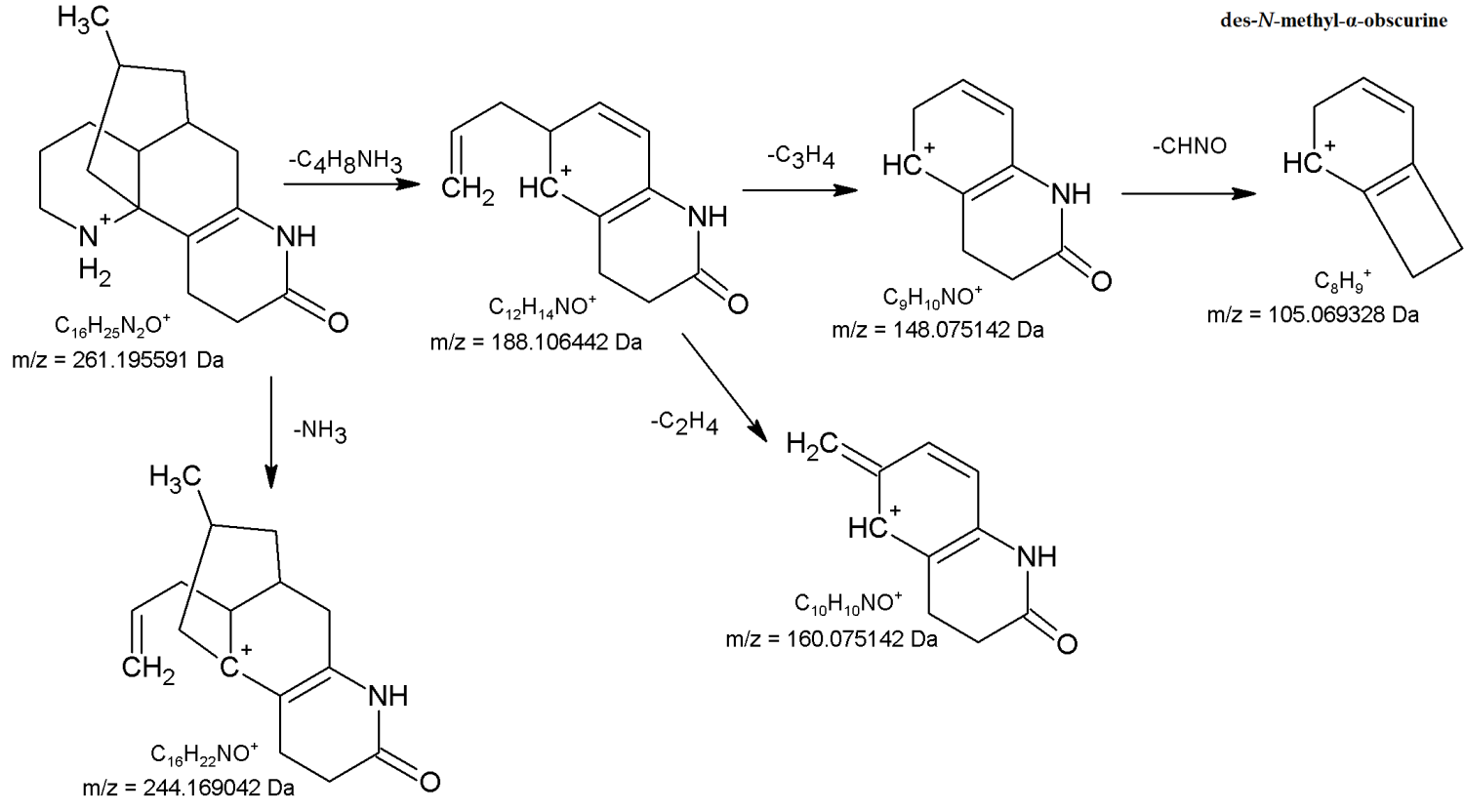

Supplement: Supplementary file 1 [file molecules-26-06379-s001.zip › Fig.S23.b.tif]

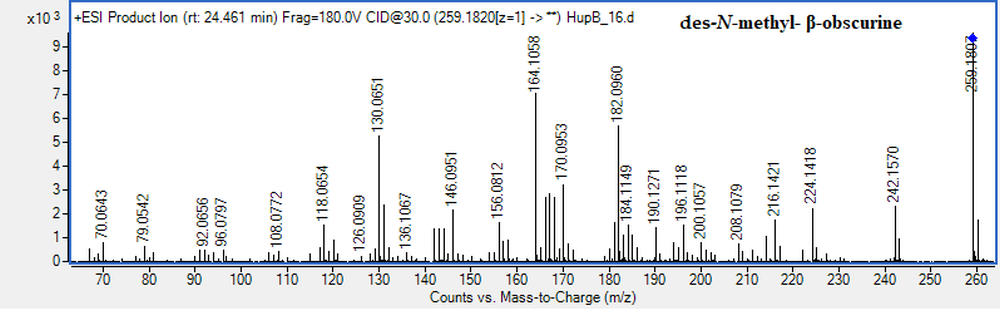

Supplement: Supplementary file 1 [file molecules-26-06379-s001.zip › Fig.S24.a.tif]

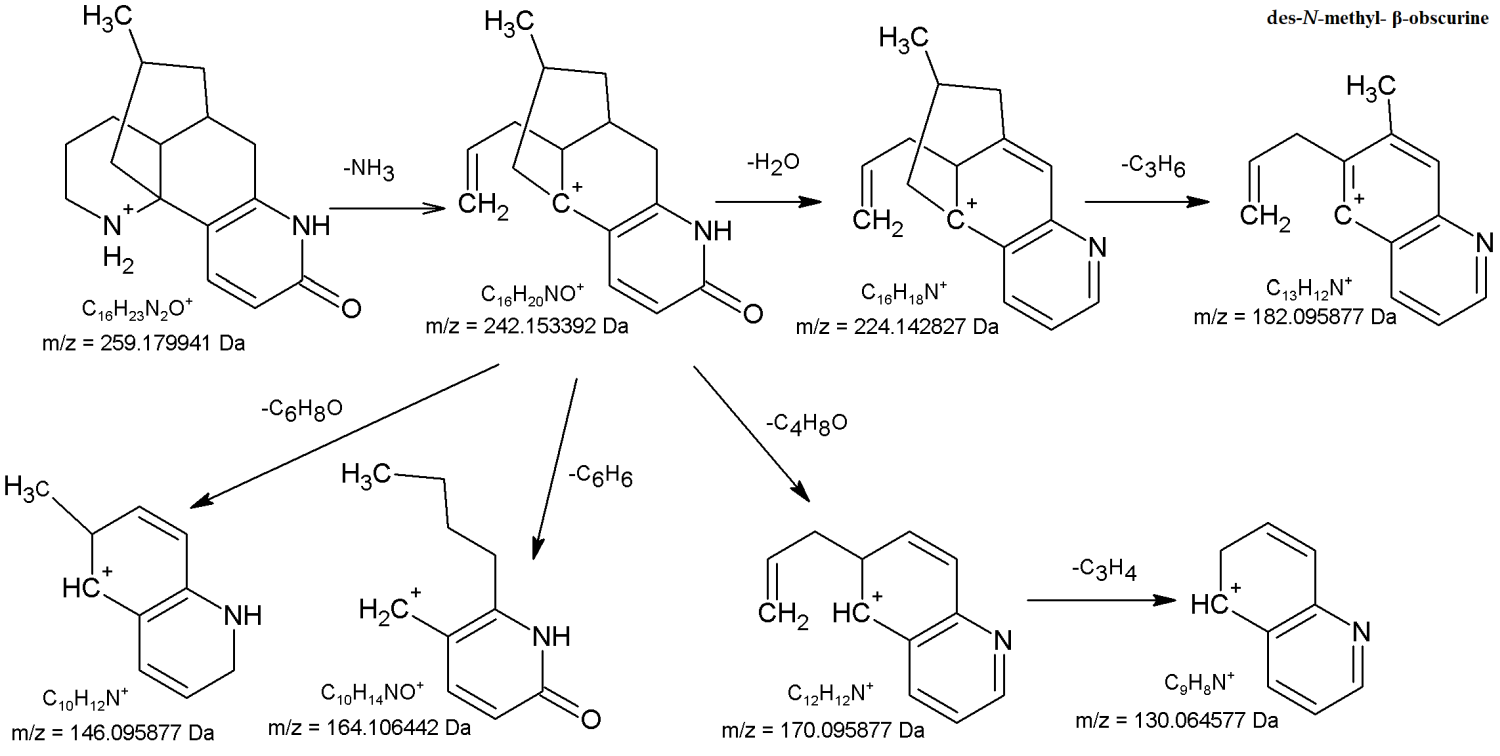

Supplement: Supplementary file 1 [file molecules-26-06379-s001.zip › Fig.S24.b.tif]

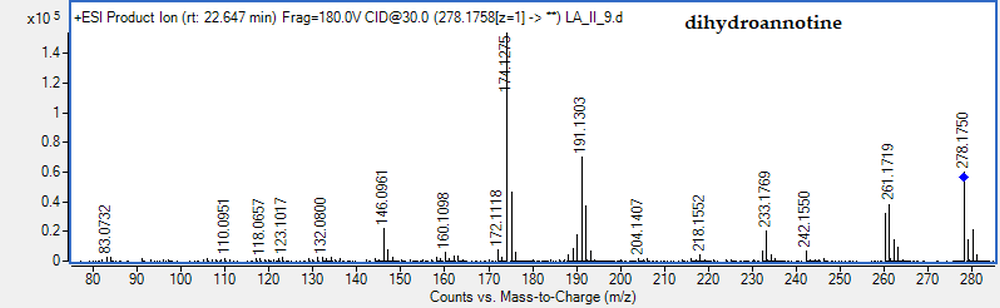

Supplement: Supplementary file 1 [file molecules-26-06379-s001.zip › Fig.S25.a.tif]

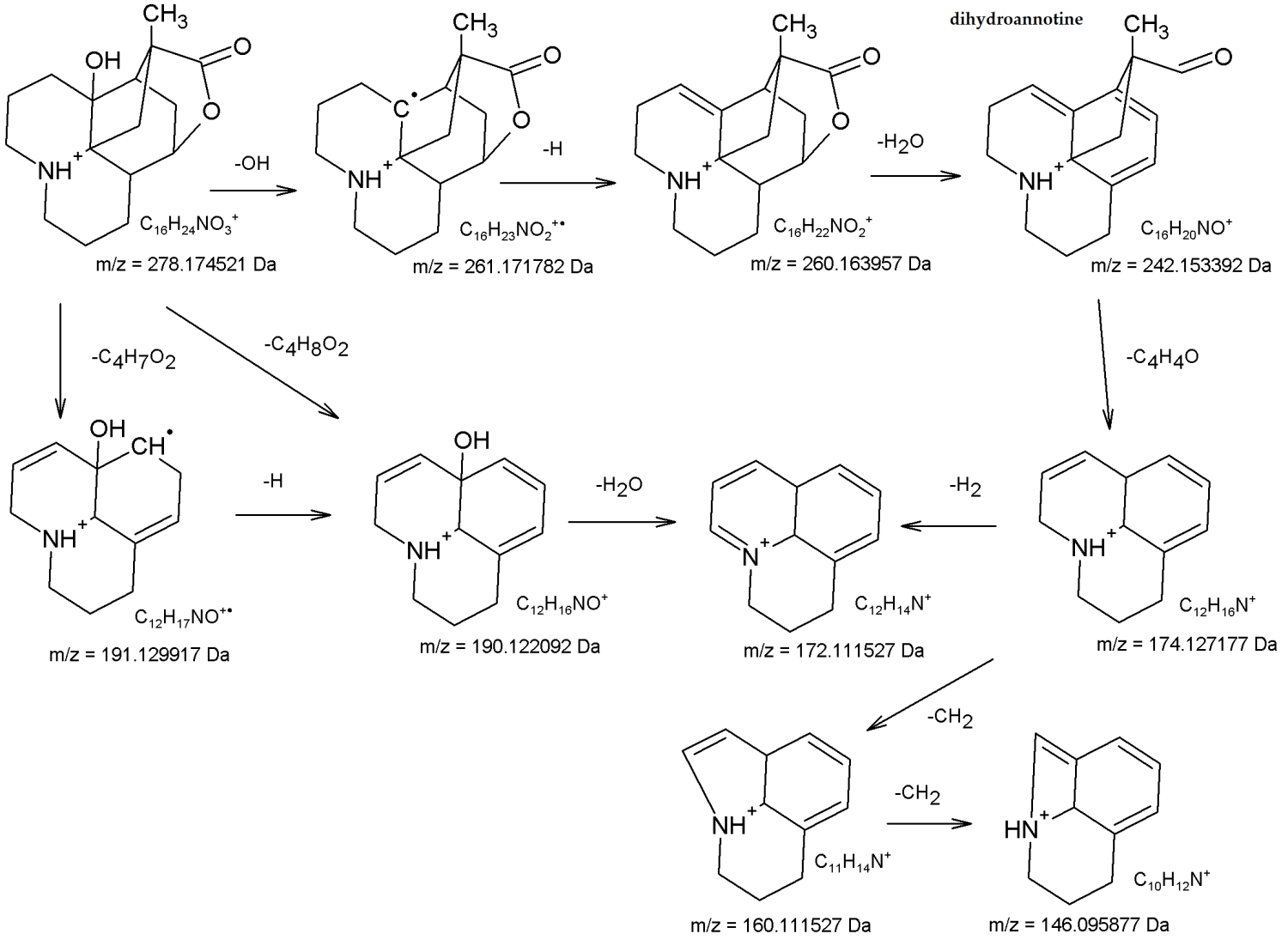

Supplement: Supplementary file 1 [file molecules-26-06379-s001.zip › Fig.S25.b.tif]

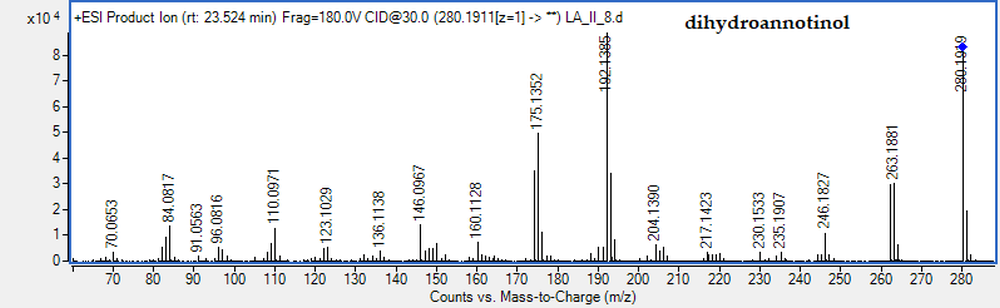

Supplement: Supplementary file 1 [file molecules-26-06379-s001.zip › Fig.S26.a.tif]

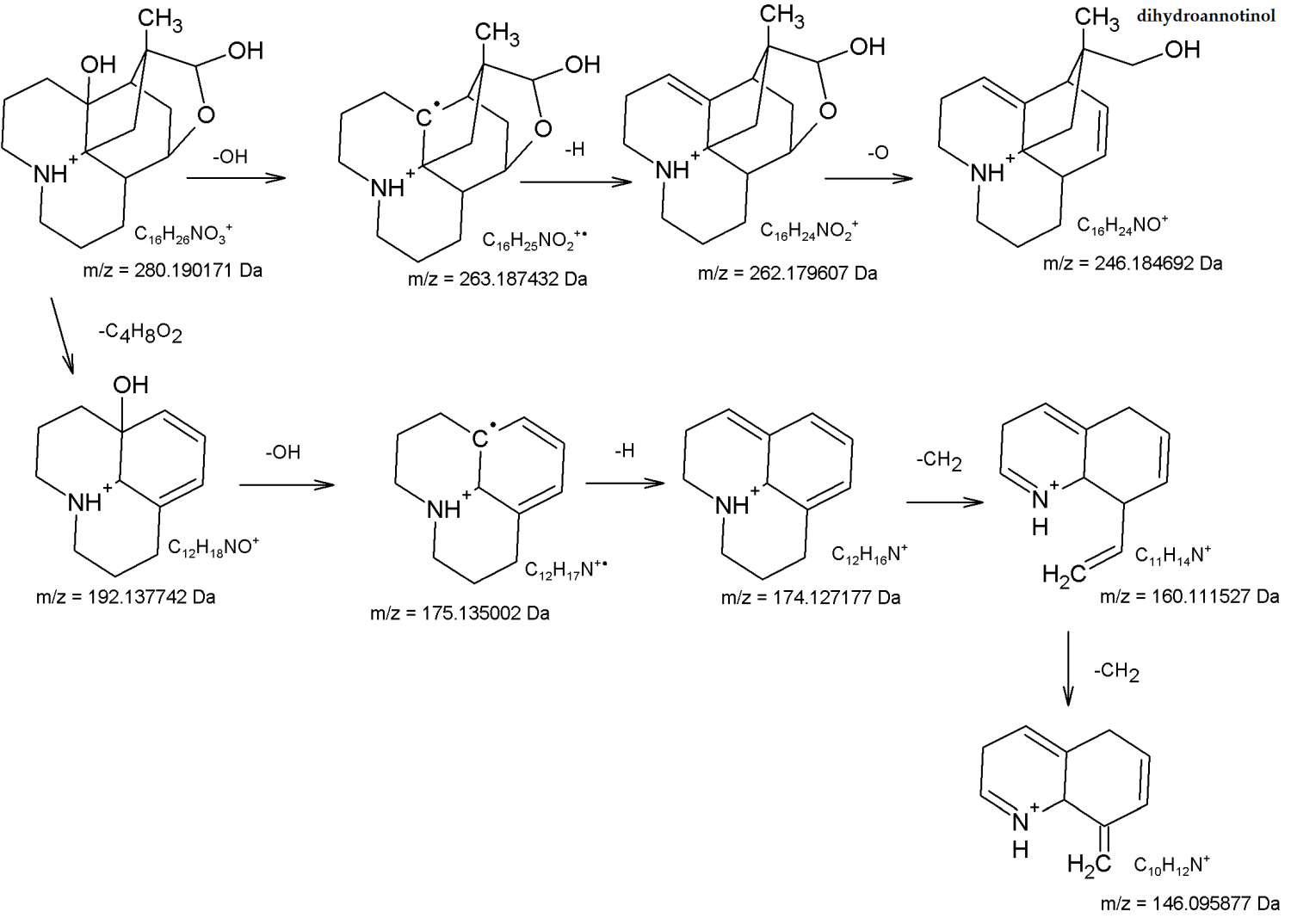

Supplement: Supplementary file 1 [file molecules-26-06379-s001.zip › Fig.S26.b.tif]

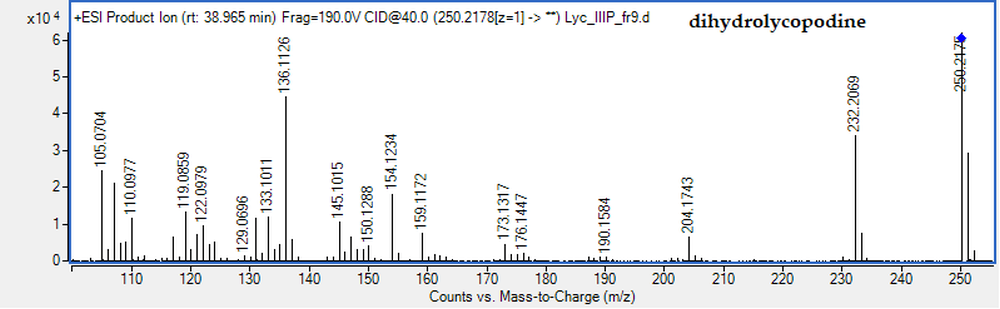

Supplement: Supplementary file 1 [file molecules-26-06379-s001.zip › Fig.S27.a.tif]

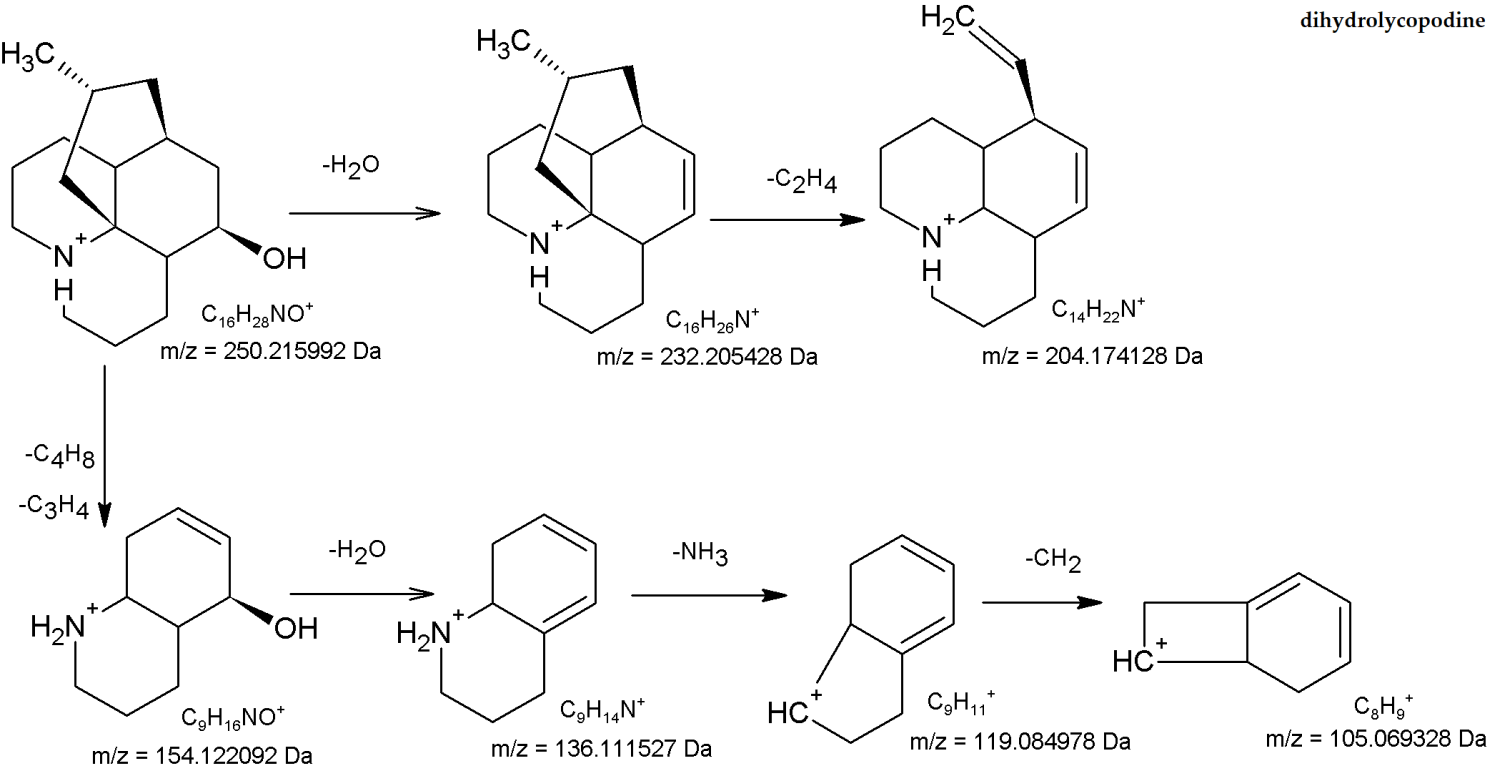

Supplement: Supplementary file 1 [file molecules-26-06379-s001.zip › Fig.S27.b.tif]

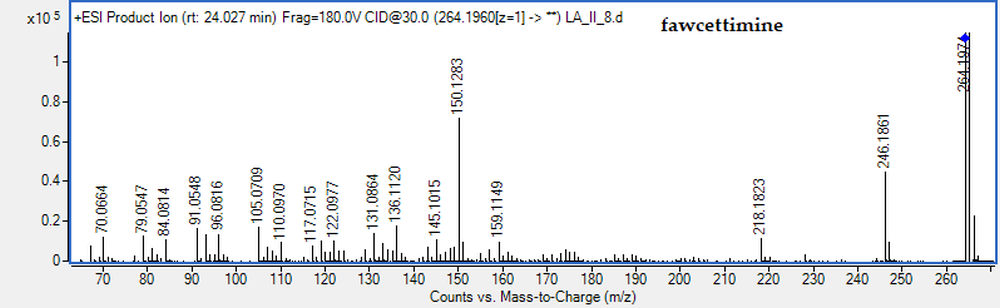

Supplement: Supplementary file 1 [file molecules-26-06379-s001.zip › Fig.S28.a.tif]

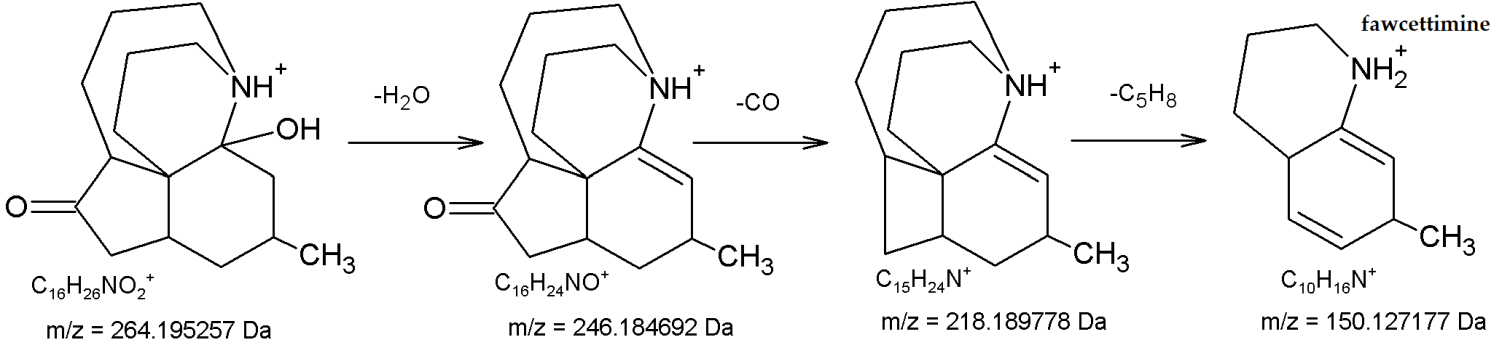

Supplement: Supplementary file 1 [file molecules-26-06379-s001.zip › Fig.S28.b.tif]

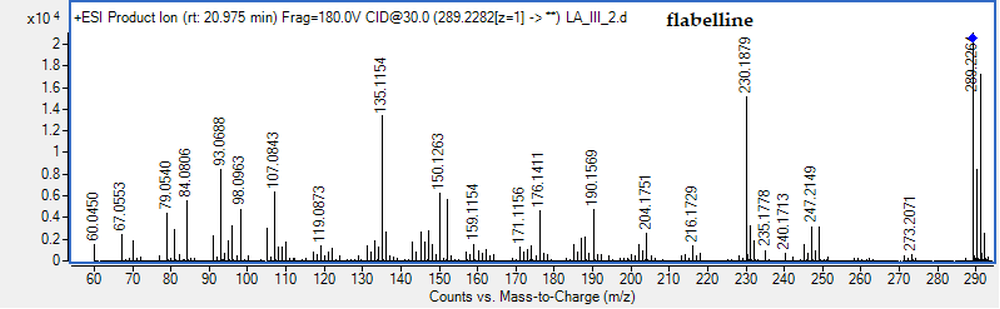

Supplement: Supplementary file 1 [file molecules-26-06379-s001.zip › Fig.S29.a.tif]

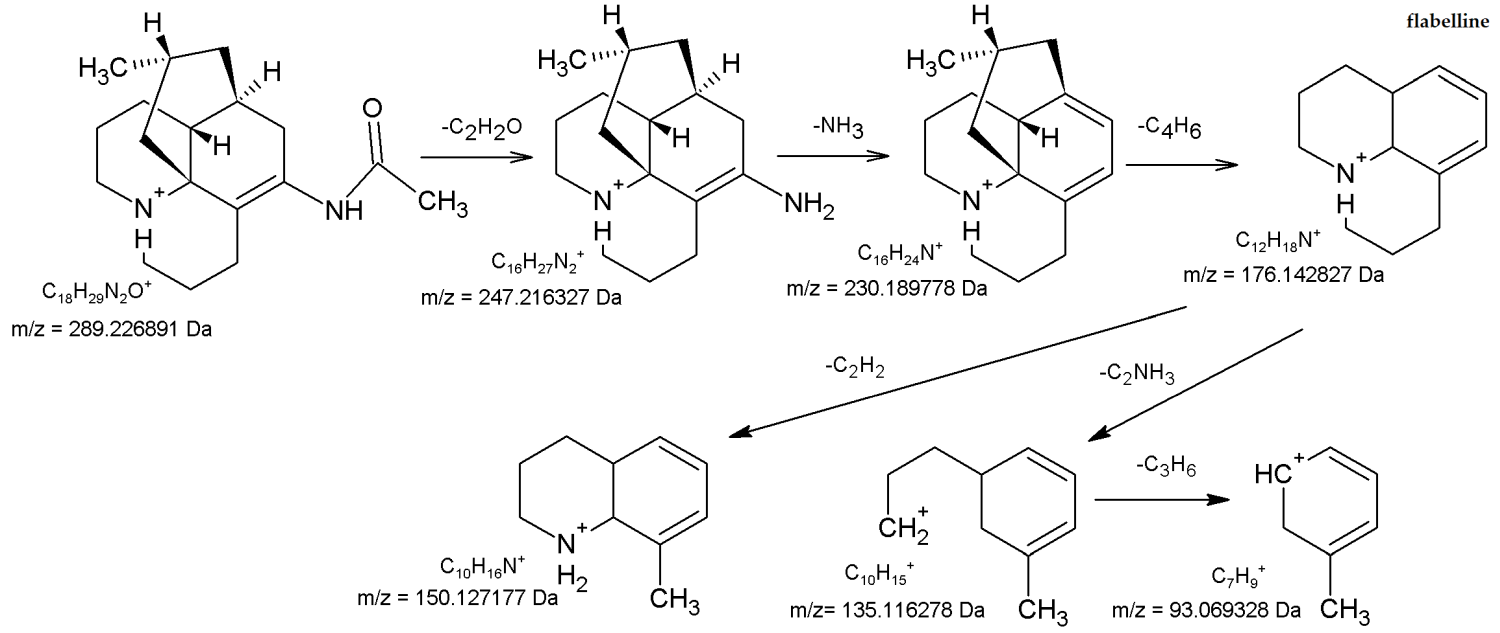

Supplement: Supplementary file 1 [file molecules-26-06379-s001.zip › Fig.S29.b.tif]

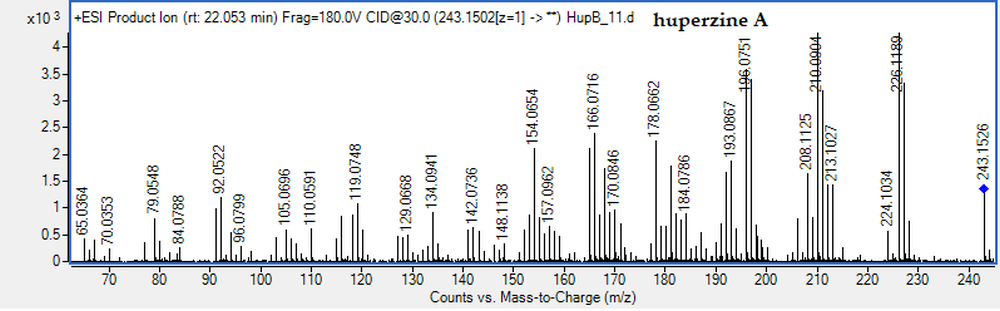

Supplement: Supplementary file 1 [file molecules-26-06379-s001.zip › Fig.S30.a.tif]

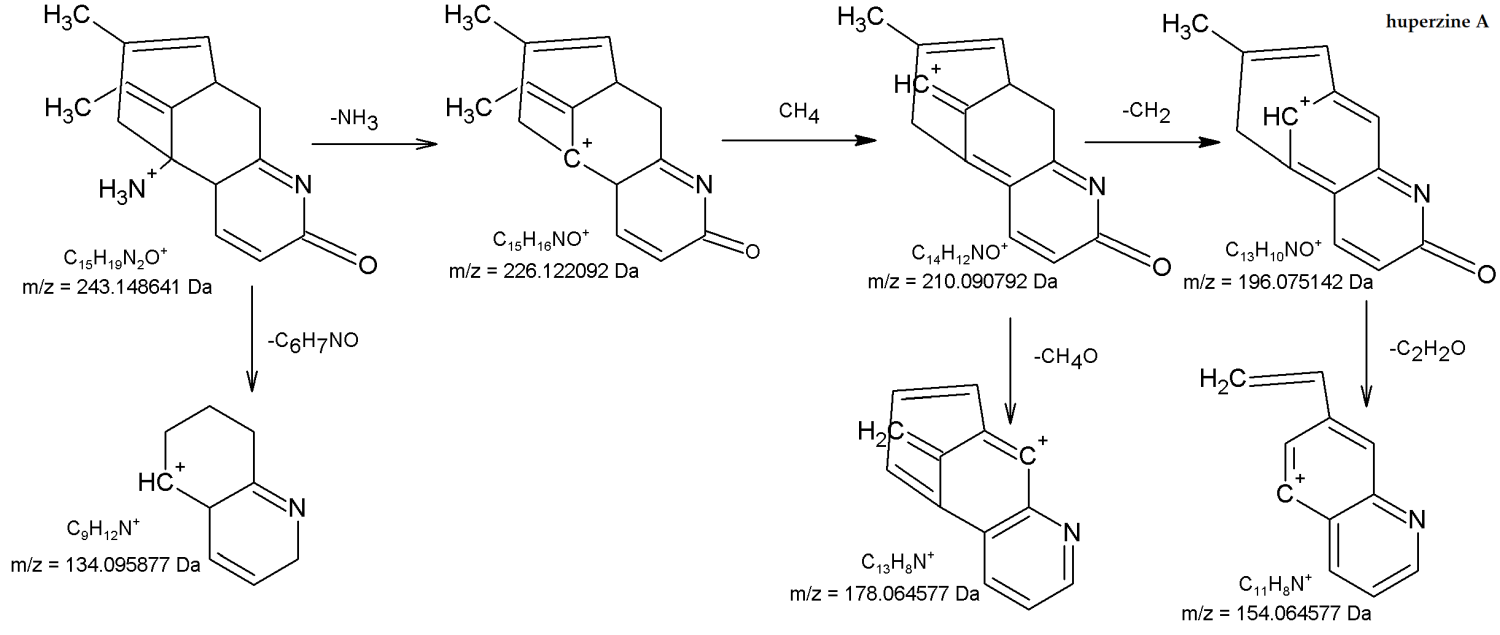

Supplement: Supplementary file 1 [file molecules-26-06379-s001.zip › Fig.S30.b.tif]

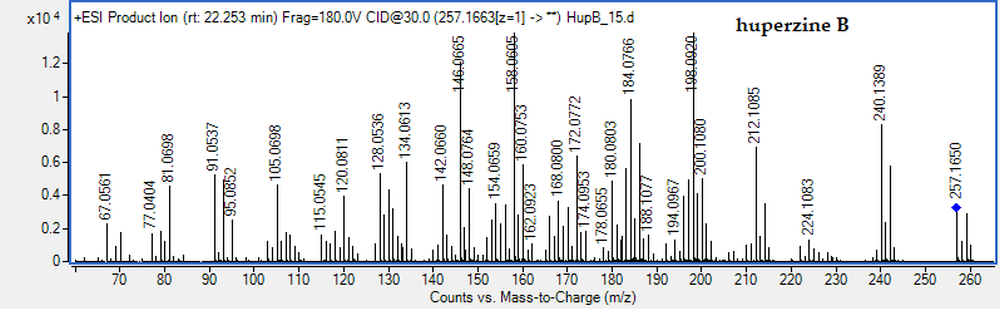

Supplement: Supplementary file 1 [file molecules-26-06379-s001.zip › Fig.S31.a.tif]

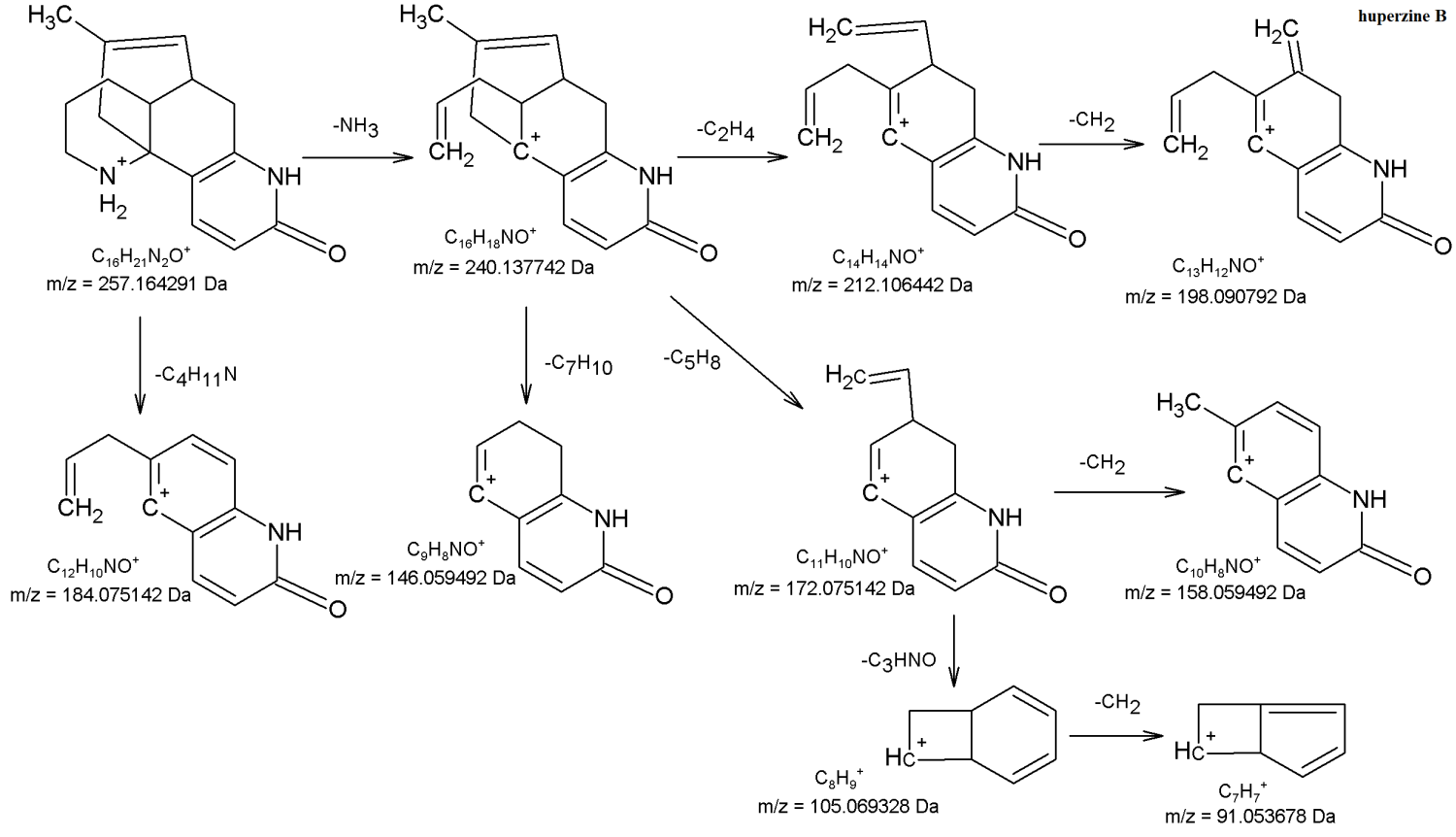

Supplement: Supplementary file 1 [file molecules-26-06379-s001.zip › Fig.S31.b.tif]

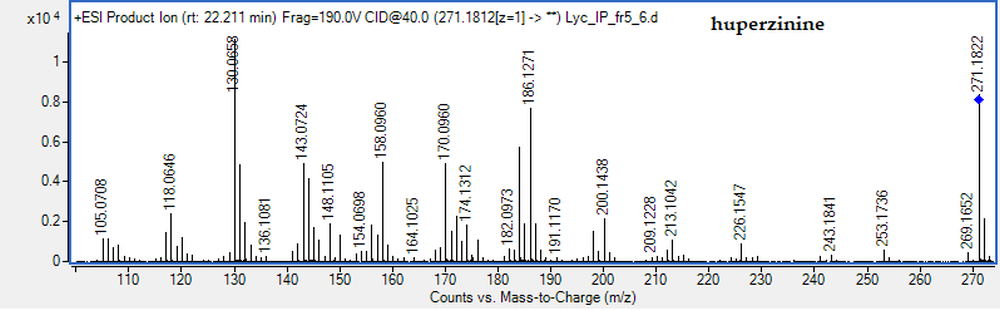

Supplement: Supplementary file 1 [file molecules-26-06379-s001.zip › Fig.S32.a.tif]

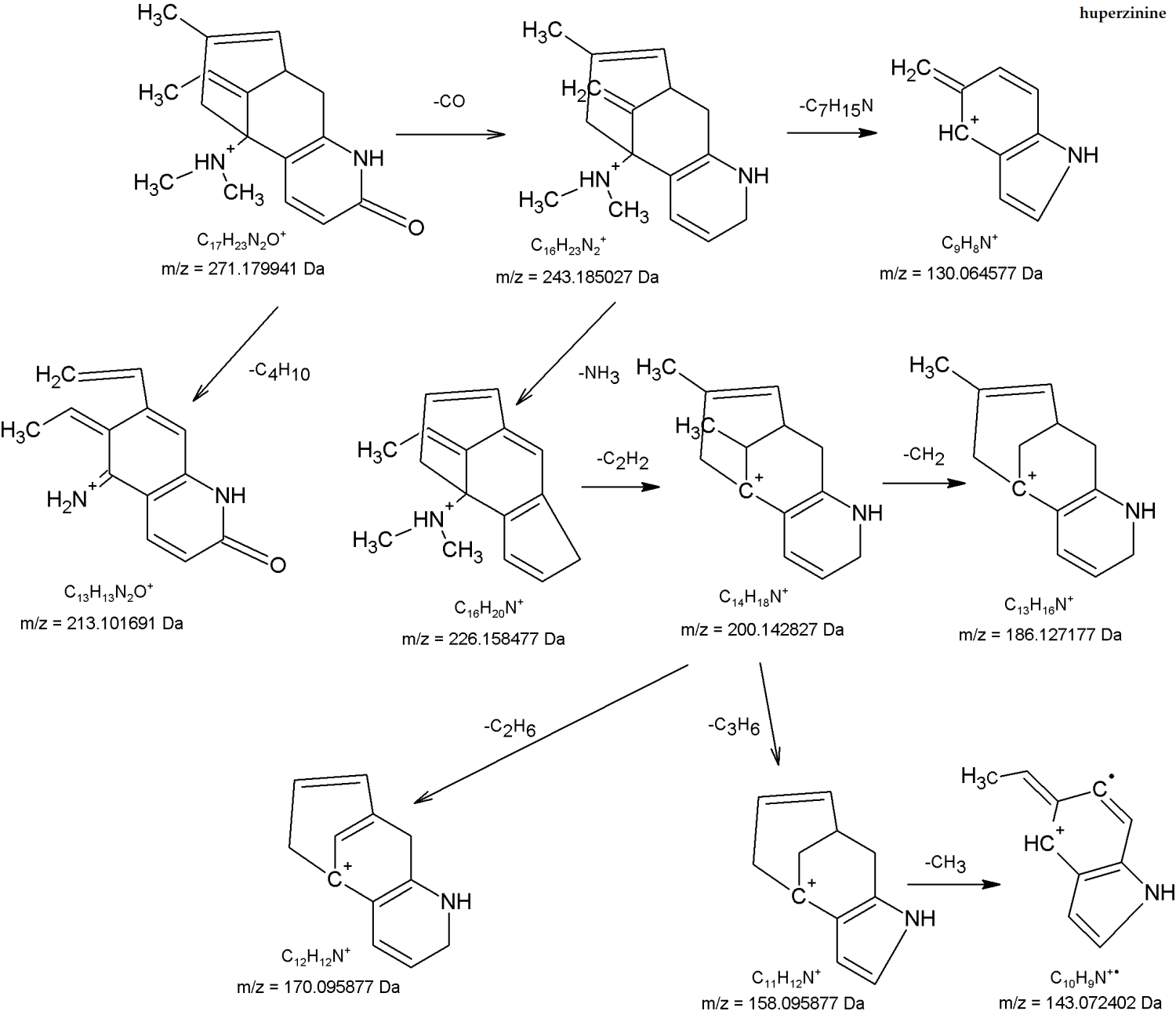

Supplement: Supplementary file 1 [file molecules-26-06379-s001.zip › Fig.S32.b.tif]

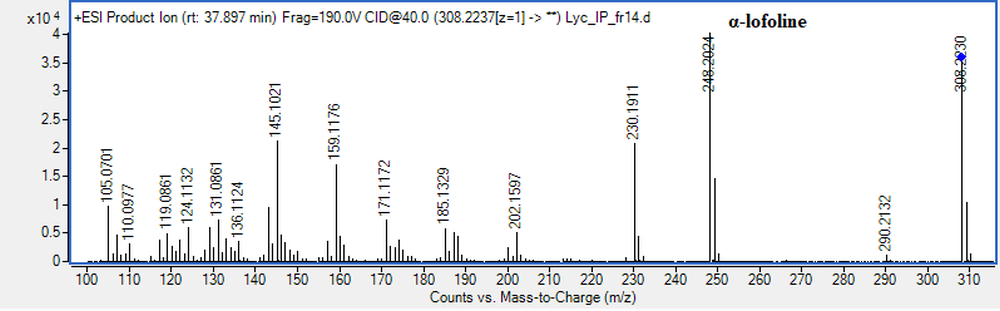

Supplement: Supplementary file 1 [file molecules-26-06379-s001.zip › Fig.S33.a.tif]

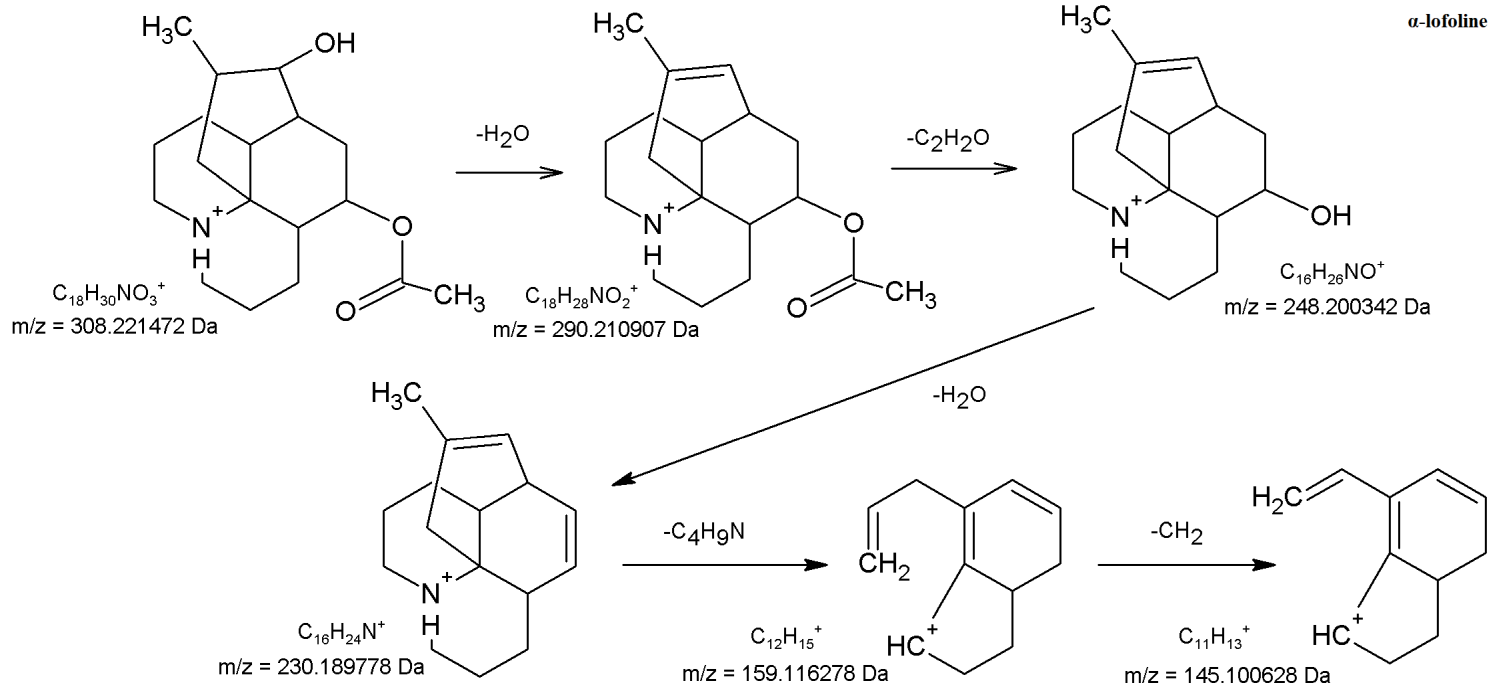

Supplement: Supplementary file 1 [file molecules-26-06379-s001.zip › Fig.S33.b.tif]

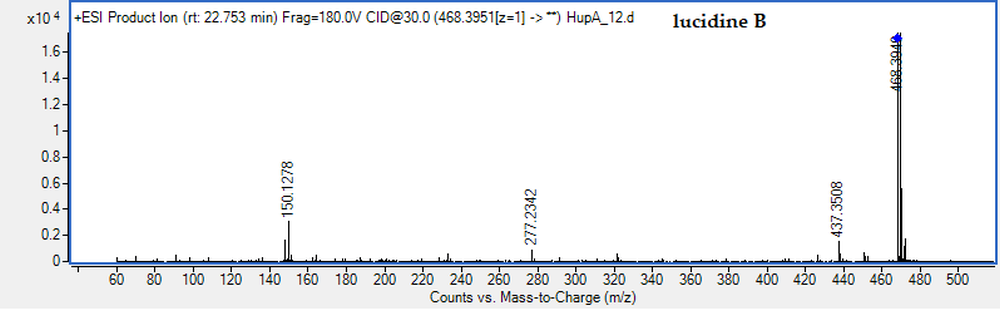

Supplement: Supplementary file 1 [file molecules-26-06379-s001.zip › Fig.S34.a.tif]

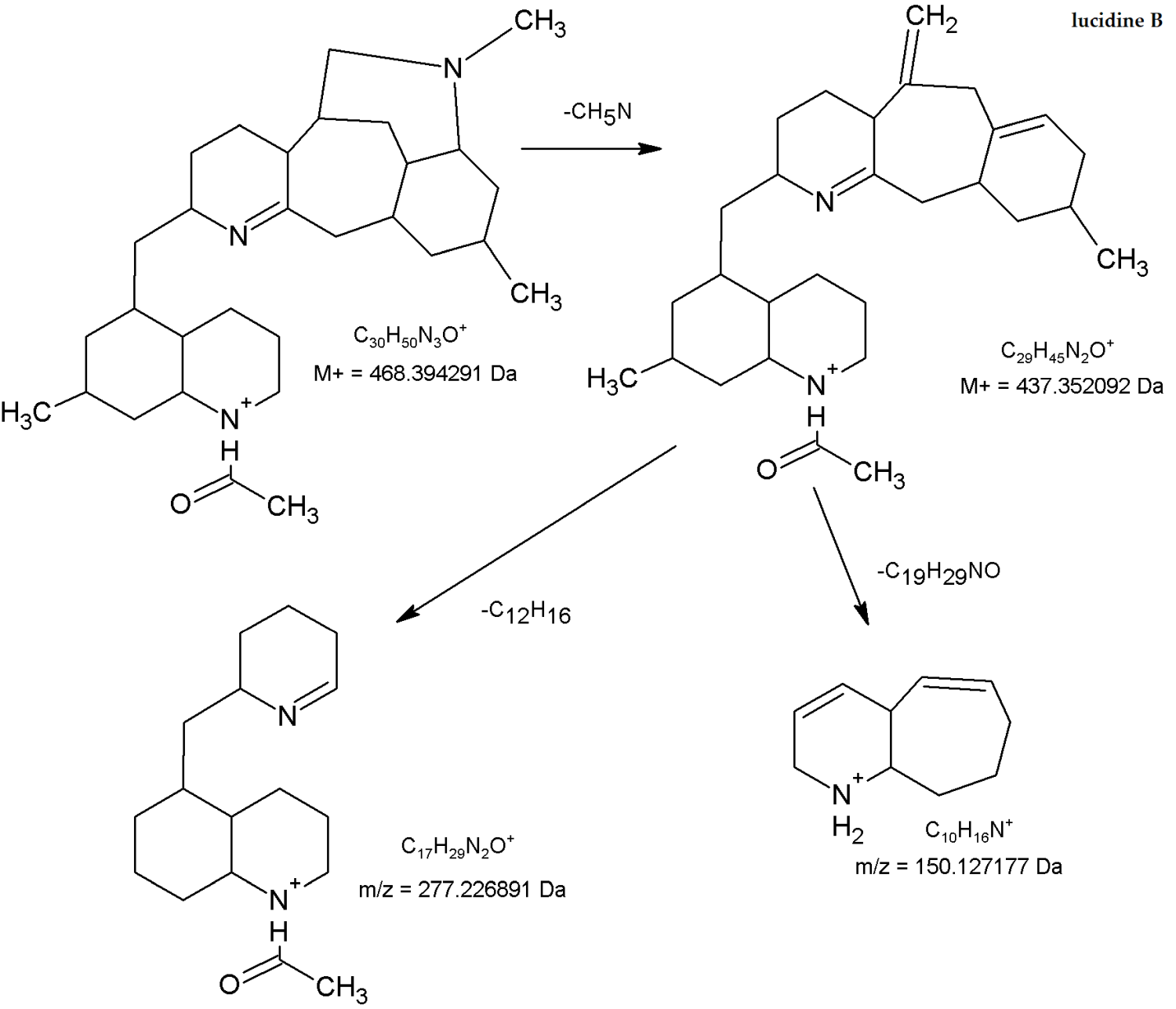

Supplement: Supplementary file 1 [file molecules-26-06379-s001.zip › Fig.S34.b.tif]

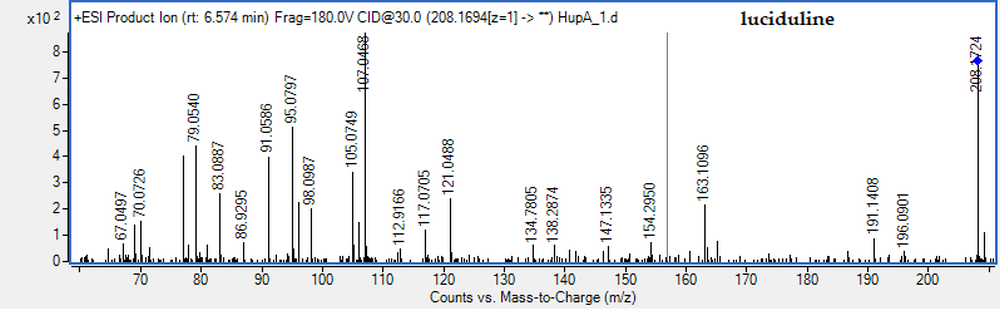

Supplement: Supplementary file 1 [file molecules-26-06379-s001.zip › Fig.S35.a.tif]

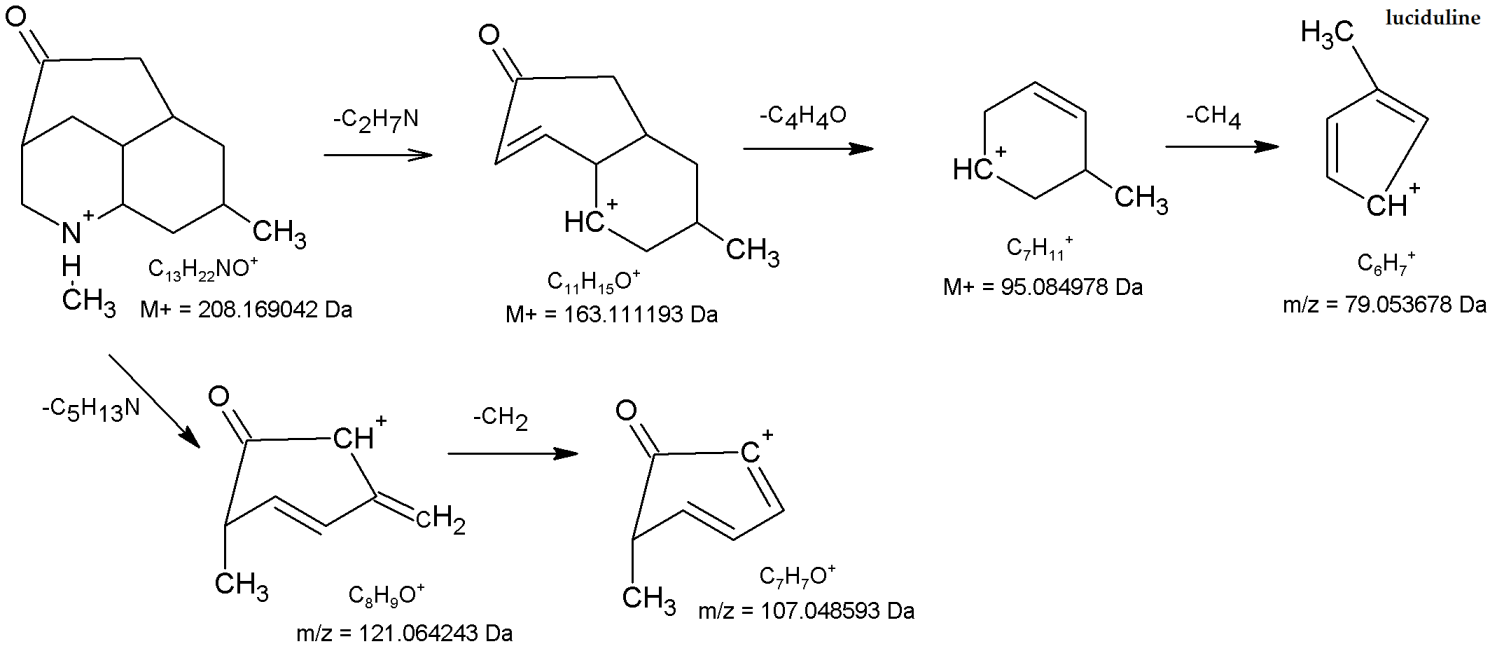

Supplement: Supplementary file 1 [file molecules-26-06379-s001.zip › Fig.S35.b.tif]

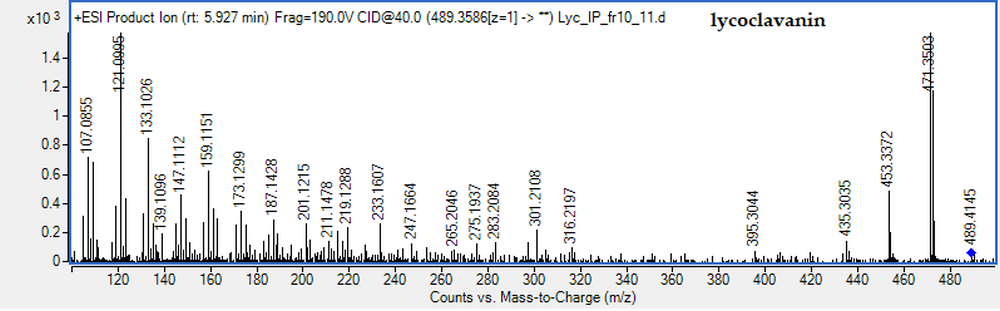

Supplement: Supplementary file 1 [file molecules-26-06379-s001.zip › Fig.S36.a.tif]

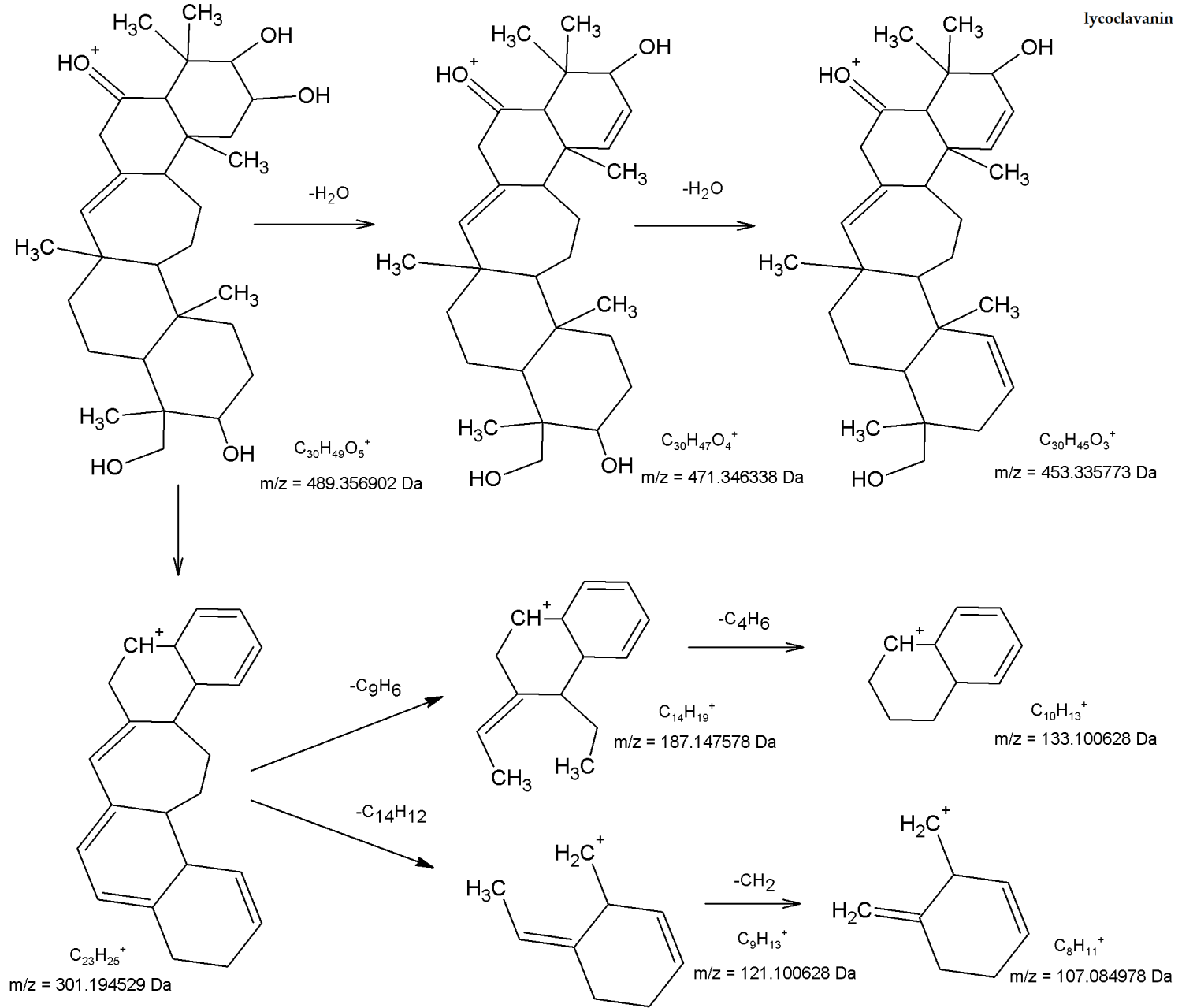

Supplement: Supplementary file 1 [file molecules-26-06379-s001.zip › Fig.S36.b.tif]

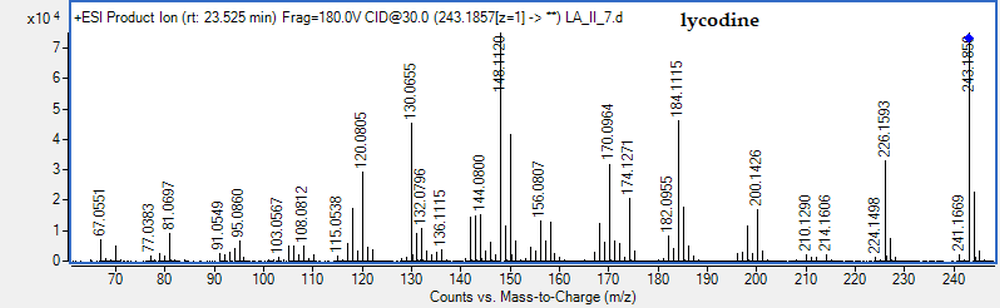

Supplement: Supplementary file 1 [file molecules-26-06379-s001.zip › Fig.S37.a.tif]

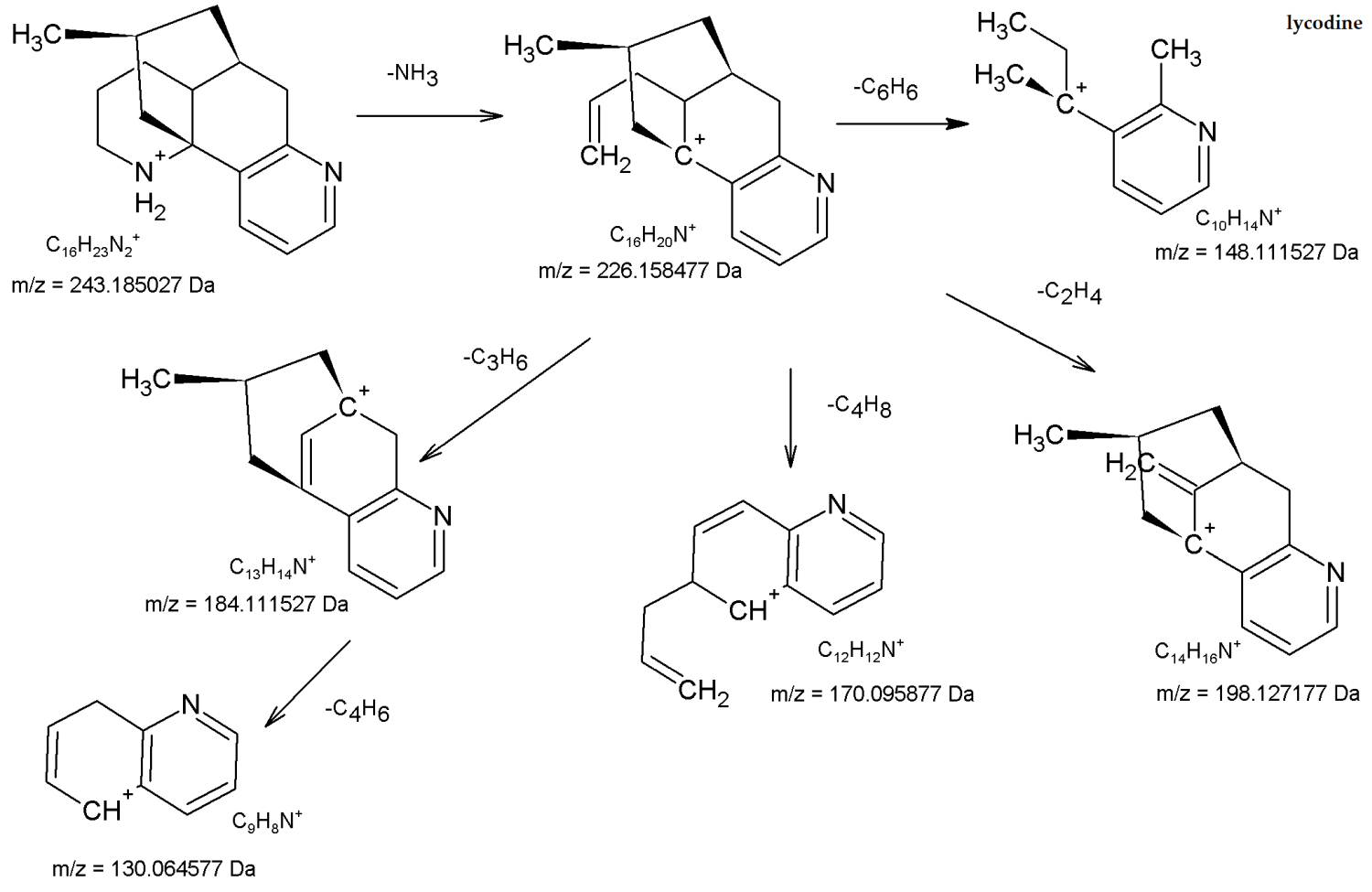

Supplement: Supplementary file 1 [file molecules-26-06379-s001.zip › Fig.S37.b.tif]

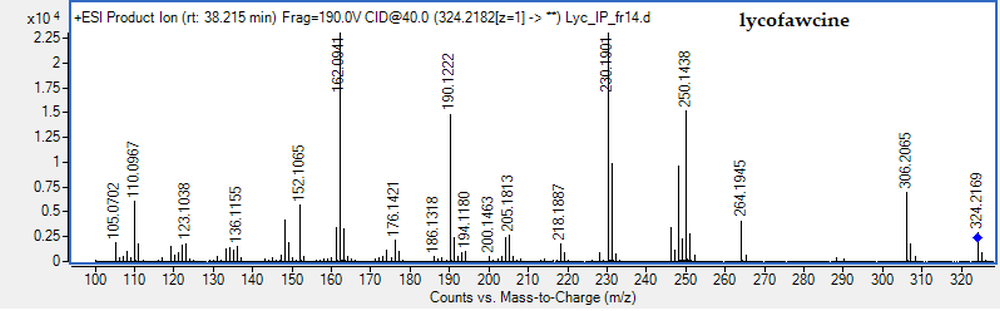

Supplement: Supplementary file 1 [file molecules-26-06379-s001.zip › Fig.S38.a.tif]

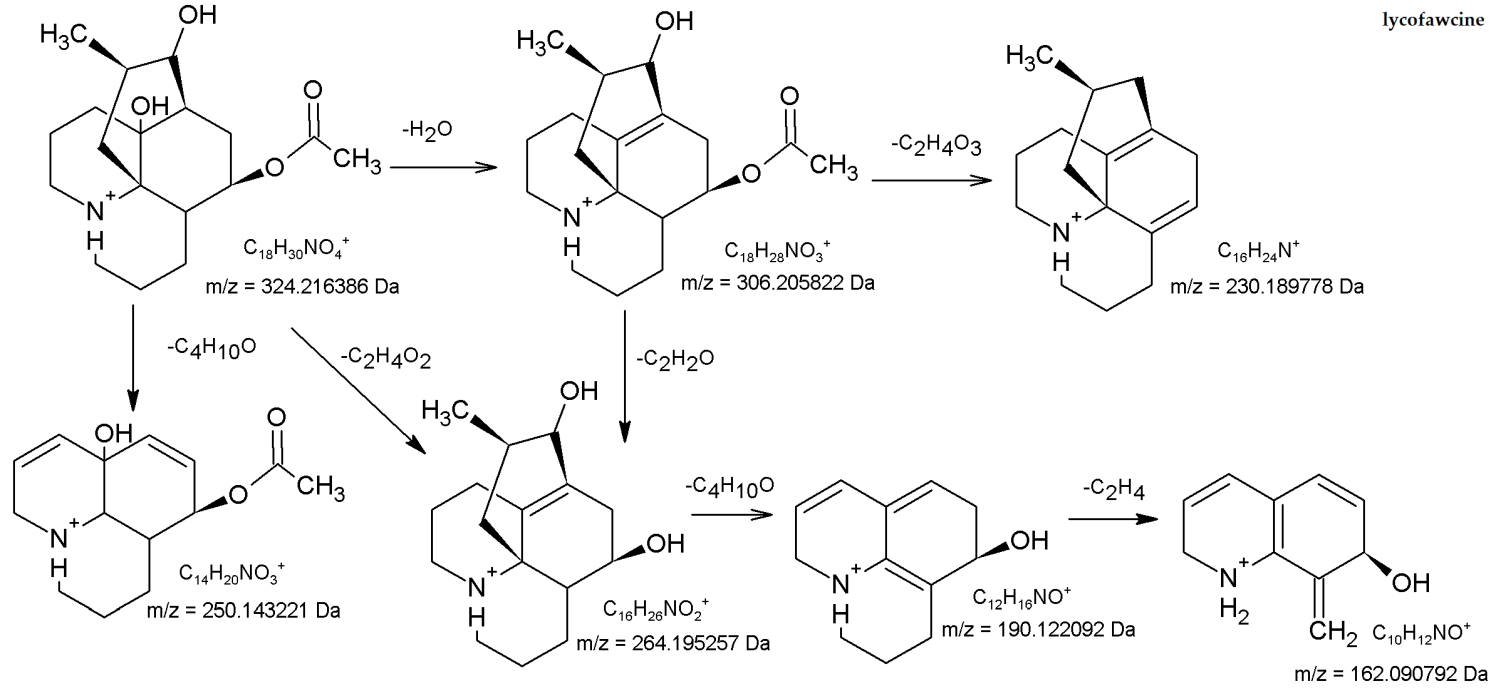

Supplement: Supplementary file 1 [file molecules-26-06379-s001.zip › Fig.S38.b.tif]

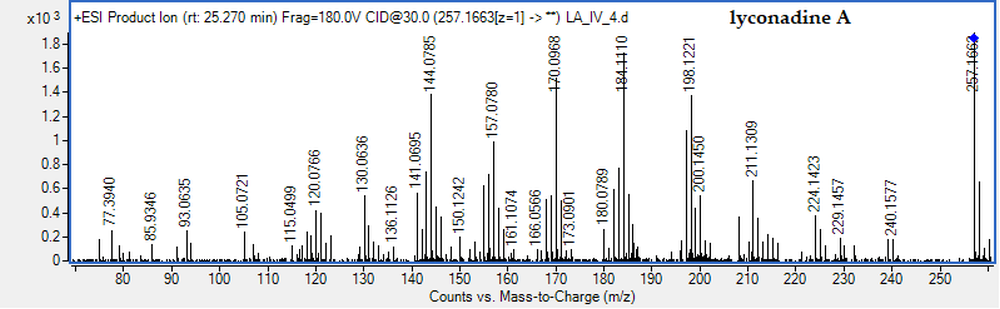

Supplement: Supplementary file 1 [file molecules-26-06379-s001.zip › Fig.S39.a.tif]

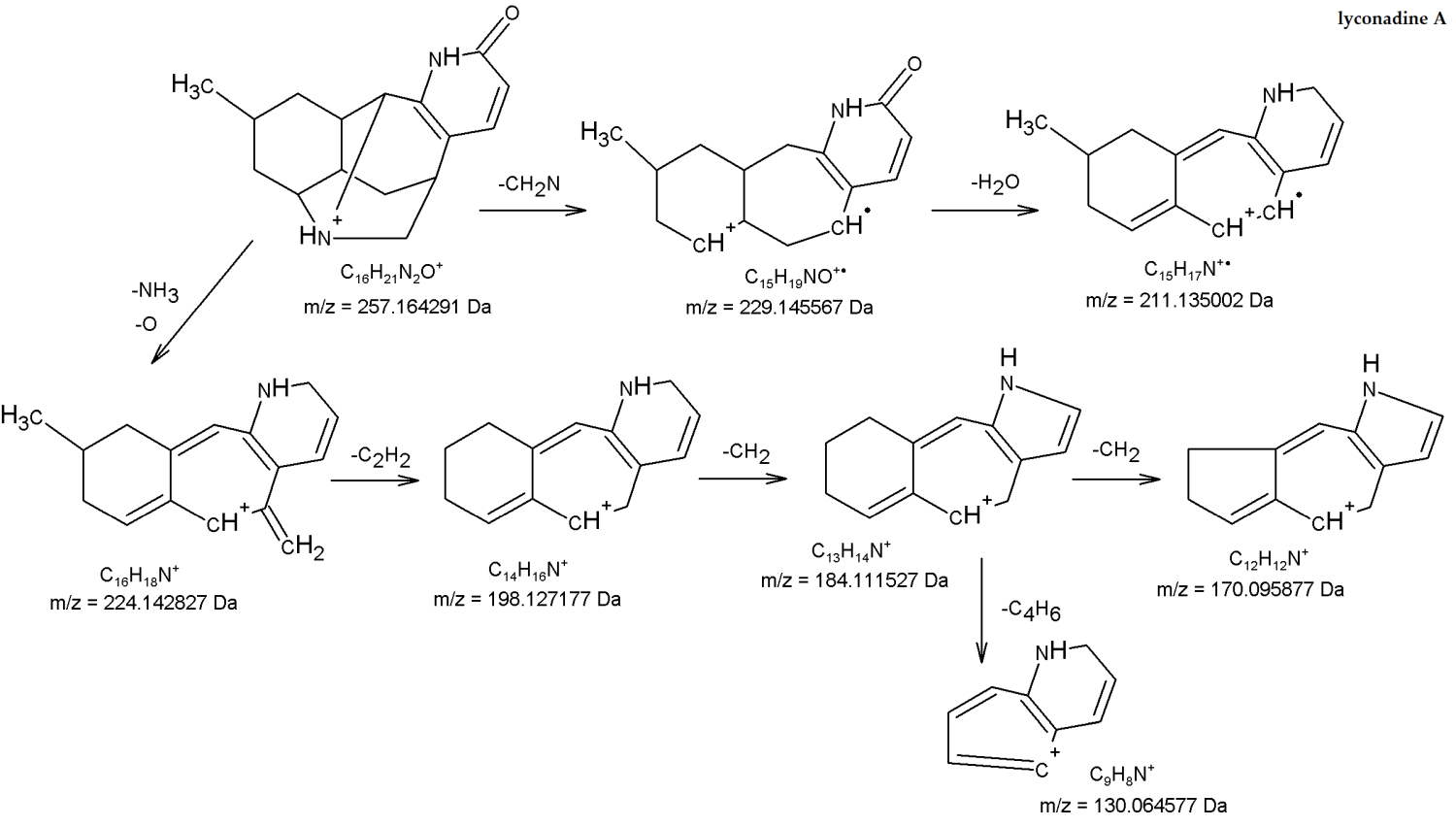

Supplement: Supplementary file 1 [file molecules-26-06379-s001.zip › Fig.S39.b.tif]

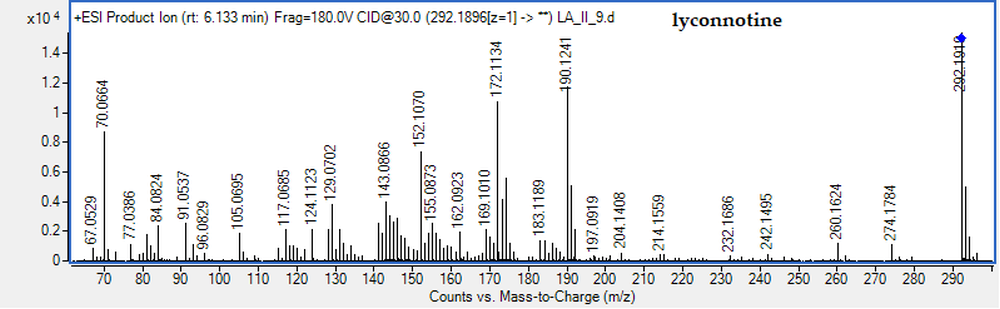

Supplement: Supplementary file 1 [file molecules-26-06379-s001.zip › Fig.S40.a.tif]

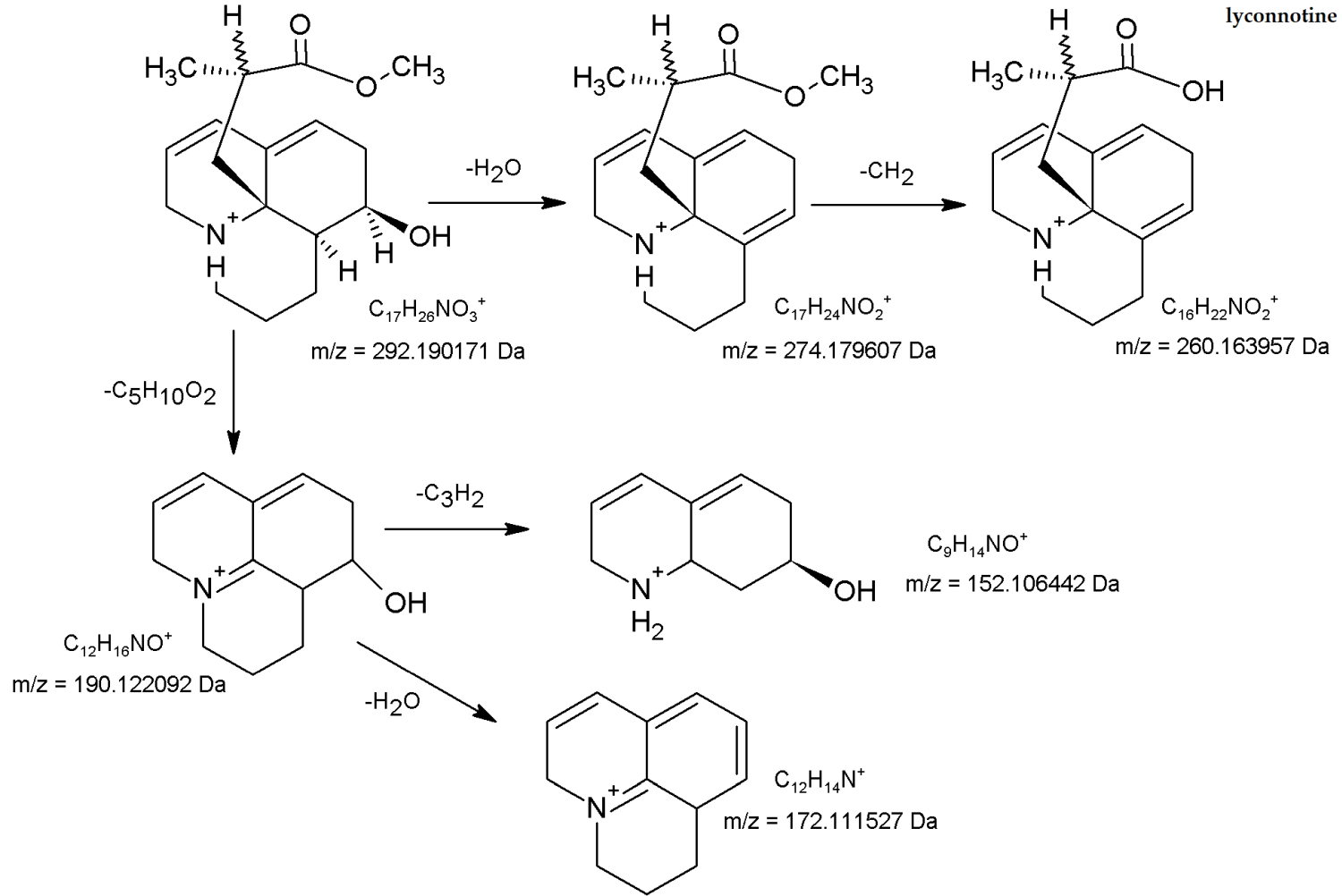

Supplement: Supplementary file 1 [file molecules-26-06379-s001.zip › Fig.S40.b.tif]

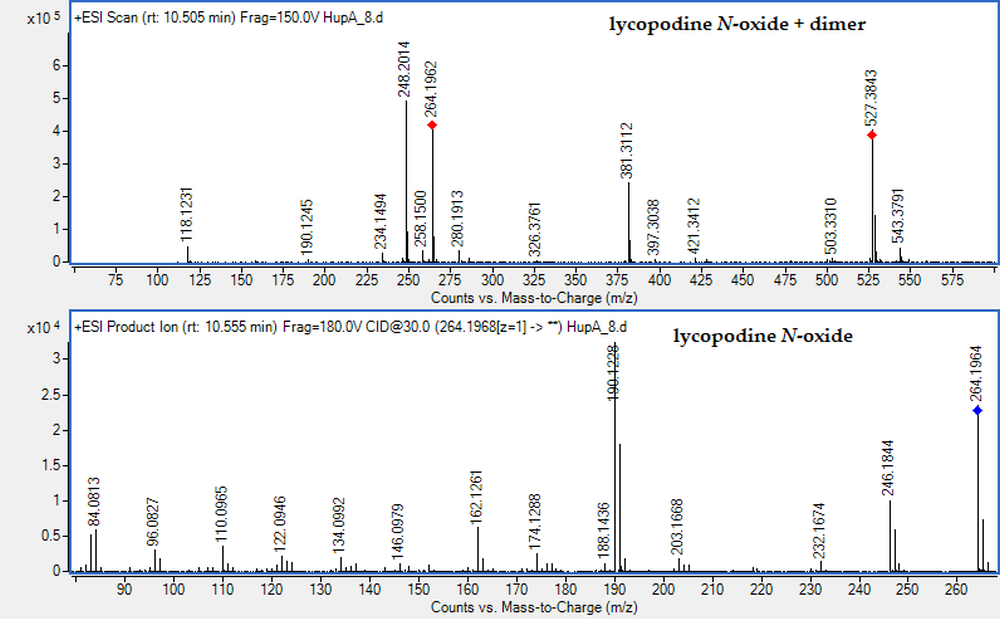

Supplement: Supplementary file 1 [file molecules-26-06379-s001.zip › Fig.S41.a.tif]

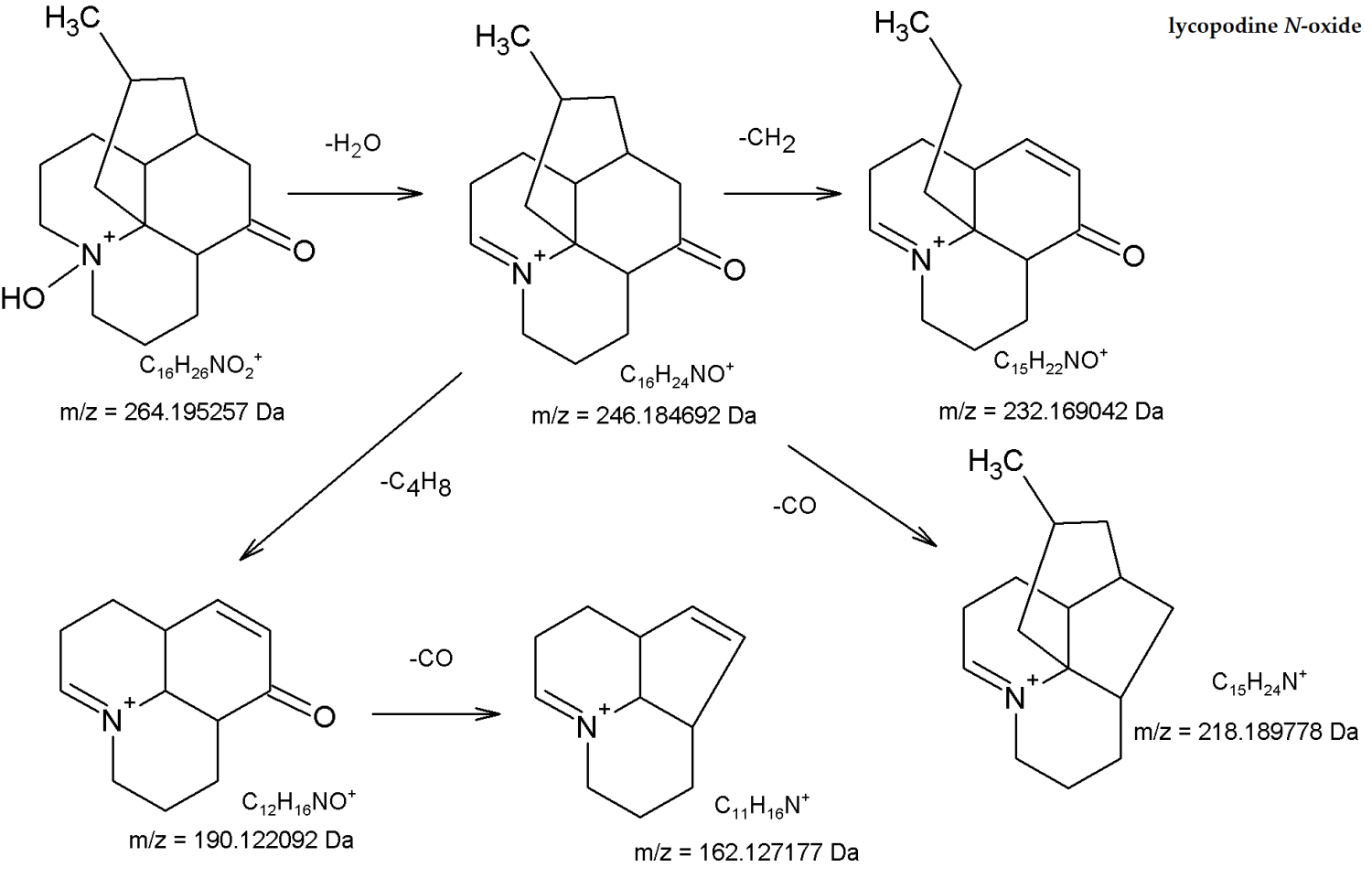

Supplement: Supplementary file 1 [file molecules-26-06379-s001.zip › Fig.S41.b.tif]

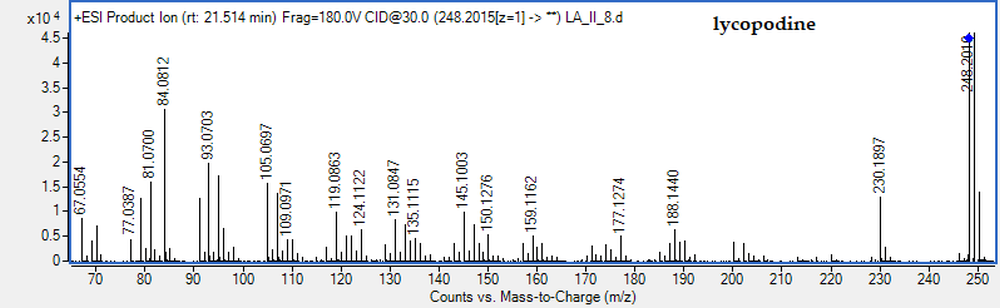

Supplement: Supplementary file 1 [file molecules-26-06379-s001.zip › Fig.S42.a.tif]

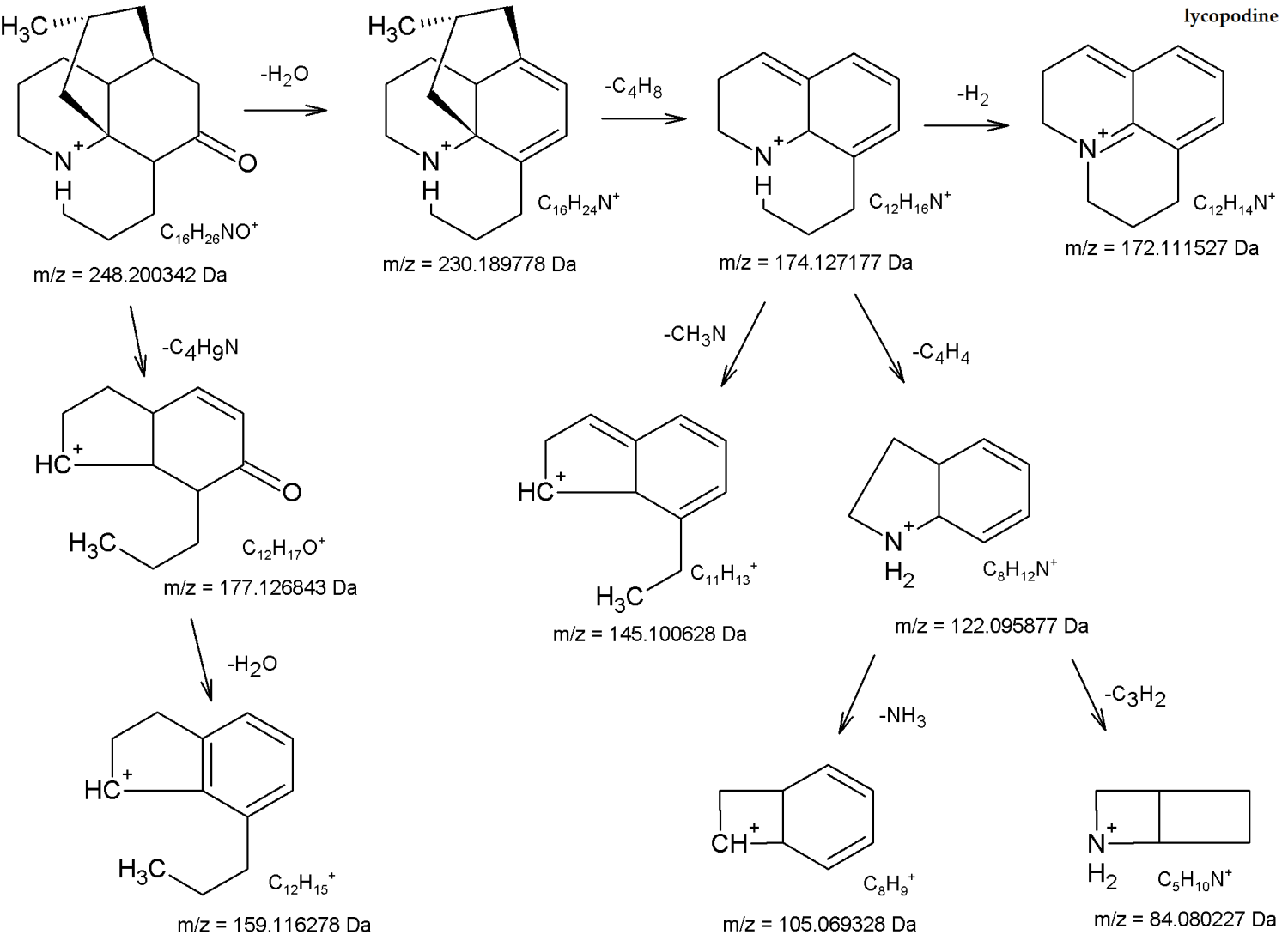

Supplement: Supplementary file 1 [file molecules-26-06379-s001.zip › Fig.S42.b.tif]

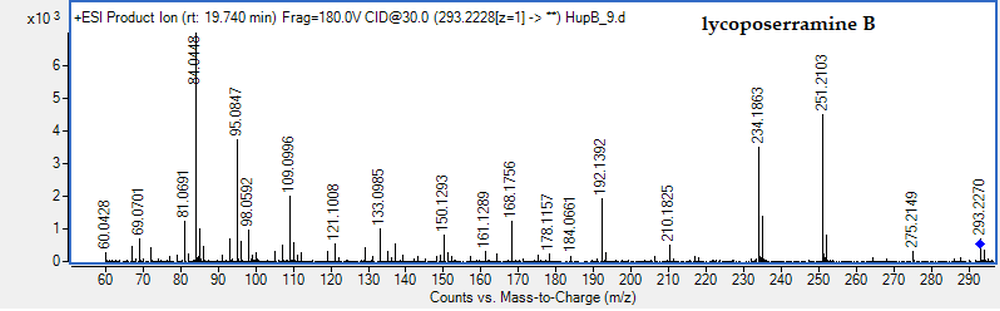

Supplement: Supplementary file 1 [file molecules-26-06379-s001.zip › Fig.S43.a.tif]

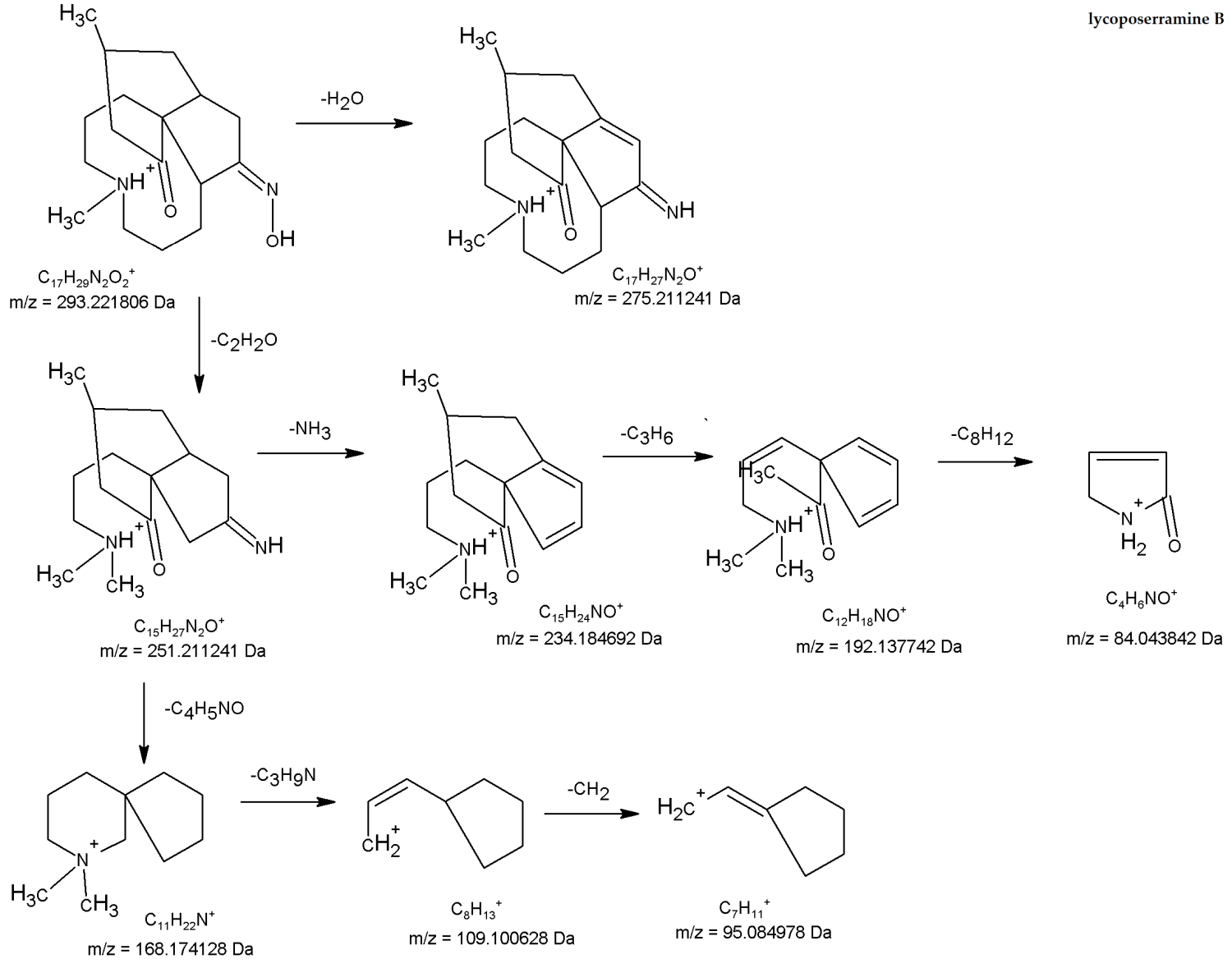

Supplement: Supplementary file 1 [file molecules-26-06379-s001.zip › Fig.S43.b.tif]

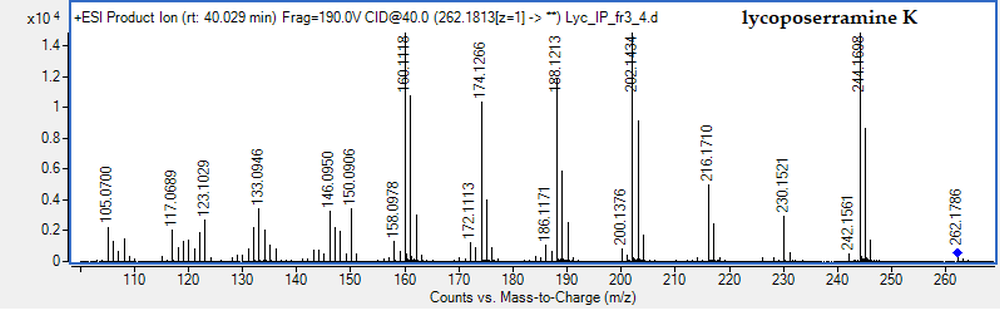

Supplement: Supplementary file 1 [file molecules-26-06379-s001.zip › Fig.S44.a.tif]

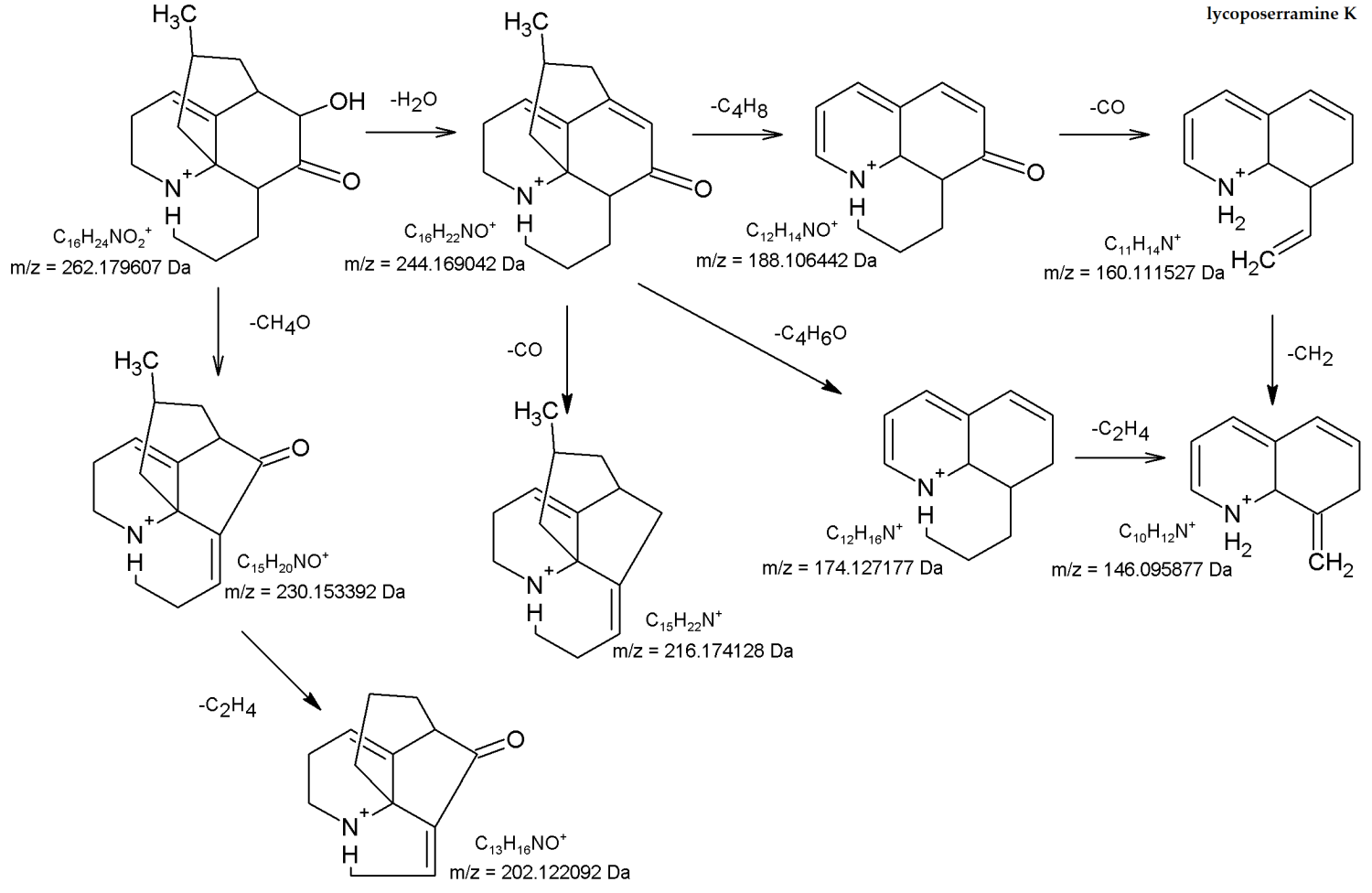

Supplement: Supplementary file 1 [file molecules-26-06379-s001.zip › Fig.S44.b.tif]

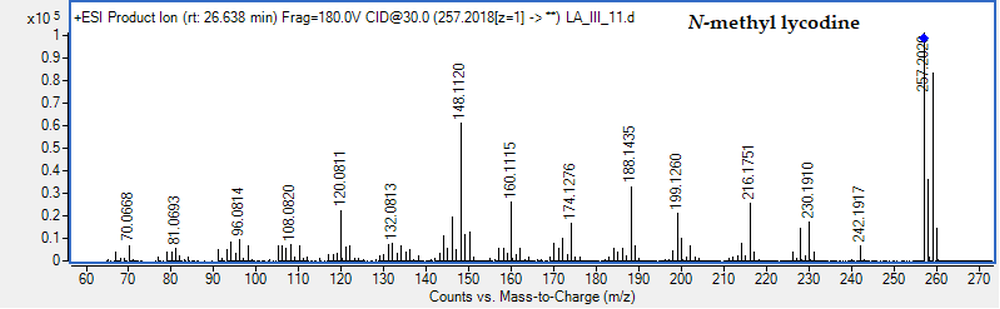

Supplement: Supplementary file 1 [file molecules-26-06379-s001.zip › Fig.S45.a.tif]

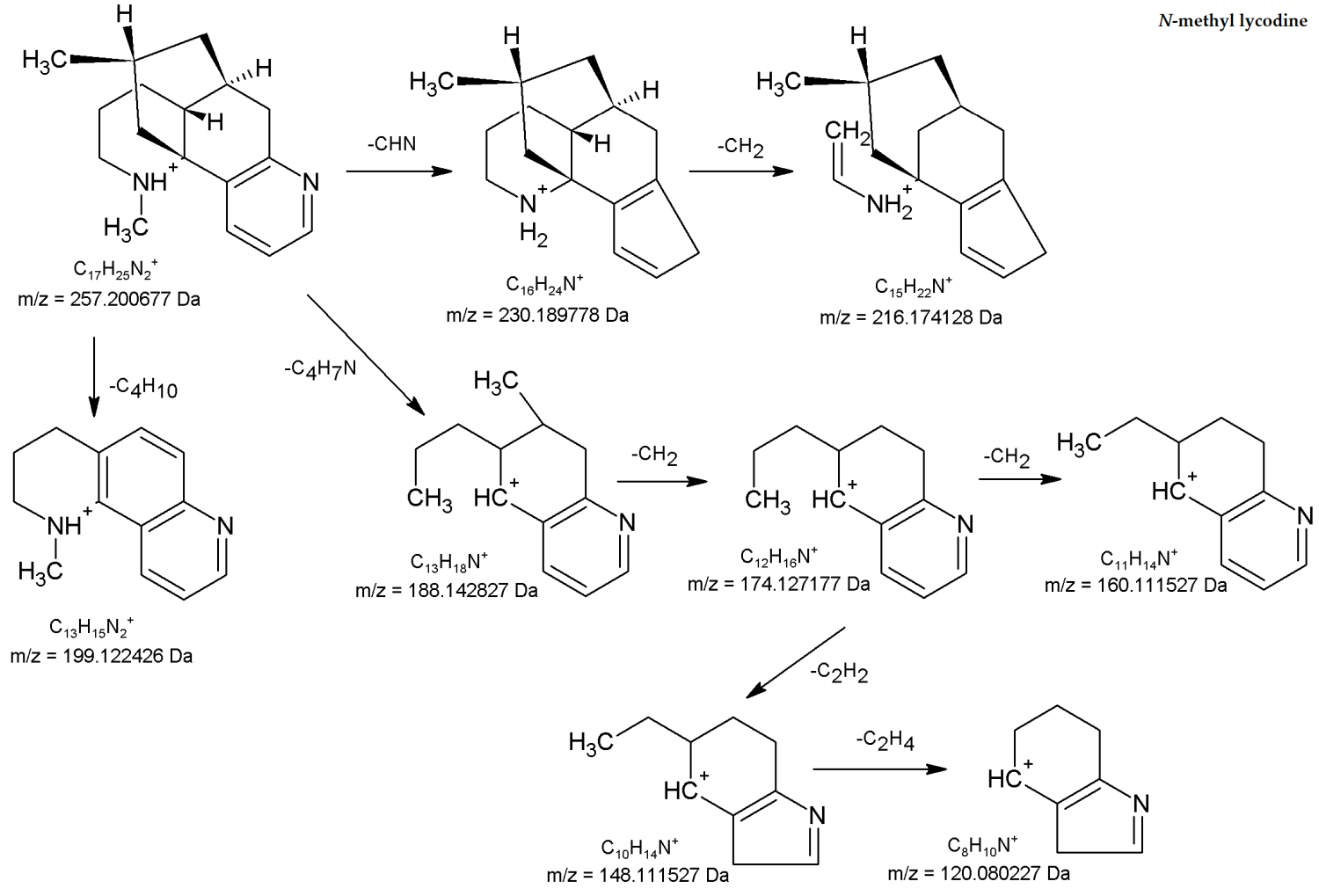

Supplement: Supplementary file 1 [file molecules-26-06379-s001.zip › Fig.S45.b.tif]

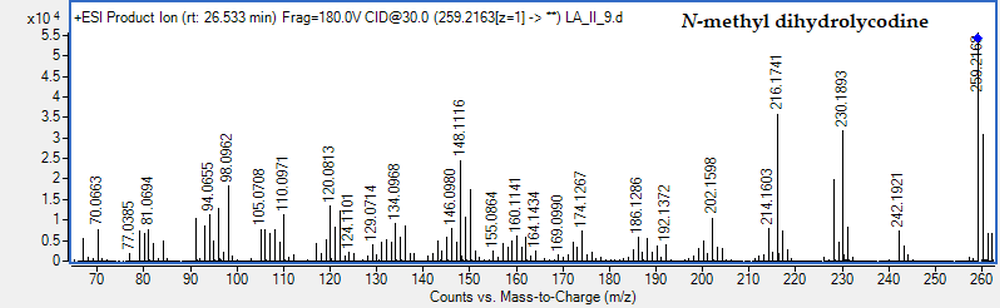

Supplement: Supplementary file 1 [file molecules-26-06379-s001.zip › Fig.S46.a.tif]

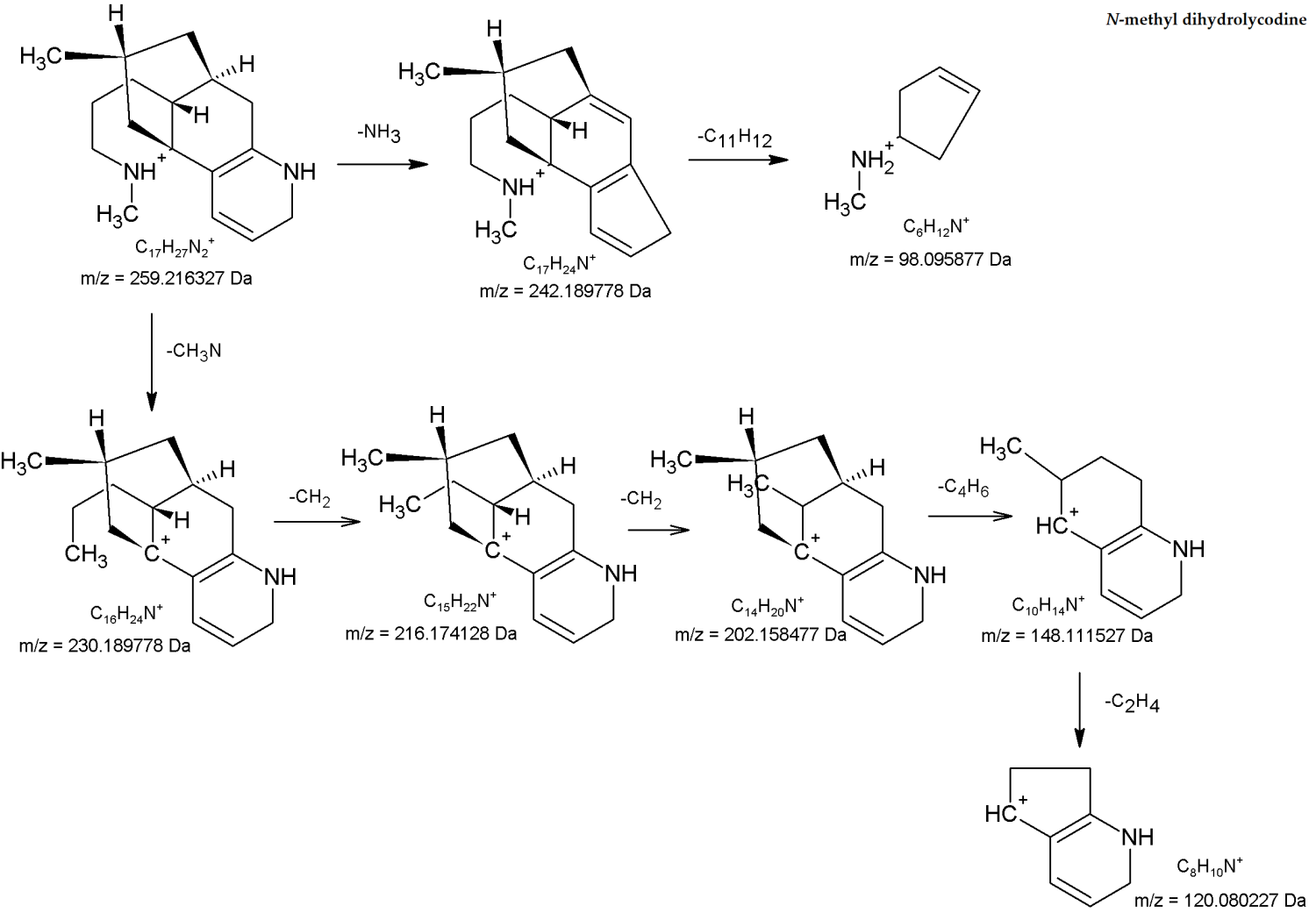

Supplement: Supplementary file 1 [file molecules-26-06379-s001.zip › Fig.S46.b.tif]

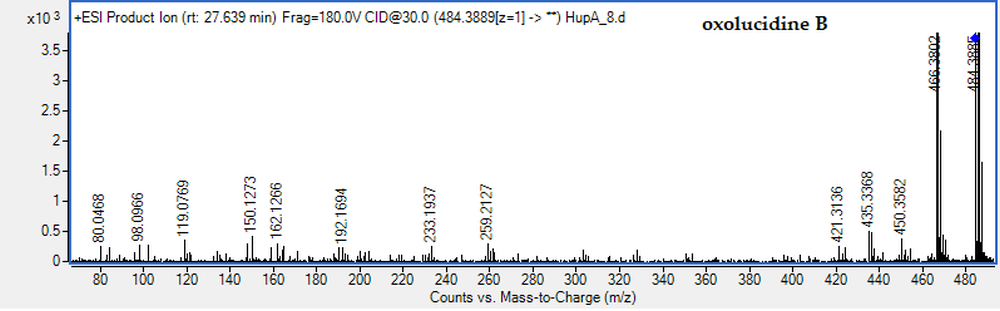

Supplement: Supplementary file 1 [file molecules-26-06379-s001.zip › Fig.S47.a.tif]

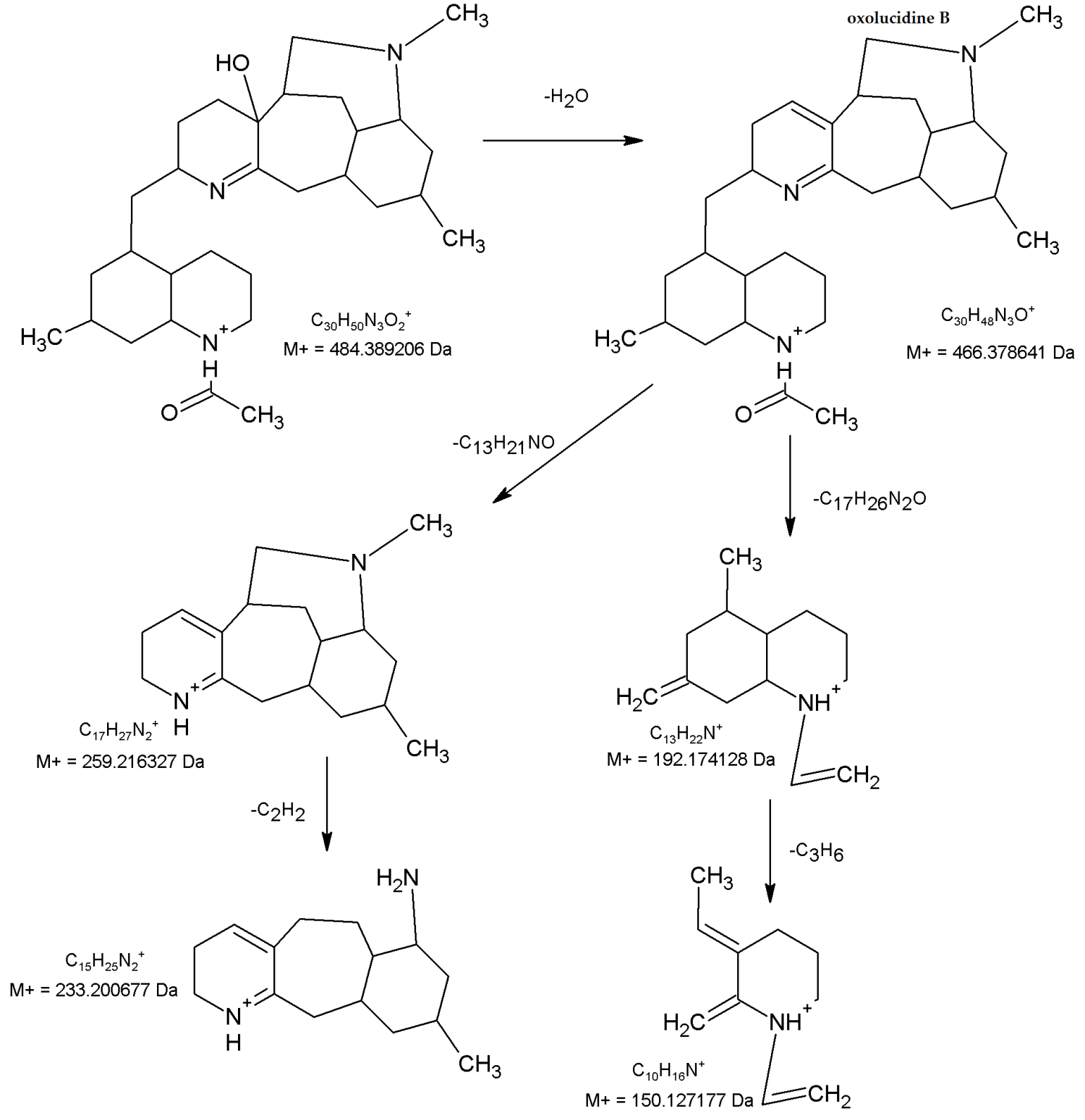

Supplement: Supplementary file 1 [file molecules-26-06379-s001.zip › Fig.S47.b.tif]

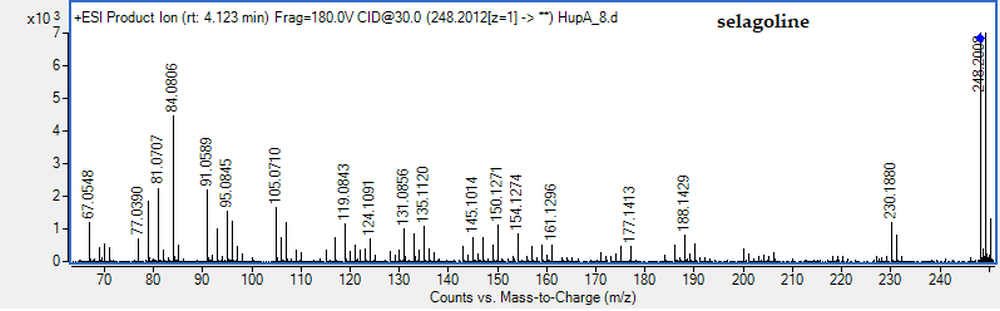

Supplement: Supplementary file 1 [file molecules-26-06379-s001.zip › Fig.S48.a.tif]

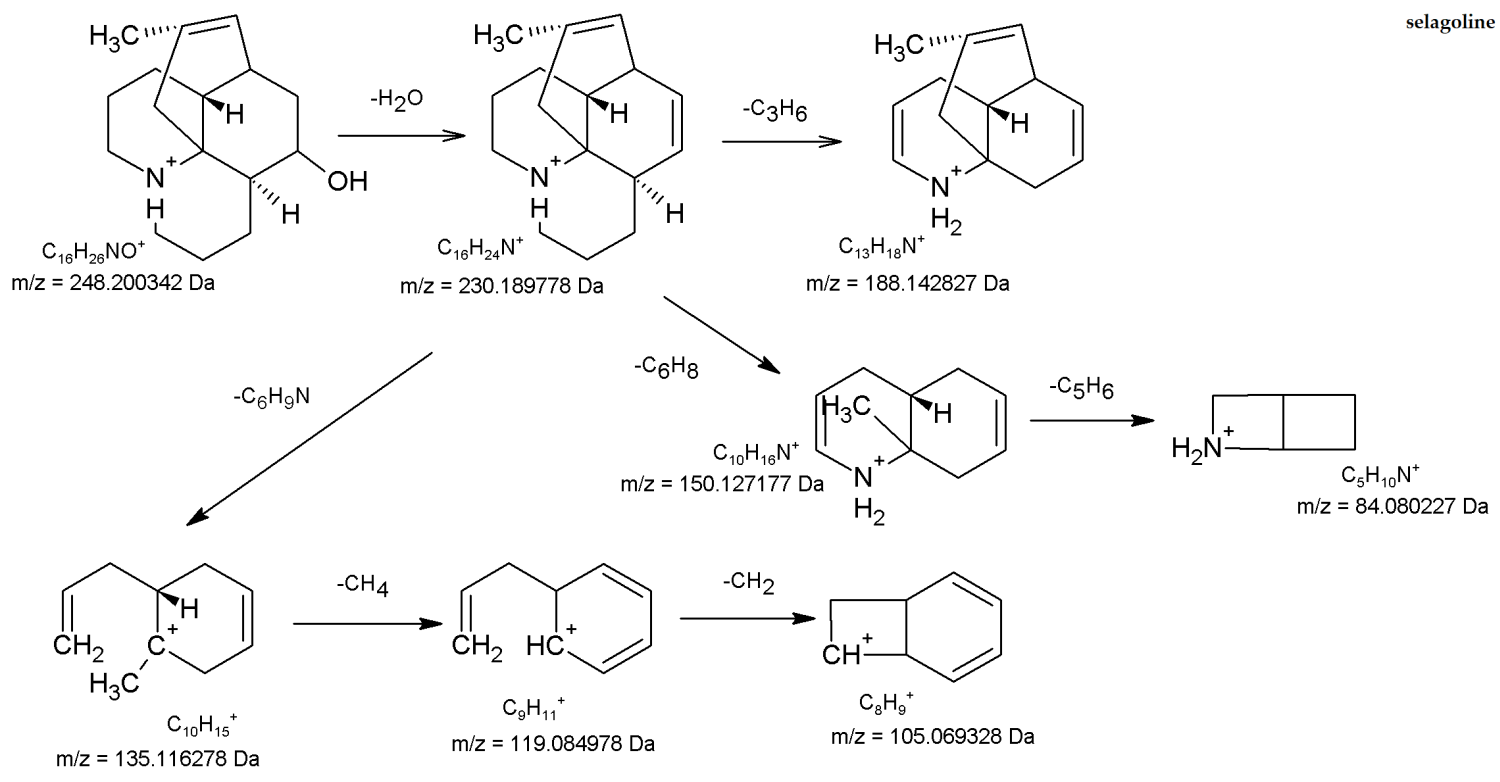

Supplement: Supplementary file 1 [file molecules-26-06379-s001.zip › Fig.S48.b.tif]

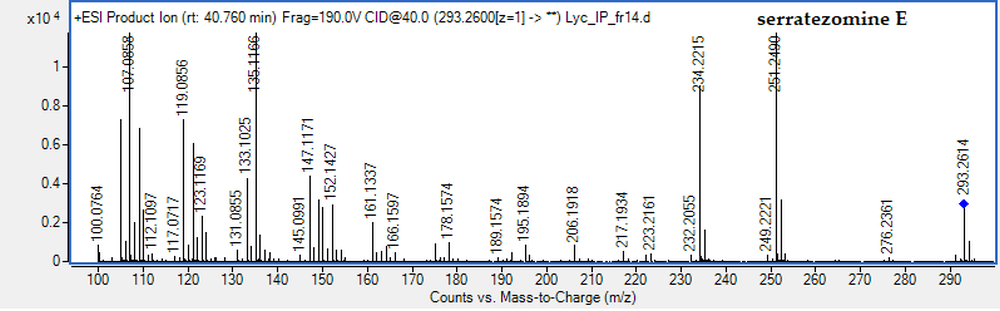

Supplement: Supplementary file 1 [file molecules-26-06379-s001.zip › Fig.S49.a.tif]

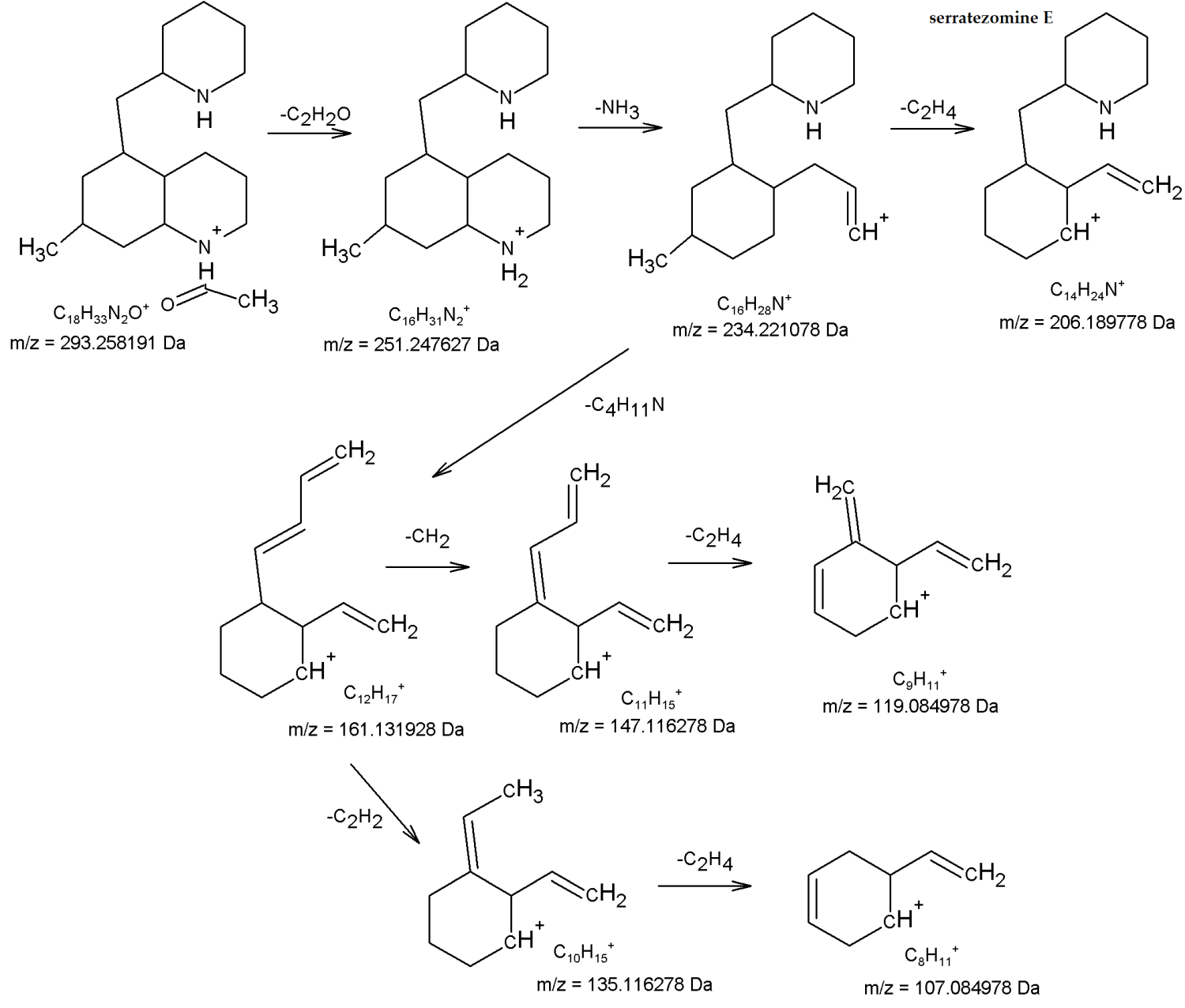

Supplement: Supplementary file 1 [file molecules-26-06379-s001.zip › Fig.S49.b.tif]

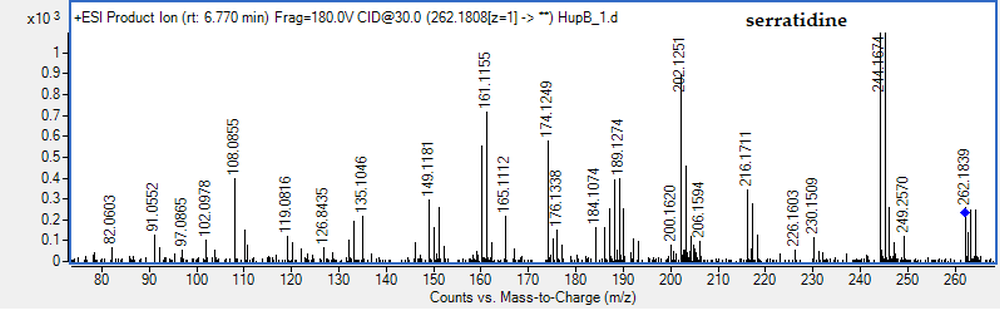

Supplement: Supplementary file 1 [file molecules-26-06379-s001.zip › Fig.S50.a.tif]

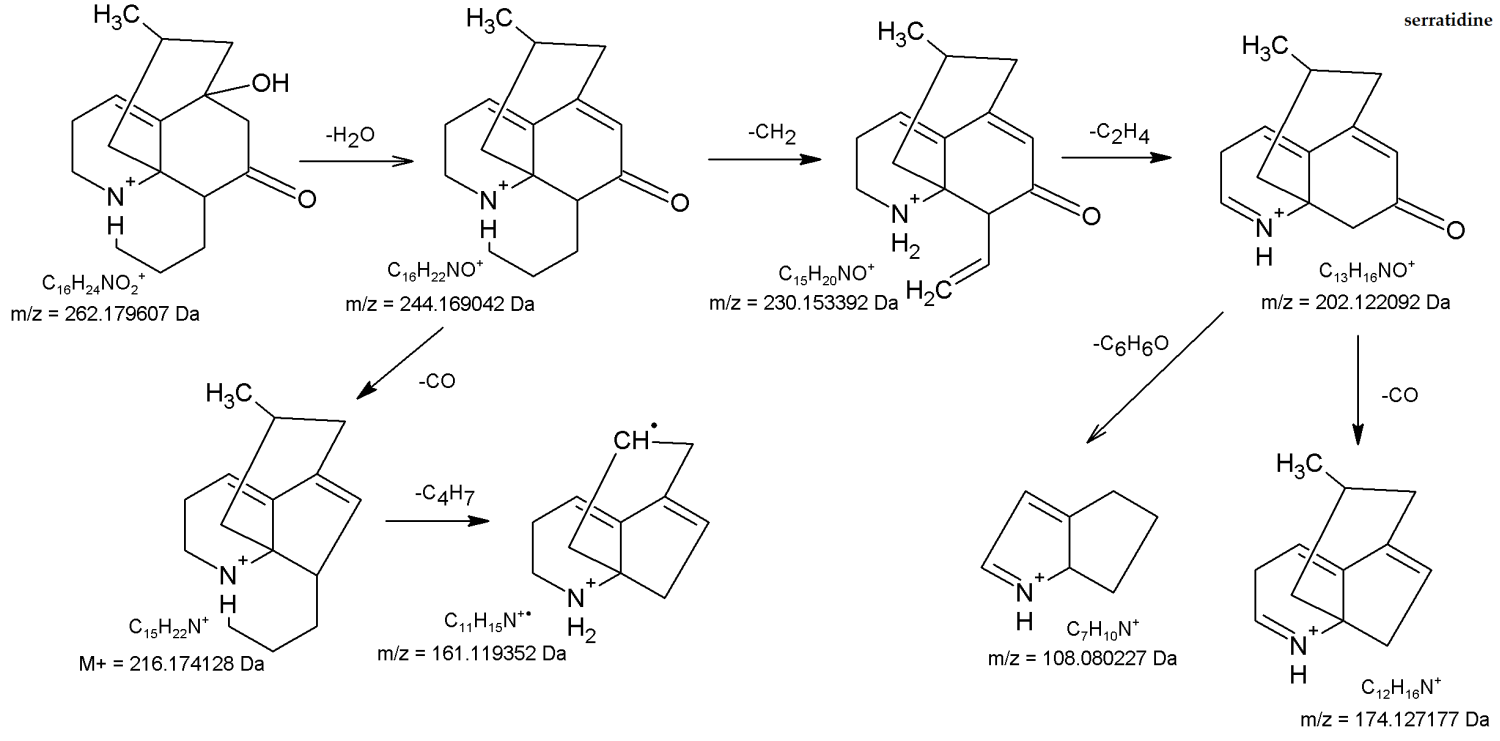

Supplement: Supplementary file 1 [file molecules-26-06379-s001.zip › Fig.S50.b.tif]

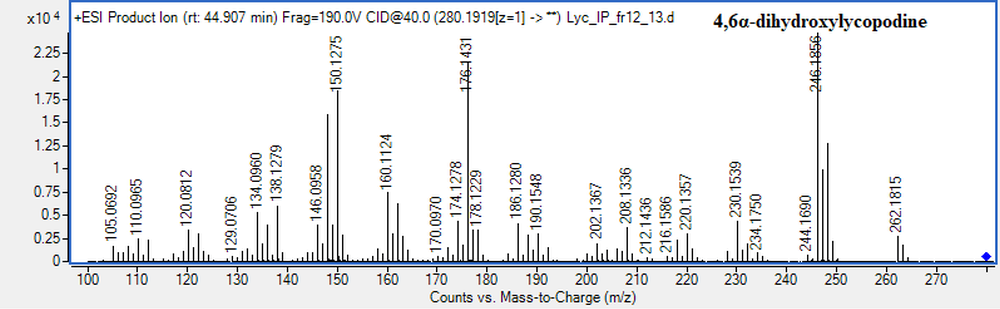

Supplement: Supplementary file 1 [file molecules-26-06379-s001.zip › Fig.S1.a.tif]

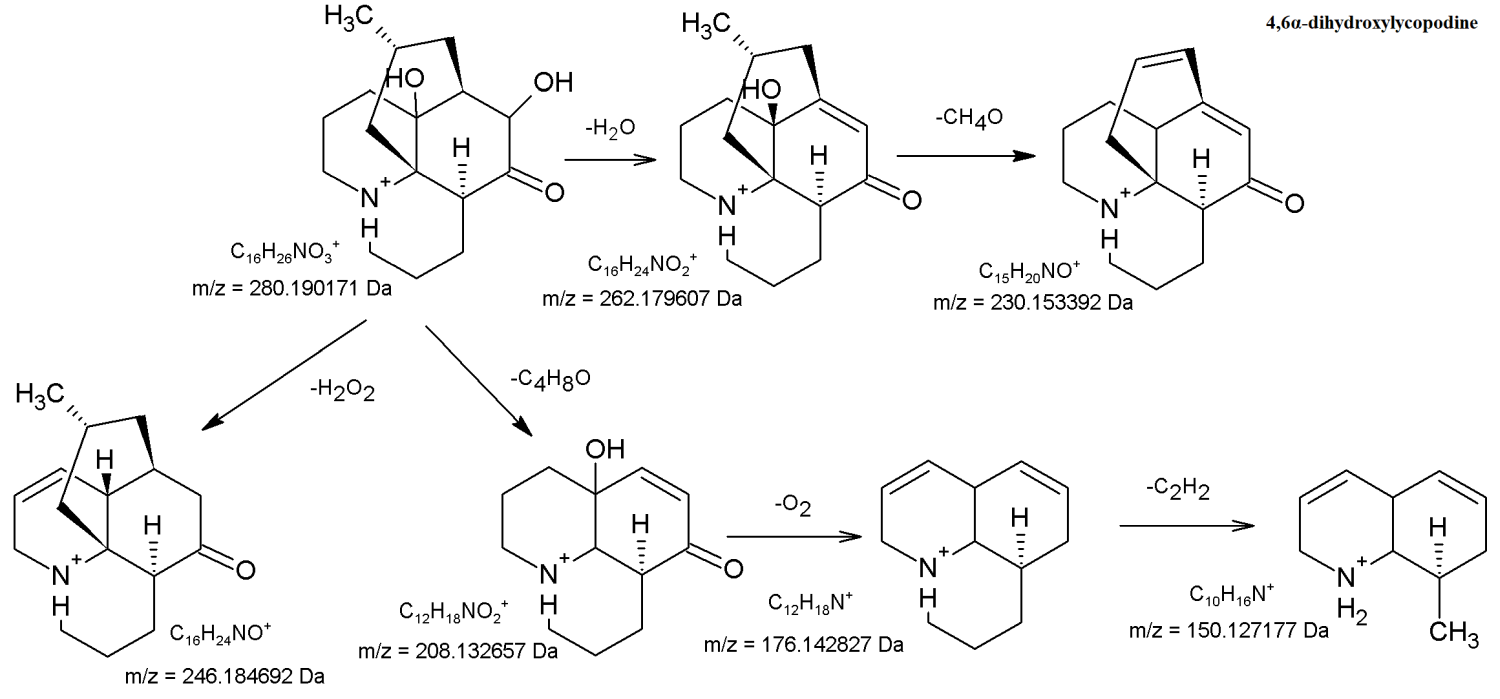

Supplement: Supplementary file 1 [file molecules-26-06379-s001.zip › Fig.S1.b.tif]
